# Mobile Units for Integrated Addiction and HIV Prevention Services for People Who Inject Drugs: The HPTN 094 Randomized Clinical Trial

**DOI:** 10.1001/jamapsychiatry.2026.3644

**Published:** 2026-11-04

**Authors:** Steven Shoptaw, Nabila El-Bassel, Brett Hanscom, Timothy Skalland, Cecile Denis, David Goodman-Meza, Irene Kuo, Jordan E. Lake, Ellen A.B. Morrison, Amy M. Richards, Jessica M. Fogel, Paul Richardson, Mark A. Marzinke, William Clarke, Rahul PaulChoudhury, Jayla Harris-Wisecarver, Melissa Cummings, Redonna Chandler, Dale R. Burwen, Philip Andrew

**Affiliations:** 1 Department of Family Medicine, University of California, Los Angeles; 2 School of Social Work, Columbia University, New York, New York; 3 Vaccine and Infectious Disease Division, Fred Hutchinson Cancer Center, Seattle, Washington; 4 Department of Psychiatry, Perelman School of Medicine, University of Pennsylvania, Philadelphia; 5 Kirby Institute, University of New South Wales, Sydney, New South Wales, Australia; 6 Milken Institute School of Public Health Department of Epidemiology, George Washington University, Washington, DC; 7 UTHealth-Houston, Houston, Texas; 8 ICAP at Columbia University, Mailman School of Public Health, New York, New York; 9 Department of Pathology, Johns Hopkins University School of Medicine, Baltimore, Maryland; 10 Statistical Center for HIV/AIDS Research, Fred Hutchinson Cancer Center, Seattle, Washington; 11 FHI 360, Durham, North Carolina; 12 Previously at the National Institute on Drug Abuse, National Institutes of Health, Rockville, Maryland; 13 Previously at the Division of AIDS, National Institute of Allergy and Infectious Diseases, National Institutes of Health, Rockville, Maryland; 14 Now at the Division of Clinical Innovation, National Center for Advancing Translational Sciences, National Institutes of Health, Rockville, Maryland

## Abstract

**Importance:**

Integrated models to deliver HIV preexposure prophylaxis (PrEP) and medication for opioid use disorder (MOUD) for people who inject drugs are needed.

**Objective:**

To determine whether integrated PrEP and MOUD services delivered on a mobile unit with manualized peer navigation improved PrEP and MOUD use compared with peer navigation to existing community-based services.

**Design, Setting, and Participants:**

This 2-group, open-label, randomized clinical trial was conducted between May 2021 and September 2024 in Houston, Texas; Los Angeles, California; New York, New York; Philadelphia, Pennsylvania; and Washington, DC, among people aged 18 years and older currently injecting drugs, without HIV, with OUD, and not taking MOUD but willing to consider MOUD.

**Interventions:**

The intervention arm (n = 203) consisted of 26 weeks of manualized peer navigation plus integrated PrEP, MOUD, and limited clinical and supportive services delivered on a mobile unit. The active control arm (n = 206) consisted of 26 weeks of manualized peer navigation to available community-based PrEP, MOUD, harm reduction, and supportive services.

**Main Outcomes and Measures:**

The co–primary outcomes were PrEP use and MOUD use at 26 weeks. Secondary outcomes included PrEP and MOUD use at 52 weeks, HIV incidence, and all-cause mortality.

**Results:**

Of 842 people who inject drugs who were screened, 409 were randomized: 134 (32.8%) female; 146 (11.2%) aged 30 years and younger vs older than 30 years. At the 26-week visit, the intervention and active control arms were similar for confirmed PrEP use: 11/203 (5.4%) vs 6/206 (2.9%) and MOUD use: 30/203 (14.8%) vs 22/206 (10.7%), respectively. At the 52-week visit, intervention and active control arms were similar for confirmed PrEP and MOUD: 4/203 (2.0%) vs 4/206 (1.9%) and MOUD use: 27/203 (13.3%) vs 29/206 (14.1%). One HIV acquisition occurred in the intervention and 2 in the active control arms; HIV incidence rate of 0.9 per 100 person-years (95% CI, 0.2-2.6). Four deaths were confirmed in the intervention arm and 13 in the active control arm (hazard ratio, 0.29; 95% CI, 0.1-0.9; *P* = .03).

**Conclusions and Relevance:**

In this randomized clinical trial, mobile delivery of integrated HIV prevention, addiction, and navigation services did not improve primary outcomes beyond peer navigation alone. The significantly lower all-cause death rate at 52 weeks in the intervention arm bears replication for delivering integrated PrEP and MOUD services to people who inject drugs.

**Trial Registration:**

ClinicalTrials.gov Identifier: NCT04804072

## Key Points

**Question:**

Does providing 26 weeks of integrated HIV prevention and addiction services for people who inject drugs using peer navigation delivered in a mobile unit produce superior outcomes compared to peer navigation to existing standard-of-care community-based services alone?

**Findings:**

In this randomized clinical trial, HIV prevention and addiction outcomes were similar between study arms, though the intervention showed a greater reduction in all-cause mortality compared with standard of care.

**Meaning:**

The findings suggest that integrated mobile service delivery models may reduce all-cause mortality for people who inject drugs.

## Teaser

This randomized clinical trial evaluates the use of preexposure prophylaxis for HIV and medications for opioid use disorder among individuals who inject drugs.

## Introduction

Opioid use disorder (OUD) is a major driver of HIV acquisition in the US^1^ and a leading contributor to mortality among people who use opioids, whether alone or in combination with stimulants.^2^ Beyond HIV acquisition and overdose,^3^ people who inject drugs experience elevated risks of comorbid conditions, including hepatitis C, bacterial infections related to nonsterile injection practices, injection-related wounds, and other sexually transmitted infections.^4^

People who inject drugs face substantial barriers to accessing key services and support necessary for consistent engagement in HIV prevention and addiction treatment. These barriers include poverty, unstable housing, food insecurity, and limited access to health care, all of which impede regular use of HIV preexposure prophylaxis (PrEP) and medication for OUD (MOUD).^5^ Siloed medical and addiction treatment systems further restrict access by fragmenting care for primary health services, HIV PrEP, and MOUD.^7^

Mobile units offer a way to address multiple barriers faced by people who inject drugs in accessing integrated services, including HIV prevention, MOUD, primary care, and social services.^8^ Mobile unit-based care differs from care based in conventional brick-and-mortar settings for people who inject drugs along key features of location, integration, immediacy, and service proximity. Yet, rigorous evaluation of mobile unit-based interventions for people who inject drugs remains limited.^9^ This study tests whether advantages of integrated HIV and MOUD services using a mobile unit combined with peer navigation would produce statistically significant improvements in primary outcomes of PrEP and MOUD compared to peer navigation to existing standard of care, community-based services alone for people who inject drugs in 5 US cities.

## Methods

### Trial Design and Oversight

HPTN 094 was a 2-arm, individually randomized, controlled, open-label study^7^ that evaluated whether an integrated intervention delivered in a mobile unit improved use of HIV PrEP and MOUD at 26 and 52 weeks (Figure 1). The trial protocol and statistical analysis plan are in Supplement 1. A single institutional review board, Advarra, provided oversight of the research. Biannual data monitoring oversight was provided by an independent study monitoring committee at the HPTN. All participants provided written informed consent. The study followed the Consolidated Standards of Reporting Trials (CONSORT) reporting guideline.^10^

**Figure 1.**
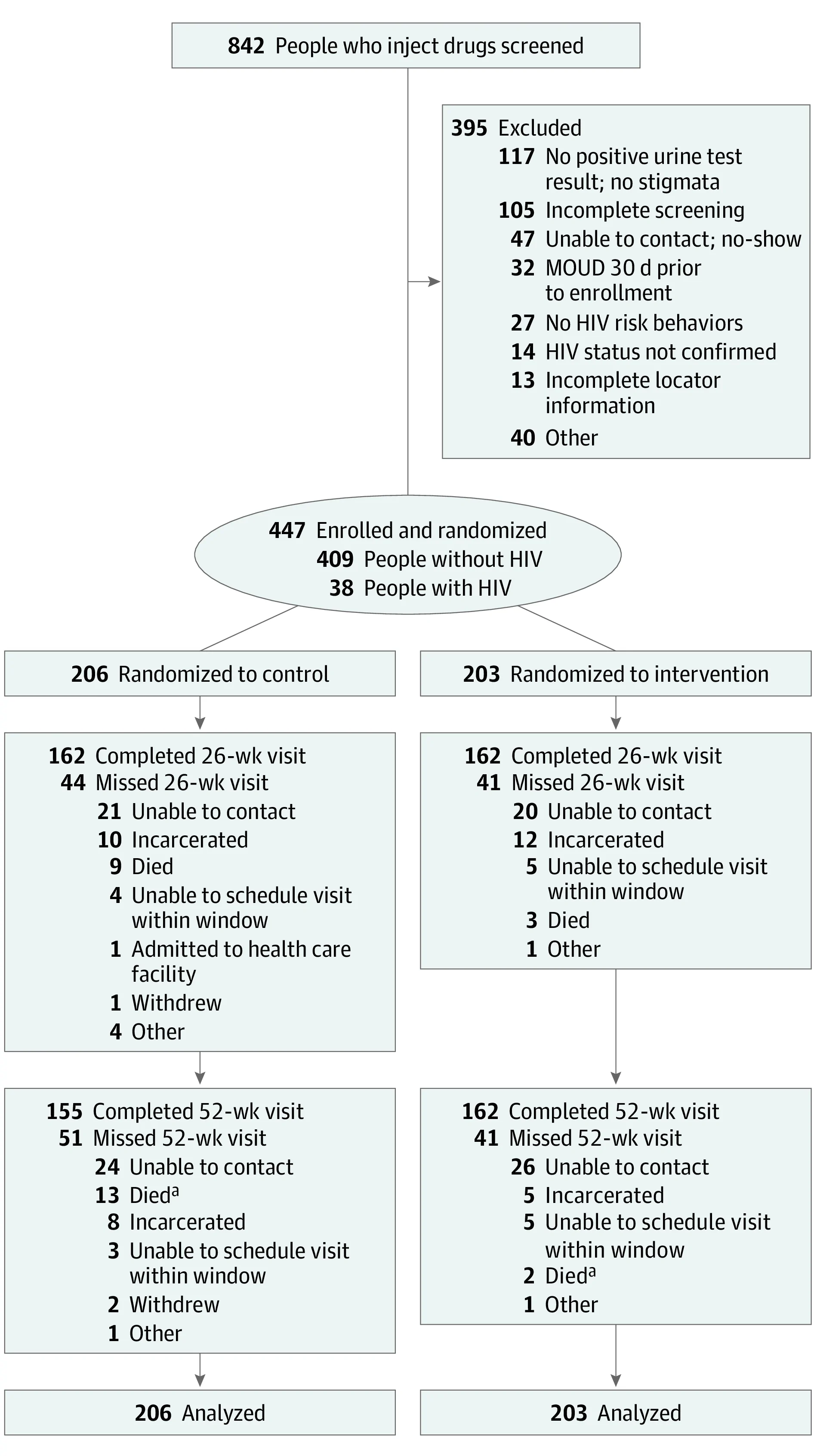
CONSORT Diagram ^a^At week 52, the total number of people who died is cumulative since enrollment.

### Participants

Eligibility criteria included current injection drug use, being aged 18 years and older, the ability to provide informed consent, not currently using but willing to use MOUD, self-reported sharing injection equipment, or having condomless sex in the last 3 months with people with HIV or persons of unknown HIV serostatus, and being able and willing to provide adequate contact information of a person or persons who could help locate participants for follow-up visits.

Participants were excluded from the study if they self-reported MOUD in the past 30 days as part of a treatment program or if they were enrolled in another interventional study. Although people with HIV were recruited and randomized, enrollment numbers were insufficient to support rigorous analysis in that group.

### Study Sites

HPTN 094 was conducted in Houston, Texas; Los Angeles, California; New York, New York; Philadelphia, Pennsylvania; and Washington, DC. The cities represented diverse large urban contexts with substantial HIV and overdose risks among people who inject drugs. Study sites were selected based on prior experience conducting research on HIV prevention and addiction for people who inject drugs, on geographic diversity, and on HIV prevalence in people who inject drugs.

### Mobile Units

Los Angeles, Houston, and New York chose identical 33-ft medical units that contained an examination table, space for processing laboratory samples, and a toilet; Philadelphia and Washington, DC, chose 26-ft, similarly equipped medical units.^7^ Data collection during screening, enrollment, week 26, and week 52 occurred in the mobile unit for all participants.

### Mobile Unit Placement

Preimplementation activities prior to study start-up were conducted by the research team in each city to develop data-driven, community-focused maps using HIV and overdose data, and the location of available community health care services to identify geographic locations where people who inject drugs might best access the mobile units.^11^ Locations were intended to facilitate access for actively injecting people who use drugs who lacked a source of care.

### Recruitment

Participants were recruited through street-based outreach at mobile unit locations, community-based recruitment events, incentivized peer referral, and social media. Purposive methods were used to compile a sample with preset target percentages of 25% women and 25% younger (<30 years) participants. Total compensation for completing all research visits was $175 in Houston; $200 in New York, $210 in Philadelphia, $350 in Los Angeles, and $360 in Washington, DC.

### Screening

Screening involved assessments to determine eligibility, including confirmation of OUD diagnosis (clinical interview and *DSM-5* checklist), active drug injection activity (recent injection marks), HIV status (rapid antibody point-of-care tests and laboratory-based confirmation), and recent opioid use (urine point-of-care test for opioids; laboratory-based test for fentanyl).

### Intervention Arm

Participants assigned to the intervention arm received 26 weeks of integrated HIV, addiction, and supportive services with peer navigation facilitation of these services, all delivered in a mobile unit (eTable 1 in Supplement 2). Peer navigation sessions linked participants to available community services if desired. Medical services delivered on the unit were (1) access to oral PrEP for people without HIV with persons assigned female sex at birth receiving emtricitabine/tenofovir disoproxil fumarate; persons assigned male sex at birth receiving emtricitabine/tenofovir alafenamide (eAppendix 1 in Supplement 2). Long-acting cabotegravir for PrEP was not provided by the study; long-acting cabotegravir use was assessed at each study visit and (2) immediate access to MOUD (sublingual buprenorphine provided on-unit or by prescription; facilitated referral to methadone clinic). In addition to facilitated access to MOUD, people who inject drugs with polysubstance use involving stimulants (methamphetamine, amphetamine, cocaine) were referred to local stimulant treatment and 12-step programs.

### Active Control Arm

Because a uniform standard for delivering integrated PrEP and MOUD to people who inject drugs who are out of treatment is not consistently available, the active control arm received 26 weeks of manualized peer navigation to facilitate linking participants to available services at brick-and-mortar agencies. The scope of services identified and referred in the active control arm paralleled services provided to the intervention arm in the mobile unit. The active control arm provided an ethically acceptable and clinically beneficial comparison condition to no navigation or to no referrals^12^ Peer navigation services were delivered virtually or in community spaces away from the mobile unit, such as in parks or near food banks and fast food restaurants.^11^

### Peer Navigation

Peer navigators were hired locally and had personal lived experience of OUD, including injection drug use, or had shared community background involving addiction and recovery with people who inject drugs. The published peer navigation model was informed by systems, self-determination, empowerment, shared decision-making, and social support theories of behavior change^13^ (eAppendix 2 in Supplement 2). A reference manual for supervisors based on the model-guided navigation sessions aided implementation across sites.^15^ Fidelity-supporting procedures for using the navigation model included peer navigators completing 2 weeks of online and in-person training on the model and the study prior to delivering services. Peer navigators provided services to participants in both arms and were supervised by a senior staff member at each site. Peer navigation supervisors from each site met monthly with an expert with extensive experience in training and supervising peer navigators to monitor adherence to the peer navigation model and to support navigators in monitoring their own recovery journeys.

Peer navigators met with participants, established rapport, conducted needs assessments, and made referrals to and assisted in accessing services. All participants completed 5 required sessions: (1) MOUD and harm reduction; (2) HIV PrEP; (3) barriers and facilitators to integrated health care; (4) housing, food security, hygiene; and (5) sexual risk behaviors. Additional sessions included these topics: (1) injection risk reduction/drug splitting; (2) stimulants and other substances; (3) alcohol, benzodiazepines, and other downers; and (4) depression and stigma. Dose of peer navigation was measured by number of sessions completed (required and elective), session length (in minutes), and session topics. After 26 weeks, no peer navigation services were provided for either arm.

### Study Assessments

Self-reported demographic characteristics (age, sex assigned at birth, race, ethnicity) and behavioral variables (housing status, access to health care, mental health, criminal justice engagement, drug and alcohol use, overdose experiences, drug treatment experience, and personal connections and support) were captured on case report forms at enrollment, week 26, and week 52. Sensitive information was collected via computer-assisted self-interview, including sexual behavior, medication adherence, stigma, violence, and sexual abuse. Reported deaths were verified by death certificate or medical examiner report, when feasible.

### Laboratory Methods

Biomarkers of recent drug use at screening and follow-ups were measured via antibody-based urine dipsticks that assessed organic and synthetic opioids, oxycodone, cocaine, methamphetamine, amphetamine, and benzodiazepines. Urine screens for fentanyl were conducted via local laboratories. Blood specimens were collected at weeks 0, 26, and 52 and tested in local laboratories. Plasma, urine, and dried blood spots were collected at study visits for HIV confirmatory testing, PrEP, and detection of illicit or nonprescribed drugs. A biomarker confirmed methadone testing was determined using HPTN Laboratory Center–derived urine measurements. Plasma samples were used to assess adherence to buprenorphine. Testing was performed at the HPTN Laboratory Center (Johns Hopkins University) and described in detail in eAppendix 1 in Supplement 2.

### Statistical Analysis

The study had 2 co–primary outcomes. The primary HIV prevention outcome was the proportion of enrolled participants who returned for the week 26 visit and had biomarker-verified evidence of PrEP use. The primary drug use outcome was the proportion of enrolled participants who attended the week 26 visit and had biomarker-verified evidence of MOUD use (buprenorphine or methadone) and a current prescription for buprenorphine or proof of attendance at a methadone clinic. Secondary outcomes included PrEP and MOUD use at 52-week visits, HIV incidence, overdose-related mortality, and all-cause mortality.

Sample sizes were calculated to provide 90% power to detect 15-point differences in PrEP and MOUD use at 26-week visits, with 5% type I error. Anticipating 25% MOUD use and 5% use of PrEP in the active control arm based on prior research,^16^ a sample of 216 participants was needed to confirm a 15-point difference in the percentage of PrEP use between arms (5% vs 20%), and 400 participants were needed to detect a 15-point difference in the percentage of MOUD use between arms (25% vs 40%). To ensure sufficient power for both end points, the larger of the 2 numbers (n = 400) was chosen. In planning, MOUD use was not expected to be higher than 40% in the intervention arm due to life challenges faced by these participants. Recognizing that larger proportions of use require larger sample sizes, we assumed 40% MOUD use as a conservative estimate of sample size. If use turned out to be lower, the study would still have sufficient statistical power. Given the innovative nature of this intervention, there were no prior data to inform these predictions. All enrolled participants were included in the primary analyses; no adjustment was needed to account for dropout.

Generalized linear models for binary outcomes using the identity link were used to estimate the differences in proportions between study arms for the primary HIV PrEP and MOUD use outcomes. All analyses were performed using SAS Version 9.4 (SAS Institute). *P* values less than .05 were considered statistically significant.

## Results

From May 2021 through September 2023, 842 people who inject drugs consented to participate in HPTN 094, with 409 eligible and enrolled (Figure 1). Participant demographic characteristics and substance use histories were similar across both study arms. Demographic, social, and drug use characteristics for participants are shown in Table 1.

**Table 1.** Demographic and Drug Use Characteristics at Enrollment

Characteristic	Participants, No. (%)		
	Active control (n = 206)	Intervention (n = 203)	Total (N = 409)
Sex at birth			
Male	138 (67.0)	137 (67.5)	275 (67.2)
Female	68 (33.0)	66 (32.5)	134 (32.8)
Age, y			
<30	26 (12.6)	20 (9.9)	46 (11.2)
≥30	180 (87.4)	183 (90.1)	363 (88.8)
Race^a^			
American Indian or Alaska Native	9 (4.4)	9 (4.4)	18 (4.4)
Black or African American	41 (19.9)	43 (21.2)	84 (20.5)
Native Hawaiian or Other Pacific Islander	2 (0.5)	0	2 (0.5)
White	115 (55.8)	111 (54.7)	226 (55.3)
Multiple races	6 (2.9)	4 (2.0)	10 (2.4)
Other^b^	33 (16.0)	35 (17.2)	68 (16.6)
Missing	0	1 (0.5)	1 (0.2)
Ethnicity^a^			
Hispanic or Latino	62 (30.1)	71 (35.0)	133 (32.5)
Not Hispanic or Latino	140 (68.0)	132 (65.0)	272 (66.5)
Not reported	3 (1.5)	0	3 (0.7)
Unknown	1 (0.5)	0	1 (0.2)
Housing at enrollment			
Private residence (own, friend, family)	62 (30.1)	65 (32.0)	127 (31.1)
Park/sidewalk, encampment, shelter	59 (28.6)	56 (27.6)	115 (28.1)
Tiny home; transitional or subsidized housing	50 (24.3)	53 (26.1)	103 (25.2)
Unhoused, unspecified	35 (17.0)	29 (14.3)	64 (15.6)
Drug use reported past year at enrollment^b^			
Buprenorphine, not prescribed	45 (21.8)	31 (15.3)	76 (18.6)
Methadone, not prescribed	42 (20.4)	43 (21.2)	85 (20.8)
Fentanyl	168 (81.6)	161 (79.3)	329 (80.4)
Opioids for pain, not prescribed	56 (27.2)	51 (25.1)	107 (26.2)
Amphetamine or methamphetamine	136 (66.0)	126 (62.1)	262 (64.1)
Cocaine	141 (68.4)	139 (68.5)	280 (68.5)
Drug use reported past 1 mo at enrollment^b^			
Buprenorphine, not prescribed	23 (11.2)	18 (8.9)	41 (10.0)
Methadone, not prescribed	28 (13.6)	26 (12.8)	54 (13.2)
Fentanyl	164 (79.6)	157 (77.3)	321 (78.5)
Opioids for pain, not prescribed	41 (19.9)	43 (21.2)	84 (20.5)
Amphetamine or methamphetamine	123 (59.7)	112 (55.2)	235 (57.5)
Cocaine	125 (60.7)	127 (62.6)	252 (61.6)
Ways usually took opioids past 1 mo at enrollment^d^			
Injection	176 (85.4)	191 (94.1)	367 (89.7)
Oral	52 (25.2)	53 (26.1)	105 (25.7)
Smoking	35 (17.0)	30 (14.8)	65 (15.9)
Snorting	20 (9.7)	14 (6.9)	34 (8.3)
Ways usually used stimulants past 1 mo^d^			
Injection	100 (48.5)	98 (48.3)	198 (48.4)
Oral	5 (2.4)	4 (2.0)	9 (2.2)
Smoking	93 (45.1)	81 (39.9)	174 (42.5)
Snorting	24 (11.7)	24 (11.8)	48 (11.7)
Self-reported overdose in the past 6 mo	81 (39.3)	76 (37.4)	157 (38.4)

^a^ Race and ethnicity data were collected via an in-person interview and reported to support comparison of study participant composition with background race and ethnicity prevalence for people who inject drugs in the 5 US cities.

^b^ Site staff interviewed participants regarding race and ethnicity, recorded responses on a case report form, and recorded “other” when participants’ preferred race was not represented on the form. The “other” option was not available for ethnicity.

^c^ Participants could have taken multiple drugs.

^d^ Participants could have taken a drug in multiple ways.

### Retention

Retention data were similar for both study arms (Figure 1). In the active control arm, 78.6% (162/206) and 79.8% (162/203) in the intervention arm completed 26-week visits. At 52 weeks, 155 of 206 participants (75.2%) in the active control arm and 162 of 203 in the intervention arm (79.8%) completed follow-up visits.

### Peer Navigation

No statistically significant differences were detected between arms for navigation sessions along number of sessions, length of sessions, or topics of sessions (Table 2). A total of 1619 sessions were conducted among participants in the active control arm, with an mean (SD) of 7.9 (7.7) sessions per participant, and 1783 sessions were conducted among participants in the intervention arm, with a mean (SD) of 8.8 (7.6) sessions per participant over 26 weeks. Navigation sessions typically lasted 6 to 30 minutes in both the active control arm (951/1619 [58.7%]) and the intervention arm (1098/1783 [61.7%]). Most navigation sessions addressed MOUD (active control arm, 1116/1619 [68.9%]; intervention arm, 1264/1783 [70.9%]), followed by PrEP (active control arm, 534/1619 [33.0%]; intervention arm, 585/1783 [32.8%]).

**Table 2.** Navigation Characteristics by Study Arm

Characteristic	People without HIV, No. (%)		
	Active control (n = 206)	Intervention (n = 203)	Total (N = 409)
Total sessions, No.^a^	1619	1783	3402
Sessions per participant, mean (SD)	7.9 (7.7)	8.8 (7.6)	8.3 (7.7)
Location of session^b^			
In person	1086 (67.1)	1367 (76.7)	2453 (72.1)
Virtual	533 (32.9)	416 (23.3)	949 (27.9)
Length of sessions, min^b^			
0-5	378 (23.3)	325 (18.2)	703 (20.7)
6-30	951 (58.7)	1098 (61.6)	2049 (60.2)
>30	290 (17.9)	360 (20.2)	650 (20.1)
Topic addressed^c^			
MOUD	1116 (68.9)	1264 (70.9)	2380 (70.0)
PrEP	534 (33.0)	585 (32.8)	1119 (32.9)
Other	486 (30.0)	571 (32.0)	1057 (31.1)

Abbreviations: MOUD, medications for opioid use disorder; PrEP, preexposure prophylaxis.

^a^ A total of 12 sessions occurred after the closing of week 26 allowable visit window.

^b^ Percentages for location and length of session may not sum to 100% due to rounding.

^c^ Multiple topics could be addressed during the same navigation session.

### Primary Effects on PrEP and MOUD

After 26 weeks of intervention, there were no statistically significant differences between study arms for verified PrEP use (active control arm, 6/206 [2.9%]; intervention arm, 11/203 [5.4%]; difference, 2.5 percentage points; 95% CI, −1.4 to 6.4; *P* = .20) or for confirmed MOUD engagement (active control arm, 22/206 [10.7%]; intervention arm, 30/203 [14.8%]; difference, 4.1 percentage points; 95% CI, −2.4 to 10.6; *P* = .20) (Table 3).

**Table 3.** Medications for Opioid Use Disorder (MOUD) and Preexposure Prophylaxis (PrEP) Use at Weeks 26 and 52 for People Who Inject Drugs

Medication	Week 26, No./total No. (%)		Difference, percentage points (95% CI)	*P* value	Week 52, No./total No. (%)		Difference, percentage points (95% CI)	*P* value
	Active Control	Intervention			Active Control	Intervention		
People without HIV at enrollment, No.	206	203			206	203		
MOUD biomarker and prescription^a^	22/206 (10.7)	30/203 (14.8)	4.1 (−2.4 to 10.6)	.20	29/206 (14.1)	27/203 (13.3)	−0.8 (−7.4 to 5.9)	.80
Detectable buprenorphine^b^	8/22 (36.4)	17/30 (56.7)	NA	NA	13/29 (44.8)	12/27 (44.4)	NA	NA
Detectable methadone^c^	14/22 (63.6)	15/30 (50.0)	NA	NA	18/29 (62.1)	15/27 (55.6)	NA	NA
MOUD biomarker^a^	52/206 (25.2)	53/203 (26.1)	NA	NA	57/206 (27.7)	60/203 (29.6)	NA	NA
Detectable buprenorphine^b^	12/52 (23.1)	25/53 (47.2)	NA	NA	17/57 (29.8)	21/60 (35.0)	NA	NA
Detectable methadone^c^	40/52 (76.9)	30/53 (56.6)	NA	NA	42/57 (73.7)	39/60 (65.0)	NA	NA
Self-reported currently taking MOUD	38/206 (18.4)	51/203 (25.1)	NA	NA	50/206 (24.3)	50/203 (24.6)	NA	NA
PrEP detected^d^	6 (2.9)	11 (5.4)	2.5 (−1.4 to 6.4)	.20	4 (1.9)	4 (2.0)	0.03 (−2.7 to 2.7)	.98

^a^ Participants could have detectable buprenorphine and methadone at the same visit.

^b^ Detectable buprenorphine, norbuprenorphine, or buprenorphine-glucuronide measured in urine and/or blood.

^c^ Detectable methadone or ethylenediamine-dimethyl-phosphinic acid.

^d^ Detectable tenofovir-based PrEP or long acting cabogetravir in biomedical measurements from blood.

### Secondary Outcomes

At week 52, 26 weeks after all interventions ended, 4 of 206 participants in the active control arm (1.9%) and 4 of 203 in the intervention arm (2.0%) met criteria for verified PrEP use. At 26-week visits, 2 participants, 1 in each arm, had acquired HIV, for an overall incidence rate of 1.0 per 100 person-years (95% CI, 0.1-3.7). By 52 weeks, an additional HIV acquisition occurred in the active control arm for a 52-week incidence rate of 0.9 per 100 person-years (95% CI, 0.2-2.6).

MOUD use at 52 weeks was similar between the active control (29/206 [14.1%]) and intervention arms (27/203 [13.7%]). The percentages of participants who met MOUD use criteria were similar by study arm at both 26- and 52-week visits. A minority of participants met the MOUD criteria at both visits with 9 of 206 (4.5%) in the active control arm and 13 of 203 (6.4%) in the intervention arm.

Finally, there was a non–statistically significant difference (Figure 2) in all-cause mortality between study arms through week 26, with 9 deaths confirmed in the active control arm (incidence rate, 9.5 deaths/100 person years) and 3 deaths in the intervention arm (incidence rate, 3.0 deaths/100 person-years; estimated hazard ratio, 0.33; 95% CI, 0.09-1.23; *P* = .10). By week 52, 13 deaths were confirmed in the active control arm (incidence rate, 7.3 deaths/100 person-years) and 4 deaths in the intervention arm (incidence rate, 2.1 deaths/100 person-years), a statistically significant difference and an estimated hazard ratio of 0.29 (95% CI, 0.1-0.9; *P* = .03). We observed no meaningful site-level differences in all-cause mortality (eTable 2 in Supplement 2).

**Figure 2.**
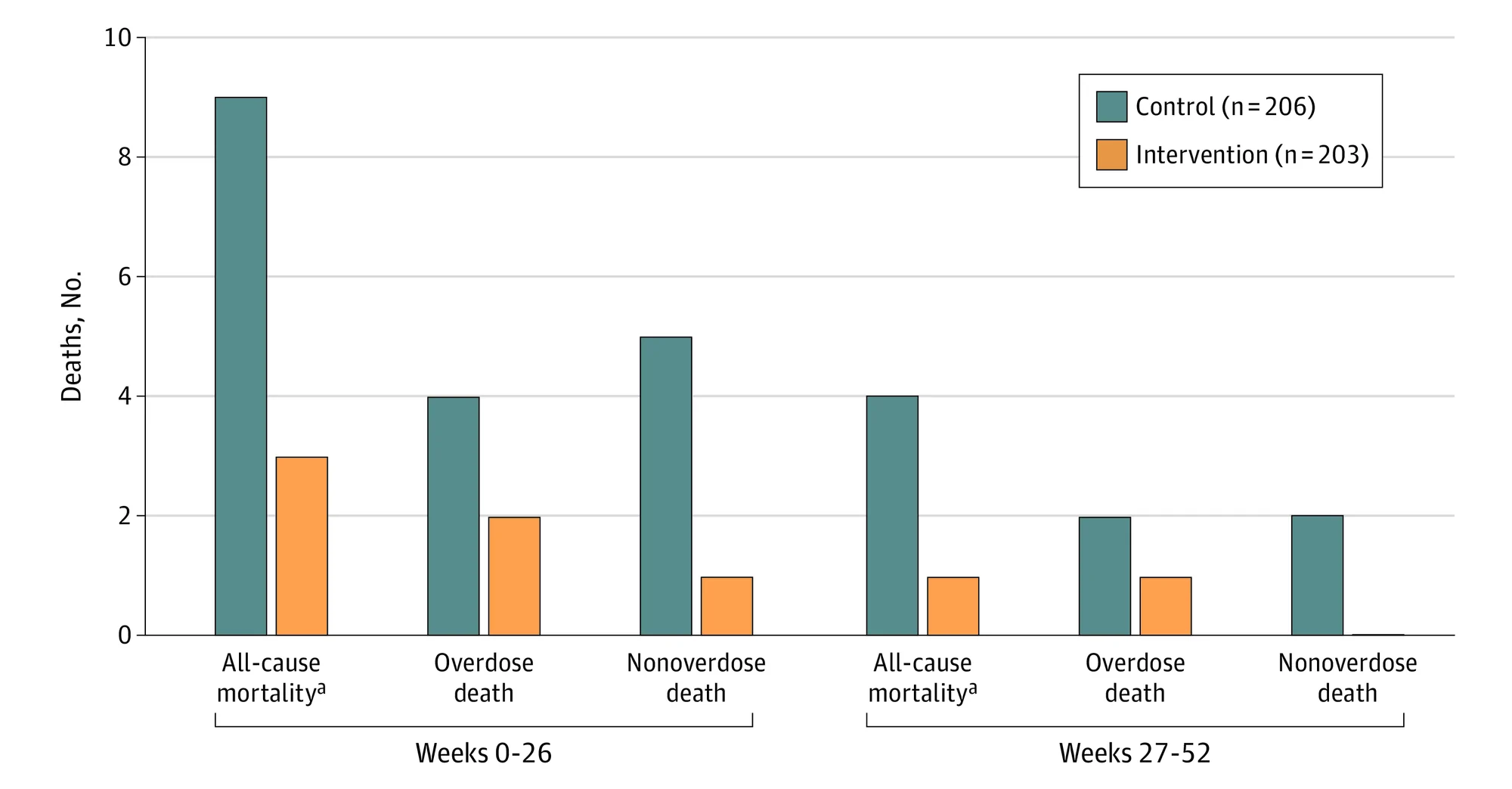
Bar Charts Showing Numbers of All-Cause, Overdose, and Nonoverdose Deaths by Assignment to Study Arm for the Periods From 0 to 26 Weeks and 27 to 52 Weeks ^a^The number of all-cause mortality deaths in each period is the sum of overdose and nonoverdose deaths by study arm for each 26-week period.

## Discussion

Although delivery of services on the mobile unit was hypothesized to reduce access barriers and improve uptake of integrated PrEP and MOUD services, the intervention did not significantly improve either co–primary outcome compared with manualized peer navigation to available community services at week 26 or at week 52. Verified PrEP use was low at 26 weeks for both study arms, even when provided consistent peer navigation services, with lower percentages observed at 52 weeks. Nevertheless, results are favorable compared to self-reported prevalence of 1% of PrEP use among people who inject drugs.^18^ Consistent with national estimates of HIV incidence among people who inject drugs of about 1 per 100 person-years,^1^ the HIV incidence rate at 52 weeks observed was approximately 1.0 per 100 person-years, justifying recommendations for HIV PrEP use for people who inject drugs. While findings demonstrate a suboptimal use of oral daily PrEP for people who inject drugs, even when provided with peer navigation encouraging its use, the incidence rate observed elevates the importance of future studies of long-acting PrEP formulations with people who inject drugs.

The integrated strategies, peer navigation, and mobile units in HPTN 094 showed some signal for retaining participants and for supporting MOUD use from enrollment visits to weeks 26 and 52. Although not statistically different between study arms, findings of about 14% MOUD use at both 26- and 52-week visits were clinically meaningful, as no participants were taking MOUD at study entry. Findings showing a minority of participants taking MOUD at both 26- and 52-week visits indicated that once enrolled in the study, people who inject drugs started taking MOUD, discontinued taking MOUD, and, for some, recommenced taking MOUD over the course of a year. Exploratory and future research will consider patterns of entry and withdrawal from MOUD through the study period, with particular attention to factors such as incarceration and housing instability.

The significant reduction in all-cause mortality observed by 52 weeks for the intervention arm as a secondary outcome is a key finding of this trial, though preliminary and needing replication. Although the number of deaths by arm was small, the effect size was large, with a 71% lower risk of death in the intervention arm compared to the active control arm at 52 weeks. This finding is consistent with a prior report showing integrated service models may meaningfully influence mortality risk among people who inject drugs by altering both access to and continuity of care.^16^ It may be that clinicians in the mobile units were more accessible to participants than clinicians at brick-and-mortar facilities, which may correspond with more timely intervention or more healthful participant behavior in the mobile units. The use of mobile units may provide additional benefit by delivering low-threshold services in geographically and socially accessible settings where people who inject drugs live, a rationale that merits future study.

### Strengths and Limitations

Strengths of this study include a randomized design across diverse urban contexts, high retention in a highly mobile and marginalized population, and the use of objective biomarker measures for both PrEP and MOUD. Women and persons from various race and ethnicity groups were well represented, addressing a gap in prior research.^7^ Because both arms received manualized peer navigation, the study was designed to estimate the marginal effect of mobile integrated service delivery beyond navigation, which may have reduced between-arm differences, but strengthened the specificity of the comparison. Fidelity-supporting procedures were used during implementation of the peer navigation intervention.

This trial also has limitations. The study was powered to detect differences in composite use of PrEP and MOUD, not HIV incidence or mortality. No process data describing delivery of integrated services were available to test links between intervention delivery and study outcomes for either study arm. Methadone was not available on the mobile unit, which may have functioned more like facilitated referral than full mobile treatment delivery. The trial occurred during and after periods of COVID-19-related service disruptions and changing community naloxone distribution, contextual factors that may have affected navigation modality, access to brick-and-mortar services, overdose response, and mortality risk. Heterogeneity across sites that included differences in access to health care, including MOUD, may have influenced effect estimates.

## Conclusions

In this randomized clinical trial, there were no differences measured between the intervention and control arms regarding HIV PrEP and MOUD use. Nonetheless, delivering integrated HIV and addiction services using mobile units appeared to reduce all-cause mortality rates among people who inject drugs over and above peer navigation to these services alone. Mobile units may confer benefits additional to peer navigation by delivering low-threshold services in geographically and socially accessible settings, thereby reaching individuals at highest risk. Together, findings showed peer-navigation services and use of mobile units align with implementation science frameworks that emphasize reach, timeliness, and service penetration as key determinants of population-level impact for people who inject drugs in the US.

## Notes

Supplement 1. Protocol and statistical analysis plan

Supplement 2. eTable 1. Overview of Medical Care Provided in the Mobile Unit for Intervention Arm Participants eAppendix 1. Description of limited medical care, HIV testing, PrEP provision, PrEP adherence assessments, and MOUD and other drug use eAppendix 2. Links to Peer Navigation Manual and the Graphical Peer Navigation Manual eTable 2. Cumulative all−cause mortality through week 26 and 52, overall and by site

Supplement 3. Data sharing statement
