# Supplementary material for: Mobile Units for Integrated Addiction and HIV Prevention Services for People Who Inject Drugs: The HPTN 094 Randomized Clinical Trial
Source: JAMA Psychiatry. 2026 Nov 4:e263644. Online ahead of print. doi: 10.1001/jamapsychiatry.2026.3644 (PMC13735193; doi:10.1001/jamapsychiatry.2026.3644)
Supplement: Supplementary file 1 [file jamapsychiatry-e263644-s001.pdf]

HPTN 094

INTEGRA: A Vanguard Study of Health Service Delivery in a Mobile Health Delivery Unit to Link Persons who Inject Drugs to Integrated Care and Prevention for Addiction, HIV, HCV and Primary Care

DAIDS Document ID: 38715

A Study by the HIV Prevention Trials Network (HPTN)

Sponsored by:
Division of AIDS (DAIDS), United States (US) National Institute of Allergy and Infectious Diseases (NIAID)
US National Institutes of Health (NIH)

Funded by:
US National Institute on Drug Abuse

Protocol Chair:
Steve Shoptaw, PhD

Protocol Co-Chair:
Nabila El-Bassel, PhD

Final Version 1.0
15 July 2020

HPTN 094
INTEGRA: A Vanguard Study of Health Service Delivery in a Mobile Health Delivery Unit to Link Persons who Inject Drugs to Integrated Care and Prevention for Addiction, HIV, HCV and Primary Care

TABLE OF CONTENTS

PROTOCOL SIGNATURE PAGE **5**
LIST OF ABBREVIATIONS AND ACRONYMS **6**
PROTOCOL TEAM ROSTER **8**
SCHEMA **11**
1. INTRODUCTION **16**
1.1 Background and Rationale 16
1.2 Rationale for Study Design 17
1.2.1 Implementation Science Framework 20
2.0 STUDY OBJECTIVES **21**
2.1 Primary Objective 21
2.2 Secondary Objectives 21
2.3 Process Objectives 22
2.4 Exploratory Objectives 22
3.0 STUDY DESIGN **22**
3.1 Participating Sites/Institutions 23
3.2 Study Duration 23
4.0 STUDY POPULATION **24**
4.1 Inclusion Criteria 24
4.2 Exclusion Criteria 24
4.3 Recruitment Process 25
4.4 Co-Enrollment Guidelines 26
4.5 Participant Retention 26
4.6 Participant Withdrawal from Study 27
5.0 STUDY INTERVENTIONS **27**
5.1 Initial Assessment & Provision of Services in the Mobile Unit - All Participants 27
5.2 Provision of Medical Services 28
5.2.1 Medical Services - Intervention Arm 28
5.2.2 Medical Services - Active Control Arm 29
5.3 Provision of Peer Navigation Services 29
5.3.1 Navigation Services - Intervention Arm 30

5.3.2 Navigation Services- Active Control Arm .... 31~~30~~

5.4 Drug Supply 31

6.0 STUDY PROCEDURES 38

6.1 Screening Visit 38

6.2 Enrollment Visit 39

6.3 Clinical Care and Navigation Visits Between Enrollment and Week 26 39

6.4 Week 26 Visit 40

6.5 Week 52 Visit 41

6.6 Implementation Evaluation & Cost-Effectiveness Data Collection Procedures . 41

7.0 SAFETY MONITORING AND SERIOUS ADVERSE EVENT REPORTING 44

7.1 Adverse Event Definition and Reporting 44

7.2 Grading Severity of Events 45

7.3 Reporting Timeframe 45

7.4 Social Impact Reporting 45

7.5 Other Unanticipated Problems 46

7.6 Safety Monitoring and Clinical Data Review 46

7.7 Pregnancy 47

8.0 STATISTICAL CONSIDERATIONS .... 48~~47~~

8.1 Review of Study Design .... 48~~47~~

8.2 Endpoints .... 48~~47~~

8.2.1 Primary Endpoints .... 48~~47~~

8.2.2 Secondary Endpoints 48

8.2.3 Process Endpoints .... 52~~51~~

8.3 Sample Size .... 53~~52~~

8.4 Accrual and Retention .... 53~~52~~

8.5 Random Assignment/Study Arm Assignment .... 53~~52~~

8.6 Blinding .... 53~~52~~

8.7 Data and Safety Monitoring Oversight .... 53~~52~~

8.8 Statistical Analysis .... 54~~53~~

8.8.1 Primary Analyses .... 54~~53~~

8.8.2 Secondary Analyses .... 54~~53~~

9.0 HUMAN SUBJECTS CONSIDERATIONS .... 55~~54~~

9.1 Ethical Review .... 55~~54~~

9.2 Informed Consent .... 55~~54~~

9.3 Risks .... 56~~55~~

9.4 Benefits .... 56~~55~~

9.5 Population-Specific Considerations .... 57~~56~~

9.6 Incentives .... 58~~57~~

9.7 Confidentiality .... 58~~57~~

9.8 Communicable Disease Reporting Requirements .... 58~~57~~

9.9 Study Discontinuation .... 59~~58~~

10.0 LABORATORY SPECIMENS AND BIOHAZARD CONTAINMENT .... 59~~58~~

10.1 Local Laboratory Specimens .... 59~~58~~

10.2 HPTN LABORATORY CENTER SPECIMENS .... 60~~59~~
10.2.1 Stored Specimens .... 60~~59~~
10.2.2 Virology .... 60~~59~~
10.2.3 Pharmacology and Toxicology Testing .... 61~~60~~
10.3 QUALITY CONTROL AND QUALITY ASSURANCE PROCEDURES .... 61~~60~~
10.4 QC FOR HIV DIAGNOSTIC TESTING .... 62~~61~~
10.5 HIV RNA MONITORING .... 62~~61~~
10.6 CD4 CELL COUNT DETERMINATION .... 62~~61~~
10.7 SPECIMEN STORAGE AND POSSIBLE FUTURE RESEARCH TESTING .... 62~~61~~
10.8 BIOHAZARD CONTAINMENT .... 62~~61~~

11.0 ADMINISTRATIVE PROCEDURES .... 63~~62~~
11.1 PROTOCOL REGISTRATION .... 63~~62~~
11.2 STUDY ACTIVATION .... 63~~62~~
11.3 STUDY COORDINATION .... 63~~62~~
11.4 STUDY MONITORING .... 64~~63~~
11.5 PROTOCOL COMPLIANCE .... 64~~63~~
11.6 INVESTIGATOR'S RECORDS .... 64~~63~~
11.7 USE OF INFORMATION AND PUBLICATIONS .... 65~~64~~

REFERENCES .... 66~~65~~

APPENDIX IA: SCHEDULE OF STUDY VISITS AND PROCEDURES FOR PARTICIPANTS WHO ARE HIV POSITIVE AT ENROLLMENT .... 69~~68~~

APPENDIX IB: SCHEDULE OF STUDY VISITS AND PROCEDURES FOR PARTICIPANTS WHO ARE HIV NEGATIVE AT ENROLLMENT .... 73~~72~~

APPENDIX IC: ADDITIONAL PROCEDURES FOR PARTICIPANTS WHO HAVE A REACTIVE OR POSITIVE HIV TEST AFTER ENROLLMENT .... 76~~75~~

APPENDIX II: SAMPLE INFORMED CONSENT FORM- MAIN STUDY .... 77~~76~~

APPENDIX III: SAMPLE INFORMED CONSENT FORM- QUALITATIVE INTERVIEWS . 92~~91~~

HPTN 094

INTEGRA: A Vanguard Study of Health Service Delivery in a Mobile Health Delivery Unit to Link Persons who Inject Drugs to Integrated Care and Prevention for Addiction, HIV, HCV and Primary Care

PROTOCOL SIGNATURE PAGE

DAIDS Document ID # 38715

Final Version 1.0
15 July 2020

I will conduct the study in accordance with the provisions of this protocol and all applicable protocol-related documents. I agree to conduct this study in compliance with United States (US) Health and Human Service regulations (45 CFR 46); applicable US Food and Drug Administration regulations; standards of the International Conference on Harmonization Guideline for Good Clinical Practice (E6); Institutional Review Board/Ethics Committee determinations; all applicable in-country, state, and local laws and regulations; and other applicable requirements (e.g., US National Institutes of Health, Division of AIDS) and institutional policies.

I have read and understand the information in this protocol and will ensure that all associates, colleagues, and employees assisting in the conduct of the study are informed about the obligations incurred by their contribution to the study.

____
Name of Investigator of Record (print name)

_________________________________ ____________________
Signature of Investigator of Record Date (MM/DD/YYYY)

HPTN 094

INTEGRA: A Vanguard Study of Health Service Delivery in a Mobile Health Delivery Unit to Link Persons who Inject Drugs to Integrated Care and Prevention for Addiction, HIV, HCV and Primary Care

LIST OF ABBREVIATIONS AND ACRONYMS

Ab	Antibody
AE	Adverse Event
AIDS	Acquired immunodeficiency syndrome
ALT	Alanine Aminotransferase
aPTT	Activated Partial Thromboplastin Time
ART	Antiretroviral therapy
ARV	Antiretroviral
AST	Aspartate Aminotransferase
CAB	Community Advisory Board
CDC	(United States) Centers for Disease Control and Prevention
CEA	Cost-effectiveness analyses
CFR	Code of Federal Regulations
CLIA	Clinical Laboratory Improvement Amendments of 1988
CMC	Clinical Management Committee
COWS	Clinical Opiate Withdrawal Scale
COVID-19	Coronavirus Disease 2019
CPQA	Clinical Pharmacology Quality Assurance
CRM	Clinical Research Manager
CRS	Clinical Research Site
CT	*Chlamydia trachomatis*
DAIDS	Division of AIDS
DBS	Dried Blood Spot
DHHS	US Department of Health and Human Services
DSM	Diagnostic and Statistical Manual of Mental Disorders
DSMB	Data and Safety Monitoring Board
FDA	(United States) Food and Drug Administration
FQHC	Federally-Qualified Health Centers
GC	*Neisseria gonorrhoeae*
GCLP	Good Clinical Laboratory Practice
HAV	Hepatitis A Virus
HBV	Hepatitis B Virus
HCG	Human Chorionic Gonadotropin
HCV	Hepatitis C Virus
HIV	Human Immunodeficiency Virus
HLA	Human Leukocyte Antigen
HPTN	HIV Prevention Trials Network
ICER	Incremental Cost Effectiveness Ratio
ICF	Informed consent form
IgG	Immunoglobulin G
IoR	Investigator of Record
IRB	Institutional Review Board
LC	(HPTN) Laboratory Center
LDMS	Laboratory Data Management System

LOC	Leadership and Operations Center
mL	Milliliter
MOP	Manual of Operations
MOUD	Medication for Opioid Use Disorder
MSM	Men who have Sex with Men
NAAT	Nucleic Acid Amplification Test
NAESM	National AIDS Education & Services for Minorities
NHBS	National HIV Behavioral Surveillance
NIAID	(United States) National Institute of Allergy and Infectious Diseases
NIDA	National Institute on Drug Abuse
NIH	(United States) National Institutes of Health
OHRP	Office of Human Research Protections
OUD	Opioid Use Disorder
PrEP	Pre-Exposure Prophylaxis
PRISM	Practical, Robust Implementation and Sustainability Model
PRO	(DAIDS) Protocol Registration Office
PT	Prothrombin Time
PWID	People Who Inject Drugs
QA	Quality Assurance
QALY	Quality-Adjusted Life Year
QC	Quality Control
RE-AIM	Reach, Effectiveness, Adoption, Implementation, Maintenance
RNA	Ribonucleic acid
RSC	Regulatory Support Center
SAE	Serious Adverse Event
SARS-CoV-2	Severe Acute Respiratory Syndrome Coronavirus 2
SDMC	(HPTN) Statistical and Data Management Center
SEP	Syringe exchange program
SMC	Study Monitoring Committee
SNS	Social Network Strategy
STI	Sexually transmitted infection
SOC	Standard of Care
SOP	Standard Operating Procedures
SSP	Study Specific Procedures
US	United States
VL	Viral load

HPTN 094

INTEGRA: A Vanguard Study of Health Service Delivery in a Mobile Health Delivery Unit to Link Persons who Inject Drugs to Integrated Care and Prevention for Addiction, HIV, HCV and Primary Care

PROTOCOL TEAM ROSTER

PROTOCOL CHAIR:
Steve Shoptaw, PhD
University of California Los Angeles
Departments of Family Medicine and Psychiatry and Biobehavioral Sciences
10880 Wilshire Blvd, Suite 1800
Los Angeles, CA 90024
Phone: 310-740-7021
Email: sshoptaw@mednet.ucla.edu

PROTOCOL CO-CHAIR
Nabila El-Bassel, PhD
Columbia University
Professor, School of Social Work
Director, Global Health Research Center of Central Asia
Director, Social Intervention Group (SIG)
1255 Amsterdam Avenue
New York, NY 10025
Phone: 212.851-2391
Email: ne5@columbia.edu

Philip Andrew, RN
Senior Research Clinician
HPTN Leadership and Operations Center
FHI 360
359 Blackwell Street, Suite 200
Durham, NC 27701
Phone: 919-544-7040 Ext. 11213
Email: pandrew@fhi360.org

David Burns, MD, MPH
Chief, Clinical Prevention Research Branch
Prevention Sciences Program, Division of AIDS, NIAID
5601 Fishers Lane, Rm 8B40
Rockville, MD 20852
Phone: 301.435.8896
Email: burnsda@niaid.nih.gov

Dale Burwen, MD, MPH
Medical Officer
Clinical Prevention Research Branch
Prevention Sciences Program, Division of AIDS, NIAID
5601 Fishers Lane, Rm 8B49
Rockville, MD 20852
Phone: 240.669.2867
Email: dale.burwen@nih.gov

Redonna K. Chandler, Ph.D.
Director, AIDS Research Program
Director, HEALing Communities Study
National Institute on Drug Abuse
6001 Executive Boulevard, Room 5265
Rockville, MD 20892-9581
Phone: 301-443-1470
E-mail: redonna.chandler@nih.gov

William Clarke, PhD, DABCC
Professor of Pathology
HPTN LC Toxicology Core Director
Johns Hopkins University School of Medicine
1551 E. Jefferson Street/CRB II 3M03
Baltimore, MD 21287
Phone: 410-502-7692
Email: wclarke@jhmi.edu

Melissa Cummings, MPH
SCHARP Clinical Data Manager
Fred Hutch
1100 Fairview Ave N, E3-129
Seattle, WA 98109
Phone: 206-667-1232
Email: mcumming@fredhutch.org

Dobromir Dimitrov, PhD
Mathematical Modeler
HPTN Modeling Center
Fred Hutch
1100 Fairview Ave N
Seattle, WA 98109
Phone: 206-667-1933
Email: ddimitro@fredhutch.org

Deborah Donnell, PhD
Biostatistician
HPTN Statistics and Data Management Center
Fred Hutch
1100 Fairview Ave N
Seattle, WA 98109
Phone: 206-667-5661
Email: deborah@fredhutch.org

Jessica M. Fogel, PhD
HPTN Laboratory Center Representative
Johns Hopkins University School of Medicine
720 Rutland Ave., Ross Bldg Rm 646
Baltimore, MD 21205
Phone: 410-614-6498
Email: jfogel@jhmi.edu

David Goodman-Meza, MD, MAS
Assistant Professor
UCLA, Division of Infectious Diseases
10833 Le Conte Ave (Room 37-121CHS)
Los Angeles, CA, 90095-1688
Phone: 310-825-8373
Email: dgoodman@mednet.ucla.edu

Sam Griffith, MAT
Senior Clinical Research Manager
HPTN Leadership and Operations Center
FHI 360
359 Blackwell Street, Suite 200
Durham, NC 27701
Phone: 919-544-7040 Ext. 11571
Email: sgriffith@fhi360.org

Brett Hanscom, PhD
Biostatistician
HPTN Statistics and Data Management Center
Fred Hutch
1100 Fairview Ave N
Seattle, WA 98109
Phone: 206-667-4534
Email: bhanscom@fredhutch.org

Abraham Johnson, MPH
Community Program Associate
HPTN Leadership and Operations Center
FHI 360
359 Blackwell Street, Suite 200
Durham, NC 27701
Phone: 919-544-7040 Ext. 11882
Email: ajohnson@fhi360.org

Irene Kuo, PhD, MPH
Associate Research Professor
George Washington University
Milken Institute School of Public Health
950 New Hampshire Avenue NW, Suite 500
Washington, DC 20052
Phone: 202-994-0367
Email: ikuo@gwu.edu

Timothy Skalland, PhD
Biostatistician
HPTN Statistics and Data Management Center
Fred Hutch
1100 Fairview Ave N
Seattle, WA 98109
Phone: 206-667-1186
Email: tskallan@fredhutch.org

Jonathan Paul Lucas, MPH
Senior Community Program Manager
HPTN Leadership and Operations Center
FHI 360
359 Blackwell Street, Suite 200
Durham, NC 27701
Phone: 919-544-7040 Ext. 11458
Email: jlucas@fhi360.org

James Moore, PhD
Biomathematician
HPTN Modeling Center
Fred Hutch
1100 Fairview Ave N
Phone: 206-667-2252
Email: jrmoore@fredhutch.org

Paul Richardson, MSc
Senior International QA/QC Coordinator
HPTN Laboratory Center
Johns Hopkins University
Pathology 306
600 N. Wolfe St.
Phone: 410-502-0435
Email: pricha18@jhmi.edu

Laramie R. Smith, PhD
Assistant Professor
UC San Diego, School of Medicine
9500 Gillman Dr. #0507
La Jolla, CA 9209.-0507
Phone: 858-822-1462
Email: laramie@health.ucsd.edu

Chloe Thio, MD
Professor of Medicine
HPTN Laboratory Center
Johns Hopkins University School of Medicine
855 N Wolfe Street, 5th floor
Baltimore, MD 21205
Phone: 410-614-6088
Email: cthio@jhmi.edu

HPTN 094

INTEGRA: A Vanguard Study of Health Service Delivery in a Mobile Health Delivery Unit to Link Persons who Inject Drugs to Integrated Care and Prevention for Addiction, HIV, HCV and Primary Care

SCHEMA

Purpose: The purpose of this study is to determine the efficacy of using a mobile health delivery unit ("mobile unit") to deliver "one stop" integrated health services – particularly medication for opioid use disorder (MOUD) and medication for HIV treatment and prevention – to people who inject drugs (PWID) with opioid use disorder (OUD) to improve uptake and use of MOUD, and uptake and use of antiretroviral therapy (ART) or pre-exposure prophylaxis (PrEP). The intervention arm receiving health services in the mobile unit will be supported by peer navigation. An active control arm will receive peer navigation to health services available at community-based agencies. Impact (cost-effectiveness, mathematical modeling) and implementation factors (mixed methods to identify barriers and facilitators of the interventions) will contextualize findings from the efficacy analysis. The impact of the COVID-19 epidemic in the study population will also be assessed.

Design: A two-arm, individually randomized, controlled, open label study

Population: HIV-positive and HIV-negative PWID with OUD not receiving MOUD

Study Size: A total of 860 participants allotted in a 1:1 ratio to intervention and active control arms, with targets of a minimum 25% women and 25% participants under 30 years of age, with approximately 460 HIV-positive and 400 HIV-negative participants.

Study Duration: Approximately three and a half years total with individual participants on study for approximately 52 weeks (26 weeks receiving the intervention or peer navigation and then an evaluation for durability of effect at the end of 52 weeks).

Study Sites: Five urban sites in the United States (US) with substantial populations of PWID with OUD. See the Study Specific Procedures (SSP) Manual for site listing.

Study Regimen: All potential study participants will provide biological samples and self-reported data via interview in the mobile unit at Screening and Enrollment Visits. Samples will be tested for HIV, hepatitis A (HAV), B (HBV) and C (HCV) and sexually transmitted infections (STIs). At the Enrollment Visit, participants who meet all inclusion and no exclusion criteria will be randomized to the intervention or active control arm and will receive harm reduction services and empiric treatment for STIs if symptomatic. Participants will also be assessed for COVID-19 at study visits; participants

with suspected COVID-19 will be referred for further evaluation, care and/or treatment, as available.

Intervention Arm: Participants in the intervention arm will be provided integrated health services delivered in the mobile unit and peer navigation for 26 weeks. The integrated health services in the mobile unit will include:

- MOUD and harm reduction services for OUD
- HIV testing
- HIV treatment for HIV-positive participants not already in care
- PrEP for HIV-negative participants
- Testing and referral for vaccination or treatment for HAV and HBV
- Testing and referral for treatment for HCV
- STI testing and treatment
- Primary care
- Harm reduction services

Peer navigation in the intervention arm will coordinate and facilitate integrated care in the mobile unit through 26 weeks. As participants become established in care, navigation will help transition participants to community-based services by 26 weeks after randomization.

Active Control Arm: Participants in the active control arm will be provided 26 weeks of peer navigation to connect them to health services available at community-based agencies.

All participants (both arms) will have study visits at 26 and 52 weeks for evaluation of study endpoints.

Primary Objective: To evaluate whether 26 weeks of "one stop" integrated health services delivered in a mobile unit, supported by peer navigation, improves use of MOUD, increases rates of viral suppression among HIV-positive individuals, and increases use of PrEP among HIV-negative individuals, as measured at 26 weeks, when compared to 26 weeks of peer navigation to similar health services available at community-based agencies.

Secondary Objectives:

1) To evaluate whether 26 weeks of "one stop" integrated health services delivered in a mobile unit, supported by peer navigation, compared to 26 weeks of peer navigation to similar health services available at community-based agencies:
    a) improves use of MOUD at 52 weeks
    b) increases rates of viral suppression among HIV-positive individuals at 52 weeks
    c) increases use of PrEP among HIV-negative individuals at 26 and 52 weeks
    d) decreases opioid and polysubstance use at 26 and 52 weeks
    e) decreases prevalence of bacterial STIs at 26 and 52 weeks
    f) decreases fatal and non-fatal overdose events by 26 and 52 weeks
    g) increases the proportion of participants with undetectable HCV RNA at 26 and 52 weeks among those with chronic HCV infection at enrollment

h) decreases HCV incidence at 52 weeks, for those who are HCV negative at enrollment

2) To evaluate whether 26 weeks of "one stop" integrated health services delivered in a mobile unit, supported by peer navigation, increases MOUD use, viral suppression, and PrEP use at 26 and 52 weeks compared to Enrollment.

3) To evaluate whether 26 weeks of peer navigation to similar health services available at community-based agencies increases MOUD use, viral suppression, and PrEP use at 26 and 52 weeks compared to Enrollment

4) To document the prevalence of seropositivity for SARS-CoV-2 at baseline, 26 and 52 weeks

5) To document the impact of the COVID-19 epidemic on participants' experiences of seeking, obtaining and/or maintaining health services, housing, food security and drugs

6) To evaluate implementation of "one-stop" integrated health services using a mobile unit, supported by peer navigation, across study sites to identify mechanisms at multiple levels to:
   a) Guide real-time improvements and refinements in the conduct of the study to ensure primary and secondary outcomes are met with fidelity
   b) Examine the quality and process of services delivered in each study arm, particularly as these affect primary and secondary outcomes
   c) Develop evidence-based guidance for policymakers on the uptake and implementation of integrated health services using peer navigation and mobile health units in urban US regions to address HIV in PWID
   d) Identify factors that enhance or impede the delivery of integrated health services using a mobile unit, supported by peer navigation, on primary and secondary outcomes, including responding to the impact of COVID-19 on service delivery

7) To use mathematical modeling methods to:
   a) Estimate the effect of integrated health services delivered in a mobile unit, supported by peer navigation, on reducing HIV incidence in PWID and their sexual and injection partners
   b) Estimate the cost-effectiveness of the integrated health services provided in a mobile unit and supported by peer navigation

Process Objectives:

For participants in the intervention arm, assess:

1) Time to provide MOUD treatment, ART (among those who are HIV positive and not on ART at Enrollment) and PrEP (among those who are HIV negative at Enrollment)
2) The proportion of participants linked to community-based MOUD, ART and PrEP services at 26 weeks

Exploratory Objectives:

Stored samples will be used to analyze HIV subtypes/strains, HIV drug resistance, and the duration of HIV infection. Phylogenetic methods will be used to evaluate behavioral, demographic, and clinical factors associated with viral clusters and transmission dynamics. Stored samples may also be used characterize HCV strains and the relationship between HIV and HCV infections, and to explore issues related to COVID-19.

Figure 1- Overview of Study Design

Formatted: Numbering: Continuous

HPTN 094

INTEGRA: A Vanguard Study of Health Service Delivery in a Mobile Health Delivery Unit to Link Persons who Inject Drugs to Integrated Care and Prevention for Addiction, HIV, HCV and Primary Care

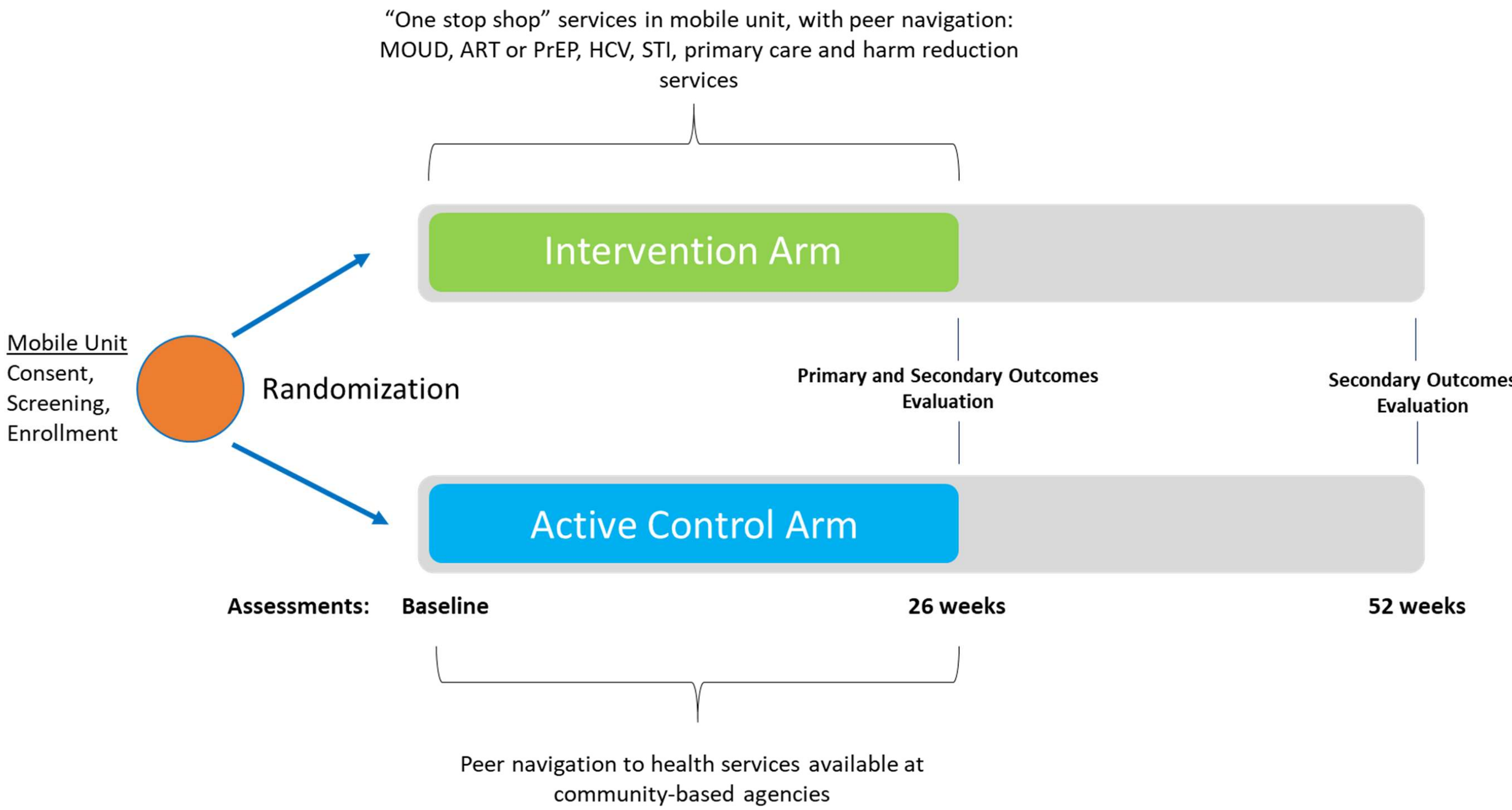

1. INTRODUCTION

Formatted: Numbering: Continuous

1.1 Background and Rationale

Drug overdose is the leading cause of accidental death in the United States (US), with over 67,000 fatalities in 2018.[1] Efforts to address the opioid overdose epidemic are reducing rates of death due to prescription opioids, though the rate of overdose related to fentanyl use continued to rise through 2018.[1] The opioid epidemic remains North America's most widespread behavioral public health problem, with a higher number of deaths due to drug overdose in 2016 compared to deaths due to HIV at the peak of the AIDS epidemic in the US.[2] The growing epidemic now significantly involves Black and Latino individuals in urban areas such as New York City, where opioid overdose rates among Blacks surpassed Whites for the first time in 2019.[3-5] Progression from prescription opioid use to injection heroin use is common, though the increase has slowed.[6-8] Fentanyl and its analogues represent the "third wave" of the US opioid epidemic and are major contributors to the ongoing and massive increase in drug overdose deaths.[1,7] Injection drug use is the primary driver for doubling hepatitis C (HCV) incidence in the US, with several recent HCV outbreaks among people who inject drugs (PWID).[9-13] Moreover, unsafe injection practices that drive HCV incidence often foreshadow increases in HIV incidence, and most PWID living with HIV are co-infected with HCV.[14] The latest surveillance reports continue to show increases in methamphetamine and cocaine involvement in drug overdose deaths.[1]

Health risks for persons with OUD and living with or at risk for HIV in the US include multiple and overlapping problems that interfere with consistent access to health care, particularly when the substance use disorder is active.[15] The behaviorally disorganizing aspects of substance use disorder can interfere with traveling to existing services (lack of transportation), with willingness to tolerate judgements and attitudes from personnel in health care settings (stigma) and with foregoing pleasure in lieu of healthcare (addiction). Consequently, persons with active addiction spend large amounts of time hanging out together in "hot spots" near jails, criminal justice community supervision programs, syringe exchange programs (SEPs), parks and tourist areas. The location and size of these hot spots can vary within regions.

This calls for an HIV prevention response that meets people living with or at-risk for HIV who also live with OUD where they are, with the aim to reduce barriers at each step in the process of accessing and sustaining medication for opioid use disorder (MOUD) treatment and HIV care and prevention. Moreover, there is a need to examine barriers that interfere with entry and retention in MOUD and with sustaining relevant HIV outcomes. This is particularly true among this population, many of whom face additional stigma, barriers and health disparities related to their multi-dimensional identities (e.g., as sex workers, men who have sex with men (MSM) and polysubstance users).[15]

The rationale for this study is based on this premise: HIV outbreaks in the US among PWID occur when simultaneous factors—lack of access to health care (including MOUD), poverty, prevalent poly-substance use, and mental health disorders and others—combine to exacerbate HIV transmission and acquisition. PWID living with or at risk of HIV who are not engaged in MOUD face the nearly impossible task of getting care from bricks and mortar clinics that provide separate, siloed care for opioid addiction (methadone, buprenorphine), HIV (ART and PrEP) and primary care (STI testing and treatment, hepatitis testing and treatment, diagnosis and treatment for chronic and other conditions) with limited or no financial resources. Health services to address these diverse problems, when they exist, are often located far away from each other and from the PWID who need them, presenting serious distance/travel barriers to access. The

challenge of finding and sustaining HIV care and prevention in the setting of untreated OUD in persons with multiple additional health threats contributed to multiple HIV outbreaks in the US (in Indiana, Massachusetts, Washington, West Virginia).[16-18] The use of a mobile venue that meets out-of-treatment PWID wherever they might be in their communities and links them to care systems and/or harm reduction is innovative, is likely to save lives and, if efficacious, could be efficiently scaled-up in the US, especially as the US health system responds to dramatic new pressures imposed by the COVID-19 pandemic.

The initial COVID-19 outbreak in early 2020 changed the world in innumerable ways, including general disruption of daily life, implementation of social distancing policies, and prioritization of healthcare systems to address the coronavirus. These changes exacerbated challenges PWID already faced to initiating or maintaining treatment/prophylaxis regimens for OUD and HIV and to obtaining other health care. Creative new strategies by the healthcare system in response to the COVID-19 epidemic included use of medical consultations by telephone or video (telehealth), and relaxing restrictions on how medications could be prescribed and dispensed (particularly buprenorphine/naloxone); these strategies have the potential to help PWID receive healthcare and maintain adherence. It is not clear what the healthcare environment will look like at the time this study begins, or how it will change during study implementation. To that end, this protocol includes research objectives that address the effect of COVID-19 on the lives and healthcare of PWID. We describe adaptations that we anticipate will be necessary to implement this study and this intervention successfully in the context of an ongoing or resurgent COVID-19 epidemic. We will also consider incorporating additional healthcare delivery activities during the study, such as COVID-19 diagnostic testing, based on feasibility, availability, and other factors.

1.2 Rationale for Study Design

This vanguard study addresses the current and overlapping health challenges to engaging and retaining HIV-positive or at-risk persons in HIV care and prevention at a time when OUD has re-emerged as a potential driver for HIV infections in the US. It does this by integrating engagement and retention for HIV care and prevention with the parallel effort to engage and retain PWID with OUD in treatment using MOUD[19,20] (i.e., buprenorphine, methadone and extended-release naltrexone). For persons with OUD, MOUD functions as a key harm reduction and treatment strategy by reducing the disruptive and dysregulating behaviors of addiction and by lowering the likelihood of overdose and death. In this way, MOUD enables increased uptake and adherence to antiretroviral therapy (ART) among people living with both HIV and OUD [21,22] including PWID. To date, however, only 15% of those with OUD in the US are receiving MOUD.[9,23] This vanguard study will test the efficacy and the impact of an integrated strategy that provides integrated care services including provision of ART and pre-exposure prophylaxis (PrEP), MOUD treatment, STI testing and treatment, primary health care, and harm reduction from a mobile health delivery unit ("mobile unit") and supported by peer navigation compared with an active control arm that receives peer navigation to similar health services available at community-based agencies. Primary outcomes are centered on HIV and OUD: viral suppression, PrEP use, and uptake and use of MOUD. This study will also offer an unparalleled opportunity to document the prevalence of SARS-CoV-2 seropositivity in this population at baseline and over time, and to investigate the impact of the COVID-19 epidemic on the lives of people with opioid addiction and living with or at risk for HIV.[24,25]

Fit with the Network. The INTEGRA integrated strategy accomplishes two important objectives of the HIV Prevention Trials Network (HPTN): (1) To conduct HIV research in at-risk opioid injectors and measure linked MOUD and HIV outcomes in the US; and (2) to address the multiplicity of challenges and barriers to primary care, HIV and HCV prevention and care, and

MOUD/harm reduction programs. This study is well suited to make use of the resources of the HPTN to conduct high impact HIV prevention work in opioid injectors, a key priority population for the HPTN. The availability of research sites in diverse geographic locations and the statistical and laboratory capacities of the HPTN are other key advantages of network engagement. The INTEGRA integrated strategy also addresses in real time and in full measure the call for integrated strategies to respond to the ongoing dual epidemics of HIV and opioid use disorder,[26] and to respond to a novel third epidemic, COVID-19.

Rationale for use of Mobile Health Delivery Units. The literature on mobile health service provision includes low to moderate quality studies showing that mobile units improve care outcomes in multiple settings and for multiple health needs. Needle exchange programs delivered from mobile units have been successful in engaging PWID in services that are either unacceptable or unavailable in brick and mortar settings.[27,28] These prior studies provide key support for the design of this study. This could raise the criticism that the outcome of this trial can be assumed to be favorable and does not require a randomized investigation. While there is hope and expectation the intervention will be efficacious, there are numerous reasons why this study is worthwhile even if one were to assume a positive outcome:

- First, these would be the first data to inform the improvement in outcomes using a mobile health delivery system, above an energetic navigation condition, for improving rates of viral suppression and PrEP adherence among PWID and for engaging and retaining these individuals in MOUD for significant periods. This study will provide a unique opportunity to assess exposure to COVID-19 in this population over time and document the impact of COVID-19 on service delivery to PWID before and during the trial period.
- Second, even if the intervention does improve outcomes, it may be that the improvement is negligible, or that it is not cost-effective. This is why the study has built-in comprehensive costing and cost-effectiveness analyses. The peer navigation provided in the active control arm is to equal what is provided in the intervention arm; this will allow us to evaluate the marginal effects of integrated care in the mobile unit supported by navigation compared to navigation to community-based services without direct provision of care. We may find that most of the benefit seen in the intervention arm is due to peer support and navigation alone; this would provide policy makers with useful cost-benefit data. Important data will also come from analysis of outcomes at 52 weeks, six months after the conclusion of the intervention. These data will inform policy makers about the durability (or lack thereof) of any gains seen in the intervention arm and/or control arm, particularly whether navigation and/or mobile unit-based care need to be provided long term, or whether these interventions can be effective by “jump-starting” long-term recovery with a transition to community-based services.
- Last, if the mobile unit intervention proves to be successful, documentation of this effect in a randomized controlled trial (RCT) will be helpful for integrating this intervention into public health guidelines.

Ensuring High-Risk in the Sample. In order to enhance the sample for participants with greater risk of HIV acquisition and transmission, and to ensure that sub-groups of PWID who might otherwise be recruited at disproportionately low numbers are adequately represented in the sample, recruitment targets have been included for young and female participants.

- *Women*. Approximately 30% of PWID with OUD diagnosis are women[29,30] but studies of PWID show that women who inject opioids historically are younger than men, progress more rapidly to addiction, and access treatment at lower rates than men,[31] largely because the services provided do not address their needs.[31] Once provided appropriate services,

however, women achieve treatment benefits at the same rates as men.[32] To ensure that the efficacy and implementation science results of this study reflect the experiences of women (who might otherwise be enrolled in small numbers) this study will target enrollment of 25% women regardless of HIV status.

- *Younger PWID*. The median age for PWID in the US [30] is in the mid-40s, but those 18-29 years engage in HIV risk behaviors (sharing injecting equipment or unprotected sex) at substantially higher rates than PWID in older groups.[33] The group of people with OUD least likely to receive treatment are also young (under 26).[34] To enrich our sample for persons at higher risk of acquiring or transmitting HIV and having untreated OUD, we will aim to enroll 25% of participants younger than 30 years (approximately 20% of PWID are younger than thirty years) regardless of their HIV status.[30]

The Importance of Documenting Study Processes and Procedures. There is increasing recognition that mobile care is an approach that policy makers should be considering. In a review of 51 studies published in 2017, the conclusions regarding the effects of using mobile health clinics (MHCs) to deliver healthcare shows a consistent signal that: "…mobile health clinics are successful in reaching vulnerable populations, by delivering services directly at the curbside in communities of need and flexibly adapting their services based on the changing needs of the target community. As a link between clinical and community settings, MHCs address both medical and social determinants of health, tackling health issues on a community-wide level. Furthermore, evidence suggest that MHCs produce significant cost savings and represent a cost-effective care delivery model that improves health outcomes in underserved groups."[35] Convincing evidence from this trial would come at a time when policy makers are considering mobile clinics for public health practice. Moreover, data collected during the pre-implementation and implementation phases of the trial will be used to assess the impact of COVID-19 on availability and access to services delivered in the community and as delivered through the mobile units at study sites.

This study will provide valuable information to policy makers in the four main areas that have been recognized as presenting substantial limitations to using mobile health clinics to deliver integrated care services: (1) fragmentation of care; (2) financial issues; (3) spatial and structural limits; and (4) logistical challenges. This project will directly **address fragmentation of care** by providing integrated health services. As well, findings from the implementation science portion of this work will **advise financial issues** necessary to mount and maintain using mobile medical units within a health jurisdiction. Mobile health clinics are, indeed, tight quarters. In preparing the mobile health units, it will be key to provide for spaces that can be allotted for medical purposes, while also providing some spaces for consultations. The project will **identify the need for additional spaces and creative approaches** that can be used to solve space issues (e.g., parking the mobile unit next to a facility that can provide quiet and private spaces for navigation sessions). The project will also **advise methods for addressing logistical challenges**, (e.g., portable generators to maintain refrigeration and other requirements, using WiFi "hot spots" for constant internet access, etc.).

Finally, the rationale for the study is that the use of a mobile unit is considered best practice for the hard-to-reach population of PWID who are not in drug treatment. There are examples of these types of integrated care strategies, offered in mobile units, that are considered best practices in the recently published NASEM report, *Opportunities to Improve Opioid Use Disorder and Infectious Disease Services: Integrating Responses to a Dual Epidemic.*[26] Perhaps providing most compelling evidence, best practices highlighted in this report developed and implemented integrated strategies for addressing HIV, addiction medicine, primary care, harm reduction,

stigma, with two of the best practices (one in Lawrence, Massachusetts) using a mobile unit (see section 1-8 of the NASEM report).

This evidence, both quantitative and qualitative, provides strong justification for using a mobile unit approach for HPTN 094. The extent to which reductions in barriers of distance and stigma will produce superior outcomes compared to an enhanced control condition that includes active navigation will inform policymakers in decisions about whether to adopt mobile health delivery systems in their jurisdictions. The implementation science aspect of the protocol will provide cost effectiveness data that will equip policymakers with cost data to advise implementing and maintaining a mobile health unit providing integrated care strategies in their jurisdiction.

This integrated strategies approach recognizes the key link between rapid, coordinated and sustained engagement in both MOUD and HIV prevention and care to achieve relevant HIV and substance use outcomes.[26] The study will use two dependent variables: MOUD use and use of antiretroviral (ARV) drug regimens for HIV treatment and prevention (ART for HIV-positive participants; PrEP for HIV-negative participants). These outcomes will be documented by laboratory testing.

Summary. The national opioid epidemic is a crisis that threatens to feed a renewed HIV epidemic. A threat of this nature requires that strategies to address the crisis have goals that are as ambitious as the threat is grave. To that end, the goal of this study is that this intervention will prove so efficacious that it will change policy in local, state and national jurisdictions responsible for addressing clinical practice for PWID with OUD in urban settings. Findings will document the prevalence of SARS-CoV-2 seropositivity in this population at baseline and over the course of the study and will provide insights about the impact of the COVID-19 epidemic on this group affected by intertwined HIV and opioid epidemics.

Audacious Goals. The ambitious goals for this study are as follows: that 50% of participants in the intervention arm are on MOUD at 26 week, with 40% on MOUD at 52 weeks; that the death rate in the intervention arm is reduced by half, compared to the active control arm; and that twice as many participants in the intervention arm are virally suppressed or on PrEP compared to the active control arm.

1.2.1 Implementation Science Framework

The implementation science component of this study provides the opportunity to determine how to deliver this intervention successfully and with fidelity in different US settings (mid-Atlantic, South, West) that are affected differently by the opioid, HIV and COVID-19 epidemics. Attention to each of the elements of the integrated strategy is vital for the health of people living with OUD who have HIV or are at risk for HIV, and have STIs, hepatitis, and other chronic health conditions. However, implementing an integrated care delivery strategy is complex, especially in settings where there can be different levels of access to addiction treatment, HIV services, harm reduction, and primary care services for PWID. We will leverage a hybrid type 1 implementation study design, where we will evaluate how factors affect the implementation of the intervention, and the primary and secondary study outcomes; we will also develop a database that will inform a more rapid translation of study results into public health practice.[36]

The Practical, Robust Implementation and Sustainability Model (PRISM) framework[37] will guide the implementation evaluation in this study. PRISM integrates theory for new behavior adoption (Diffusion of Innovations model); for chronic disease processes like OUD and HIV (the Chronic Care model); and for continuous program improvement (the Model for Improvement).

PRISM will allow evaluation of multi-level factors that influence the implementation process including, (i) how the interventions in both arms are perceived by providers and patients, (ii) characteristics that influence the delivery and receipt of the intervention in both arms, (iii) community-level factors in the external environment that may influence how the intervention is accessed, (iv) systems- and policy-level factors that affect the infrastructure and sustainability of the intervention, and (v) how COVID-19 affects access to the mix of integrated services of MOUD, HIV care (ART, PrEP), STI and hepatitis treatment, primary care, and harm reduction at the systemic and individual level. Finally, PRISM allows evaluation of the implementation outcomes of the intervention through the RE-AIM model (reach, effectiveness, adoption, implementation, maintenance).[38]

This implementation science framework will use a mixed method approach leveraging data systematically captured from routine community engagement, fidelity monitoring, and efficacy evaluation procedures. These data will be augmented by qualitative data collected at each site from participants, providers of integrated health care in the mobile unit and key community stakeholders.

2.0 STUDY OBJECTIVES

2.1 Primary Objective

To evaluate whether 26 weeks of "one stop" integrated health services delivered in a mobile unit, supported by peer navigation, improves use of MOUD, increases rates of viral suppression among HIV-positive individuals, and increases use of PrEP among HIV-negative individuals, as measured at 26 weeks, when compared to 26 weeks of peer navigation to similar health services available at community-based agencies.

2.2 Secondary Objectives

1) To evaluate whether 26 weeks of "one stop" integrated health services delivered in a mobile unit, supported by peer navigation, compared to 26 weeks of peer navigation to similar health services available at community-based agencies:
    a) improves use of MOUD at 52 weeks
    b) increases rates of viral suppression among HIV-positive individuals at 52 weeks
    c) increases use of PrEP among HIV-negative individuals at 26 and 52 weeks
    d) decreases opioid and polysubstance use at 26 and 52 weeks
    e) decreases prevalence of bacterial STIs at 26 and 52 weeks
    f) decreases fatal and non-fatal overdose events by 26 and 52 weeks
    g) increases the proportion of participants with undetectable HCV RNA at 26 and 52 weeks among those with chronic HCV infection at Enrollment
    h) decreases HCV incidence at 52 weeks, for those who are HCV negative at Enrollment

2) To evaluate whether 26 weeks of "one stop" integrated health services delivered in a mobile unit, supported by peer navigation, increases MOUD use, viral suppression, and PrEP use at 26 and 52 weeks compared to enrollment

3) To evaluate whether 26 weeks of peer navigation to similar health services available at community-based agencies increases MOUD use, viral suppression, and PrEP use at 26 and 52 weeks compared to enrollment

4) To assess the prevalence of SARS-CoV-2 seropositivity at baseline, 26 and 52 weeks

5) To document the impact of the COVID-19 epidemic on participants' experiences of seeking, obtaining and/or maintaining health services, housing, food security and drugs

6) To evaluate implementation of "one-stop" integrated health services using a mobile unit, supported by peer navigation, across study sites to identify mechanisms at multiple levels to:
   a) Guide real-time improvements and refinements in the conduct of the study to ensure primary and secondary outcomes are met with fidelity
   b) Examine the quality and process of services delivered in each study arm, particularly as these affect primary and secondary outcomes
   c) Develop evidence-based guidance for policymakers on the uptake and implementation of integrated health services using peer navigation and mobile health units in urban US regions to address HIV in PWID
   d) Identify factors that enhance or impede the delivery of integrated health services using a mobile unit, supported by peer navigation, on primary and secondary outcomes, including responding to the impact of COVID-19 on service delivery

7) To use mathematical modeling methods to:
   a) estimate the effect of integrated health services delivered in a mobile unit, supported by peer navigation, on reducing HIV incidence in PWID and their sexual and injection partners
   b) estimate the cost-effectiveness of the integrated health services provided in a mobile unit and supported by peer navigation

2.3 Process Objectives

To assess among participants in the intervention arm:
1) time to provide MOUD treatment, ART (among those who are HIV positive and not on ART at enrollment) and PrEP (among those who are HIV-negative at Enrollment)
2) the proportion of participants linked to community-based MOUD, ART and PrEP services at 26 weeks

2.4 Exploratory Objectives

Stored samples will be used to analyze HIV subtypes/strains, HIV drug resistance, and the duration of HIV infection. Phylogenetic methods will be used to evaluate behavioral, demographic, and clinical factors associated with viral clusters and transmission dynamics. Stored samples may also be used to characterize HCV strains and the relationship between HIV and HCV infections, and to explore issues related to COVID-19.

3.0 STUDY DESIGN

INTEGRA will be a two-arm, individually randomized, unblinded longitudinal study. The study will be conducted in five US cities selected to represent the geographic diversity of the country's opioid epidemic; for having an appropriate population in sufficient numbers for timely enrollment of the study; and for the ability to implement the study successfully. All potential participants will be screened and enrolled (if eligible) in a mobile unit. The mobile unit will be configured as a mobile clinic, staffed by clinicians licensed to provide MOUD and other care, and will travel to "hot spots" in the community frequented by PWID. Clinicians providing care in the mobile unit

will do so under the auspices of the institution or clinical group with which the research site is affiliated, typically a university health system or medical school. Screening and enrollment procedures, which will take place over a minimum of two visits, will include providing informed consent, obtaining opioid use and treatment history, answering questions about current and past health, and obtaining urine, blood and swab samples for laboratory tests. All enrolled participants will be PWID with OUD (verified by diagnostic interview) who are not on MOUD. Following randomization, participants in the intervention arm will be offered peer navigation and treatment for OUD (MOUD) integrated with HIV services (ART for HIV-positive participants, PrEP for HIV-negative participants) and additional health services in a mobile unit for 26 weeks. In the active control arm, participants will receive peer navigation to community-based services for the same services as the intervention arm for 26 weeks. Approximately 460 HIV-positive and 400 HIV-negative participants will be enrolled (860 total, 430 in each arm), with a target minimum 25% women, and 25% participants under 30 years of age.

The services that participants in both arms receive during the 26 weeks of the intervention will be provided on a schedule determined by the needs of the participant. At baseline, all participants will be seen in the mobile unit to complete the consent process and interview and to provide specimens for laboratory testing. Participants in the intervention arm will receive immediate MOUD, ART, PrEP, STI testing and treatment, hepatitis and pregnancy testing, and primary health services, and navigation to promote uptake of clinical services offered in the mobile unit. Before or at the start of a visit, participants will be assessed for COVID-19; if SARS-CoV-2 infection is suspected, the visit will be deferred to a later date and the participant will be referred for further evaluation and/or care, as indicated and available. Intervention arm participants will also receive immediate navigation to health services or diagnostic tests not available in the mobile unit, and to successfully transfer all care to existing facilities in the community by 26 weeks. Participants in the active control arm will receive navigation through 26 weeks to community-based services for all health services, including MOUD, ART, PrEP, STI testing and treatment, HCV treatment, primary health services and COVID-19 evaluation, testing and care. Peer navigation will be delivered by a cadre of staff called peer navigators; these staff will have been in recovery for OUD for at least a year and will have been trained and certified to provide recovery coaching (support and assistance to help others initiate and adhere to MOUD) and health systems navigation.

Laboratory testing will be performed retrospectively to assess MOUD, ART and PrEP use using samples collected at 26 weeks (primary endpoint) and 52 weeks (secondary endpoint) to assess the initial and durable effects of the intervention. Laboratory testing will also be performed retrospectively to assess substance use, HIV incidence, HCV incidence (52 weeks), prevalence of bacterial STIs, and prevalence of SARS-CoV-2 seropositivity (baseline, 26 and 52 weeks). Results of retrospective testing will not be returned to the sites or participants. Results of point-of-care testing for MOUD and substance use testing at baseline, 26 and 52 weeks will be provided to participants.

A graphical depiction of the study design and randomization scheme is provided in Figure 1 above. See Appendix I for a schedule of study visits and procedures.

3.1 Participating Sites/Institutions

The five sites participating in this study are all in US cities and are listed in the Study Specific Procedures (SSP) Manual.

3.2 Study Duration

Study duration is approximately three and a half years. The study will be initiated at all sites over a six-month period. Accrual is anticipated to occur over two and a half years with individual participants followed on study for approximately one year (26 weeks receiving the intervention or peer navigation, followed by an evaluation for durability of effect at the end of 52 weeks).

4.0 STUDY POPULATION

Approximately 860 PWID with OUD not in MOUD will be included in this study, with a target of a minimum 25% women and 25% aged 18-29. Approximately 460 HIV-positive and 400 HIV-negative participants will be enrolled. Participants will be selected for the study according to the criteria in Section 4.1 and 4.2 and the guidelines in Section 4.4. Participants will be recruited, screened, and enrolled as described in Section 4.3 and assigned to intervention or active control group as described in Section 8.5. Requirements related to participant retention and withdrawal from the study are described in Sections 4.5 and 4.6, respectively.

4.1 Inclusion Criteria

Adults who meet all of the following criteria are eligible for inclusion in this study:

- 18 to 60 years of age
- Urine test positive for recent opioid use and with evidence of recent injection drug use (“track marks”)
- Diagnosed with OUD per Diagnostic and Statistical Manual of Mental Disorders (DSM)-5
- Able and willing to give informed consent
- Willing to start MOUD treatment
- Able to successfully complete an Assessment of Understanding
- Self-reported sharing injection equipment and/or condomless sex in the last three months with partners of HIV-positive or unknown status
- Able to provide adequate locator information
- Confirmed HIV status, as defined in the HPTN 094 SSP Manual

4.2 Exclusion Criteria

Adults who meet any of the following criteria will be excluded from this study:

- Received MOUD in the 30 days prior to enrollment by self-report
- Co-enrollment in any other interventional study unless approved by the Clinical Management Committee (CMC)

Persons who are otherwise eligible to be enrolled will be deferred if they are suspected to have COVID-19; the period of deferral will be determined based on criteria for discontinuation of isolation per US Centers for Disease Control and Prevention (CDC) guidelines or applicable local guidelines. Depending on the length of deferral, screening procedures may have to be repeated to establish eligibility.

Note: The CMC is a sub-group of the Protocol Team that includes physicians from sites, the Protocol Chair and Co-Chair and the DAIDS Medical Officer. Additional roles of the CMC are

addressed in sections 7, 10 and 11 of this protocol and will be further described in the SSP and the CMC charter.

4.3 Recruitment Process

Sites will be responsible for developing appropriate recruitment processes that are geared toward their respective local communities. All advertising materials must undergo approval by the Institutional Review Board (IRB).

Recruitment of persons who inject opioids who are living with HIV or who are HIV-negative and at high-risk will need attention at regular intervals to ensure adequate enrollment for the study in the time allotted. A recruitment plan will be developed at each site to guide outreach to and enrollment of participants. Outreach activities will be consistently conducted at "hot spots" - facilities and venues where persons who inject opioids are commonly found and can be contacted for possible enrollment in the study.

Hot spots will be identified and mapped at each site prior to the start of the project. Examples of locations that are common hot spots include venue-based areas (locations outside jails, criminal justice community supervision programs, syringe exchange sites, detox centers, emergency rooms and hospitals) and street-based areas (tourist areas, parks, strolling areas, skid row, public housing). Hot spots will change over the course of the study as groups of PWID who are not receiving MOUD migrate to new locations due to changes in the local environment (e.g. policing patterns, gentrification). Each site will therefore need to continually review their recruitment data and other information such as the productivity of hot spots in yielding enrollments.

Plans for recruitment at each site will be detailed (and revised) in the SOPs and/or recruitment plan. Sites will be expected to use local data (e.g., heat maps of overdoses) and consultation with local experts to develop their recruitment plan and keep it updated. Local experts consulted may include 1) public health department officials (especially those responsible for conducting National HIV Behavioral Surveillance [NHBS] and for managing publicly-funded MOUD), 2) law enforcement, 3) harm reduction leaders and advocates, 4) health care providers (particularly infectious disease clinicians, emergency room physicians and hospitalists), 5) medical examiners/coroner's office, 6) scientists who are involved in studying PWID in the area and 7) community members. As hot spots are identified, this information will be recorded and maintained through the life of the study to provide a running list of environmental features (e.g., parks, "broken windows," cheap motels, convenience and liquor stores, marijuana dispensaries, gentlemen's clubs, etc.) where PWID who are not receiving MOUD may congregate.

Hot spots will serve not only as sites for outreach and recruitment, but also as sites where the mobile unit may be deployed to implement the study.

In addition to recruiting from identified hot spots we will employ peer recruitment and referral strategies. Participants will be encouraged to recruit their peers from their social network, from other PWID and from their communities. We will also encourage referrals from providers such as emergency departments, alternative-to-incarceration programs, homeless shelters, harm reduction programs, public health departments, etc.

As noted in Section 1.2 of this protocol, a minimum of 25% women are planned for enrollment in this study and a minimum of 25% under age 30 years. Sites may propose additional recruitment strategies needed to yield the expected minimums of women and youth for the study.

4.4 Co-Enrollment Guidelines

In general, participants in this study will not be allowed to take part in other concurrent interventional research studies during their study participation, unless approved by the CMC. This is due in part to concerns about participant study burden and to avoid confounding in the interpretation of the study data. This concern is particularly relevant for co-enrollment in studies of long-acting HIV care and prevention products. Given this situation, the CMC should be consulted for any possible exceptions, including participation in observational studies.

4.5 Participant Retention

It is expected that this population under study (persons who inject opioids who are not in MOUD treatment) will present challenges exceeding those encountered in traditional HPTN protocols. As such, retention activities will require additional attention and resource to ensure that 90% retention targets at 26 and week 52 visits are met. The following activities, strategies and perspectives are required in some combination to ensure participants are found throughout the study period and especially at distal follow-up visits.

Study site staff are responsible for developing and implementing local standard operating procedures (SOPs) to achieve the 90% retention goal. Components of such procedures may include:

- Thorough explanation of the study visit schedule, the importance of keeping each study visit, procedural requirements during the informed consent process and re-emphasis of the importance of these visits and requirements at each study visit.
- Thorough explanation of the importance of both study treatment groups to the overall success of the study.
- Collection of detailed and valid locator information at the study Screening Visit, with active review and updating of this information at each subsequent visit.
- Providing a small incentive for regular locator information updates or high study retention.
- Soliciting consent to contact providers, such as physicians, social workers or nurses, when trying to locate the participant.
- Use of mapping techniques to establish the location of participant residences and other locator venues at Screening and at each visit to monitor changes in living situations.
- Use of appropriate and timely visit reminder mechanisms.
- Immediate (as soon as recognized), consistent (until a response is gotten) and multifaceted (all contacts reached) follow-up activities on missed visits.
- Mobilization of trained outreach workers or "tracers" to complete in-person contact with participants at their homes and/or other community locations.
- Regular communication with the study community at large to increase awareness about HIV/AIDS and explain the purpose of HIV prevention research and the importance of completing research study visits.

In addition to the procedures described, which are standard for all HPTN studies, an important aspect that will facilitate retention of this population will be to ensure that all study staff, and particularly clinicians and peer navigators, display a non-judgmental, supportive attitude toward PWID in general and study participants specifically, and that they build genuine rapport with participants during study encounters in both arms.

It will also be important for study staff to establish and maintain rapport and good will with the community of PWID and with those who work with PWID in the local setting. This will facilitate reconnecting with participants who have missed visits or become lost to follow up, an inevitable eventuality when working with this population.

4.6 Participant Withdrawal from Study

Participants may voluntarily withdraw from the study for any reason at any time. The Investigator of Record (IoR) or designee also may withdraw participants from the study in order to protect their safety and/or if they are unwilling or unable to comply with required study procedures after consultation with the Protocol Chair, DAIDS Medical Officer, SDMC Protocol Statistician, and LOC Clinical Research Manager (CRM).

Participants also may be withdrawn if the study sponsor, government or regulatory authorities, or IRB terminate the study prior to its planned end date.

Every reasonable effort will be made to complete a final evaluation (as described in Section 6.5 and Appendix I) of participants who terminate from the study prior to the Week 52 visit, and study staff will record the reason(s) for all withdrawals from the study in participants' study records.

5.0 STUDY INTERVENTIONS

Participants in both study arms will receive initial medical assessment and services in the mobile unit during Screening and Enrollment (see Section 5.1 for details). Following enrollment and randomization:

- Those assigned to the intervention arm will receive medical services for OUD (MOUD), HIV treatment or prevention (ART or PrEP), and other primary care conditions that can be managed in the mobile unit (see Table 2 below). They will also receive 26 weeks of peer navigation. Although medical services will initially be based in the mobile unit, a goal of peer navigation will be to successfully transition participants to services at existing facilities in the community by the end of 26 weeks.
- Those assigned to the active control arm will receive 26 weeks of peer navigation to the same medical services delivered at existing facilities in the community.

At the end of 26 weeks, participants in both arms will have study visits for assessment of primary and secondary endpoints. At the end of 52 weeks, participants in both arms will have a final study visit for assessment of additional secondary endpoints. Table 1 (p.32) summarizes services that will be provided to participants in the two arms. Table 2 (p.36) provides details of the medical services that will be provided in the mobile unit to intervention arm participants.

5.1 Initial Assessment and Provision of Services in the Mobile Unit - All Participants

Engagement with persons interested in participating in the study will begin with the informed consent process. To protect the safety of community members and staff, sites may implement a pre-screening assessment for COVID-19 before beginning study screening procedures. These procedures may take place remotely (e.g. over the telephone) or outside of the mobile unit, per

guidelines. Those suspected to have COVID-19 will be deferred from screening and referred for testing and/or treatment, as appropriate and available.

Once informed consent has been obtained, participants will begin screening procedures and, if appropriate, enrollment into the study. As part of Screening and Enrollment, all participants will receive assessment by a clinician in the mobile unit and point-of-care testing for OUD, HIV, and pregnancy (for those who can become pregnant). Samples will also be collected for laboratory testing for HIV, HCV, HBV, HAV, STIs, and hematology/chemistry at this time. Those who test HIV positive will receive additional testing as appropriate (CD4 cell count, viral load, resistance testing). At enrollment, basic medical services will be provided to all participants, including empiric treatment for STIs if symptoms are present, provision of harm reduction services (naloxone kits and, where possible, syringe/needle exchange services), and preparation of a treatment plan. During the Enrollment Visit, all participants will be assessed for OUD diagnosis per the DSM-5, will asked about any MOUD treatment in the past 30 days and will meet with their peer navigators for the first time. Persons with suspected COVID-19 will be deferred from enrollment until they meet the criteria for discontinuation of isolation per CDC guidelines or applicable local guidelines and will be referred for COVID-19 testing and treatment services, as appropriate and available. The specific procedures for assessing participants for COVID-19 will be described in the SSP Manual. Following the Enrollment Visit, navigation, medical and counseling services will be provided by study arm as described below.

5.2 Provision of Medical Services

5.2.1 Medical Services - Intervention Arm

For the 26 weeks of intervention following study enrollment and randomization, participants assigned to the intervention arm will receive medical services in the mobile unit from a clinician. First and foremost, participants will be provided immediate access to MOUD to control withdrawal symptoms quickly and thereby minimize the disorganizing behavioral effects of addiction that jeopardize adherence to HIV treatment or prophylaxis regimens. The MOUD regimen provided in the mobile unit will be influenced by the participant's recent drug use and treatment history, the drug regimens used in local opioid treatment programs, and availability of medications on the mobile unit (i.e., a restricted range of medications will be available on the mobile unit; a wider range of MOUD options will be available via navigation/linkage to regularly-available resources at local clinics). The clinician in the mobile unit will have the necessary medical waiver (Drug Addiction Treatment Act- 2000 "X" waiver) and clinical experience to provide MOUD to this population.

For those identified as HIV positive during Screening and Enrollment (and not already established in ART elsewhere), and for those who acquire HIV infection during the intervention period, ART will be provided in the mobile unit. Those identified as HIV negative at Enrollment will be provided risk reduction counseling and offered PrEP according to current CDC recommendations; these participants will be tested for HIV infection during the intervention period. Clinical care including applicable laboratory test monitoring for HIV-positive participants and those on PrEP will be provided per national clinical guidelines. Over time, these participants will be linked to appropriate long-term ART or PrEP service settings.

Based upon the results of testing for HAV, HBV and HCV at Enrollment, participants will meet with their navigators to receive facilitated referral to the most appropriate community-based services in their area for vaccination and/or treatment, anticipated to be Federally-Qualified Health Centers (FQHCs) and clinics with experience in providing hepatitis services for PWID.

An exception to this will be for HBV treatment, where ART or PrEP regimens provided in the mobile unit can also treat HBV infection, if determined to be appropriate by the medical provider.

STI treatment (or referral for cases that cannot be addressed in the mobile unit) will be provided for those with laboratory-confirmed infection from samples collected at Enrollment (testing for GC/CT and syphilis); at all visits, participants with STI symptoms will be offered empiric treatment. Sites will use local guidelines for interpretation of test results for clinical management. To determine syphilis prevalence, syphilis results will also be evaluated retrospectively at the HPTN LC to differentiate active infection from past exposure.

Assessment and/or treatment of other basic medical conditions will be provided in the mobile unit for participants in the intervention arm (see Table 2). Treatment or management of more complex conditions will require referral. In this population, it is expected that few participants will already be successfully engaged with health services providers in their community. However, if a participant does prefer to receive some or all of their care from community-based services instead of the mobile unit (for example, if they are already receiving HIV care from a clinic in the community), that will be allowed and supported by peer navigation.

Clinicians on the mobile unit will assess participants in the intervention arm for COVID-19 during the first 26 weeks. Participants with suspected COVID-19 will be referred for services in the community and facilitated in accessing those services.

Participants who also use stimulants (methamphetamine, cocaine) in addition to injection opioids, will be referred to 12-step meetings available locally such as crystal meth anonymous, narcotics anonymous and alcoholics anonymous. Participants will be assisted in finding counseling services for evidence-based behavioral treatment, where available.

Harm reduction services (naloxone kits, and where possible, needle/syringe exchange) will be available on the mobile unit, as will condoms and lubricant. We will collaborate with local community-based organizations providing such services to augment what is offered in the mobile unit. Peer navigators will offer condoms and lubricants during their meeting with participants.

5.2.2 Medical Services - Active Control Arm

Except for the services described above in Section 5.1, medical services for participants in the active control arm will not be provided in the mobile unit but will be provided by the existing facilities in the local area (referred to in this protocol as "community-based services") according to the local standard of care (SOC). Throughout the 26 weeks, linkage provided through peer navigation will assist participants in the active control arm to access, at local clinics and agencies, the full range of health services that intervention arm participants will be provided on the mobile unit or be navigated to. Peer navigators will offer condoms and lubricants during their meetings with participants.

5.3 Provision of Peer Navigation Services

Peer navigation is intended to assist people who may not have ready access to services to overcome barriers and to enter care and remain in treatment. These services will be provided by a cadre of trained study staff designated as peer navigators who will act both as health system navigators (assisting participants to access care in a fractured health system) and as peer recovery

coaches with lived OUD experience (assisting participants to initiate and sustain MOUD treatment).

Peer navigators will motivate and assist participants to successfully enroll in and consistently use MOUD, ART, PrEP, as well as to receive STI testing and treatment, hepatitis vaccine or treatment, SARS-CoV-2 testing (if available), and referral for services, primary care and harm reduction services. It is anticipated that many participants will be eligible to receive medical insurance or other government support for their medical care and treatment. Peer navigators will help participants sign up for/access health insurance or other programs for which they are eligible, especially if no insurance is available. Depending on participant need, navigation services will include identifying appropriate health service providers in the community, arrangement of medical appointments, reminding participants of upcoming appointments, assisting with transport or accompaniment to appointments (whether in the mobile unit or at community-based services), and transfer of medical records. Although assistance with obtaining health care services and adhering to treatment/prophylaxis regimens will be the primary focus of peer navigation, peer navigators will also provide referrals to local resources for participants who have needs related to food security, housing and employment.

Participants in both arms will work with a trained peer navigator for the first 26 weeks on study. It is expected that initial interactions between the peer navigator and a participant will be conducted in person, beginning with the Enrollment Visit in the mobile unit. For participants with a phone, it is expected that the peer navigator will use messaging apps/texts to maintain regular contact with participant, confirm appointments, locate those who have missed scheduled visits, etc. throughout the 26 weeks of navigation (cell phone minutes will be provided). To the extent possible and depending on local availability, navigators will assist with temporary shelter placements for those who need them and will provide transport vouchers (e.g., bus tokens) to enable participants to access services and remain in care.

The frequency and timing of navigation encounters will be measured for both arms in order to monitor dose by condition in number of sessions, minutes spent per encounter and types of activities, referrals and services completed by the navigator. The study staff supervisor will meet with the navigators on a weekly basis in both conditions and provide support for navigators as described in the SOP. Rather than set a required "dose" of navigation for each arm, monitoring implementation of navigation will document whether equipoise is maintained between conditions through the trial when evaluating efficacy of the mobile unit on primary and secondary outcomes, and when evaluating improvements in outcomes for both conditions compared to baseline.

5.3.1 Navigation Services - Intervention Arm

Peer navigators in the intervention arm will assist their participants to access medical services provided from the mobile unit. They will also assist their participants to obtain care from community-based services for those tests or services that cannot be addressed in the mobile unit. Initial visits between the participant and their navigator are expected to occur in person, beginning with introductions at the Enrollment Visit in the mobile unit. As the participant-navigator relationship becomes more established, interactions can be expected to take place at locations other than the mobile unit and may occur by phone or by messaging app/text. The peer navigator will also be responsible for working with the participant during the intervention period to prepare them to transition to community-based services as the 26 weeks of study-provided care in the mobile unit comes to an end.

5.3.2 Navigation Services- Active Control Arm

The role of the peer navigator for active control arm participants will be to link them to community-based services for the same types of medical services provided in the mobile unit in the intervention arm, i.e., MOUD, HIV treatment (ART) and prevention (PrEP), vaccination or treatment for hepatitis, STIs testing and treatment, SARS-CoV-2 testing (if available), and referral, primary care and harm reduction services. As described for the intervention arm, initial meetings between the participant and the navigator will be in-person. As the participant-navigator relationship becomes more established, interactions can be expected to occur by phone or by messaging app/text.

5.4 Drug Supply

All of the drugs that will be provided as part of services in the mobile unit will be US Food and Drug Administration (FDA)-approved medications in common use for these indications; this study does not involve any investigational study products. Drug supply and management will be an important issue for this study since a core component of the intervention is provision of drugs for MOUD, ART and PrEP. It is anticipated that it will be possible to obtain these drugs through manufacturer donation to the study, and that manufacturers' donations will be received and distributed to the sites by a qualified contractor. Once the contractor has been selected, policies and procedures for management, storage, distribution, dispensation and accountability will be established for the study and documented in the SSP Manual and site SOPs. Donated product will be received by the research pharmacies at the CRS, who will be responsible for local storage, dispensation and accountability. Because the MOUD medication expected to be used in this study will be buprenorphine, a schedule III controlled substance, the study team will work with the US Drug Enforcement Administration to obtain approval for dispensation of buprenorphine from the site pharmacy to participants on the mobile unit. Study sites are responsible for procuring antibiotics locally for STI treatment for the mobile units.

Table 1- Summary of Services Provided to the Intervention and Active Control Arms

Formatted: Numbering: Continuous

**Service**		**Summary**
**Diagnostic Testing**	**Screening/ Enrollment**	Screening and Enrollment will occur on the mobile unit for all participants.<br>**<u>Screening Visit:</u>**<br>• HIV rapid testing<br>• Urine dip stick testing for substances of abuse and MOUD.<br>**<u>Enrollment Visit</u>:**<br>**In both arms**,<br>• HIV rapid testing performed for HIV<br>• Rapid pregnancy testing, as appropriate<br>• Collection of blood, urine and swab specimens for local laboratory testing for HIV, STIs, HAV/HBV/HCV, and hematology/chemistry assessments. Laboratory results will not be immediately available.<br>**In the intervention arm,** laboratory results should be provided at the next mobile unit care visit, within days of enrollment. If this is not possible, laboratory results will be conveyed by a navigator, clinician or trained staff member, as appropriate, in person or by phone, medical app, or letter.<br>**In the active control arm**, laboratory results will be conveyed by navigator, clinician or trained staff member, as appropriate, in person or by phone, medical app, or letter.
	**After Enrollment**	**<u>Between Enrollment and week 26</u>**<br>**In the intervention arm,**<br>• As needed, further/repeat testing for clinical care related to MOUD, HIV, STIs, hepatitis or pregnancy.<br>**In the active control arm,** no diagnostics will be performed in the mobile unit or as part of the study during this time (all care & diagnostics will need to be provided from community-based services).<br><br>**<u>At week 26 and 52 Visits</u>**<br>**In both arms,**<br>• HIV rapid testing (for those not previously confirmed to be HIV positive)<br>• Rapid pregnancy testing (as appropriate)<br>• Urine dip stick testing performed for substances of abuse and MOUD.<br>• Collection of blood and swabs for laboratory testing for HIV, STIs and (week 52 only) incident HCV infection in those who were anti-HCV negative at study entry. Those with chronic HCV will receive HCV RNA testing at week 52.

		Laboratory results will not be available until after the visit; clinically-relevant results will be conveyed by navigator, clinician or trained staff member, as appropriate, in person or by phone, medical app, or letter.
**Medical Care and Treatment**	**Enrollment**	**Enrollment Visit:**<br>**In both arms in the mobile unit,**<br>• Assessment for COVID-19<br>• Assessment for OUD and confirmation of HIV status.<br>• HIV and MOUD counseling and a basic physical exam.<br>• Assessment for mental health needs by standardized questionnaires<br>• Obtaining a targeted medical history.<br>• Assessment of STI symptoms and provision of empiric treatment as indicated.<br>• Staff will provide information about MOUD therapy, ART and PrEP, and viral hepatitis treatment and vaccination.<br>• Clinician and participant will develop a preliminary care plan.<br>• Condoms, lube and harm reduction will be offered.<br>**For those in the intervention arm**,<br>• The participant will be given instructions on how to prepare for initiating MOUD therapy. In some instances, it may be appropriate to dispense medication to begin MOUD, ART or PrEP at this visit, but it is expected that this will be rare.<br>**For those in the active control arm,** no additional procedures.
	**After Enrollment**	**<u>Between Enrollment and week 26</u>**<br>Condoms, lubricant and harm reduction will be available to all participants<br>**In the intervention arm,** the participant will have visits in the mobile unit as indicated for limited primary care. Results from enrollment tests will be provided. Test results will inform updates to the clinical plan.<br>• Assessment for COVID-19<br>• Establish participant on MOUD, ART (if HIV-positive), PrEP (if HIV-negative)<br>• Treat bacterial STIs<br>• Refer for treatment or vaccination for hepatitis infection.<br>***For additional details on medical care in the mobile unit, see Table 2.***<br>**In the active control arm,** the clinician or other appropriate site staff will provide results from enrollment tests to the participant, typically over the phone, though in-person delivery at a peer navigation visit may also be common. Test results will inform updates to the clinical plan.<br>• The clinician and peer navigator will identify appropriate referrals to community-based services and will provide test results to these services with participant consent.<br><br>**<u>At week 26 and 52 Visits</u>**

		Limited medical services will be provided to participants at the week 26 and 52 visits, which may take place in the mobile unit or another location such as a community clinic or CRS.<br>• Assess for COVID-19<br>• HIV testing and counseling will be provided to those who were not previously confirmed to be HIV positive<br>• Rapid pregnancy testing (as appropriate) and results provided during the visit.<br>• Provision of condoms, lubricant and harm reduction.<br>The results of tests for HIV viral load, STIs, and HCV Ab/viral load, performed on samples obtained at the visit will be provided to participants when available, with appropriate referral to community-based services.
**Peer Recovery Coaching and Navigation**	**At Enrollment**	• All participants will be assigned and introduced to a peer navigator at the Enrollment Visit and together will identify preliminary goals and plans.<br>• The dosage of peer navigation will be titrated to the level necessary to keep the participant engaged in the study, engaged in the medical care and treatment to achieve the goals prioritized in the clinical plan, and able to be located for 26 and 52 week follow-up visits.<br>• Protocol team leaders will provide supervision and guidance to monitor peer navigator use by condition and to provide feedback to navigators to ensure relatively similar levels of navigator session use by condition.<br>• Obtaining insurance or enrollment in other programs to pay for health services will be a top priority.<br>• Once laboratory results from testing of samples collected at Enrollment are available, the preliminary clinical plan will be updated and confirmed.
	**After Enrollment**	**<u>Between Enrollment and week 26</u>**<br>**Intervention Arm**:<br>Peer navigators will<br>• Help the participant obtain (or confirm) health insurance<br>• Identify an appropriate provider and accompany the participant to visits until the participant feels confident in doing this on their own, and provide assistance for transportation costs (e.g. vouchers, bus passes) for any health need that cannot be addressed on the mobile unit<br>• Remind participants of appointments at the mobile unit or other providers, follow-up when appointments are missed, and motivate the participant to follow through on goals<br>• Help the participant fill prescriptions at the pharmacy<br>• Provide encouragement, practical advice and understanding, as a person with MOUD experience, as the participant engages in recovery<br>• Help the participant in the weeks before the end of the intervention, transition from receiving care in the mobile unit by establishing care at community-based services so that when the intervention stops, the participant is already comfortable with the new provider

		**Active Control Arm**: The services provided by the peer navigator in the active control arm will be the same as for the intervention arm except that from the start, the peer navigator will be connecting the participant to services available in the community rather than the mobile unit.

Table 2- Overview of Medical Care Provided in the Mobile Unit for Intervention Arm Participants

Formatted: Numbering: Continuous

This table describes the scope of clinical services provided in the integrated strategies approach on the mobile unit. Details of the timing of tests and activities are provided in Appendices IA-IC.

This is not a comprehensive list of services provided by clinicians in the mobile unit, as clinicians may encounter and respond to many conditions in the course of providing care for addiction, infectious diseases and primary care. The descriptions below set expectations for how primary care clinicians can function in the mobile unit with consistency from site to site, with limits on what will be provided, while enabling them to provide needed attention to the conditions most common to primary care. Referrals will be provided for diagnosis and maintenance of care of most chronic primary care conditions. Referrals will also be provided for conditions that will routinely be managed in the mobile unit (e.g. STI infection) if they require more specialized care (e.g. neurosyphilis).

Condition	Notes
OUD	MOUD will be managed on the mobile unit. Will include dispensation of drugs (pending donation), to include:<br>• Buprenorphine-based medicine (sublingual and injectable regimens)<br>• Long-acting naltrexone<br>Participants who prefer methadone will be referred to community-based services if available
Stimulant Use	Participants who also use stimulants (methamphetamine, cocaine) will be referred to 12-step meetings such as crystal meth anonymous, narcotics anonymous and alcoholics anonymous, and evidence-based behavioral treatment, where available.
HIV-ART	ART will be managed from the mobile unit for those not already in HIV care, including:<br>• Dispensation of one first-line, single-pill regimen to participants for whom this is indicated (pending donation)<br>• Prescription provided for fulfillment at a pharmacy if a different regimen is indicated
HIV-PrEP	PrEP will be managed from the mobile unit including:<br>• Dispensation of one, single pill regimen (pending donation)<br>• Prescription provided for fulfillment at a pharmacy if a different regimen is indicated
HCV Treatment	• Testing will be performed as part of study procedures.<br>• Those who test positive will be referred for treatment.
STI	Bacterial STIs will be managed from the mobile unit including:<br>• Provision of antibiotics for treatment of bacterial STIs tested for as part of the protocol (CT, GC and syphilis)<br>• Providing a prescription for fulfillment at a pharmacy, or a referral to community-available services, for any conditions not able to be treated on the mobile unit
HAV	• Testing will be performed as part of study procedures.<br>• Those without evidence of immunity will be referred for vaccination.
HBV	• Testing will be performed as part of study procedures.<br>• Those without evidence of immunity will be referred for vaccination.

	• Those with evidence of chronic infection who are HIV positive may be co-treated by an appropriate ART regimen dispensed on the mobile unit or through a prescription fulfilled at a local pharmacy.<br>• Those with HBV who are HIV negative will be counseled on the risks/benefits of PrEP.
Mental Health	Clinicians will screen for symptoms of mental health disorders during visits on the mobile unit.<br>• For any issues identified, participants will be referred for further evaluation and/or care at community-based services, facilitated by peer navigation.<br>• If a person appears to be at risk to harm themselves or others, 911 will be called
Pregnancy	Pregnant participants may utilize the mobile unit.<br>• Participants who are pregnant will continue to be seen for MOUD, ART, PrEP and other services on the mobile unit. all pregnant participants will be referred for obstetric care with an OB/GYN provider comfortable treating pregnant women who inject drugs treated with MOUD. Study clinicians will endeavor to coordinate with the obstetric care provider to optimize care.
Reproductive Health	Limited reproductive health services will be provided in the mobile unit<br>• Prescriptions for oral contraception<br>• Condoms/lube
Basic Primary Care	Basic primary care that will be provided on the mobile unit will include:<br>• Diagnosis and management of minor acute illnesses and infections such as upper respiratory tract infections, colds, flu, diarrheal illness<br>• Providing prescriptions for pharmacy fulfillment for such conditions if indicated.<br>• Identifying more complex care needs and referring for further diagnostics and care from community-available services
COVID-19	Clinicians will assess participants for COVID-19 at each encounter. Those with suspected COVID-19 will be referred for further evaluation, care and treatment, as appropriate and available. CDC and local guidelines for discontinuation of isolation will direct when participants with suspected COVID-19 can resume in-person visits. Distance procedures to collect data and monitor health will be implemented to the extent possible.
More Complex Care Needs & Chronic Conditions	• Management of complex care needs or chronic conditions will be referred to community-available services, such as FQHCs or other clinics<br>• Clinicians on the mobile unit may provide prescriptions for medications for chronic conditions that have been lost or stolen or are needed for continuity of care, including communication and coordination, as possible, with the provider managing the care of the chronic condition.

6.0 STUDY PROCEDURES

An overview of the study visits and procedures is presented in Appendix I. Presented below is additional information about specific-study procedures. Additional detailed instructions to guide and standardize all study procedures across sites will be provided in the SSP Manual. Visit windows and visit coding are described in the SSP Manual.

Routine laboratory test results will be provided to participants in a timely fashion. The clinical teams will attempt to report results to participants in the following time frames: routine normal laboratory results will be reported to patients within two weeks, routine abnormal results within two days, and critical abnormal results the same day. Depending on the circumstance and urgency, results may be provided to participants via phone call, medical app, in person or via letter (if participant has an address). The personnel who provide results (both normal and abnormal) will be clinical staff and non-clinical staff (as permitted by local regulations), who have received appropriate training.

We will respond flexibly and in accord with federal and local health policies and guidelines to the challenges presented by an ongoing or recurrent COVID-19 epidemic throughout the study. The accommodations to study procedures listed below reflect our best estimate of the scenarios we might face and what approaches might be possible under such conditions. Any modifications to protocol conduct or pausing of screening, enrollment or follow-up undertaken to address the threat of SARS-CoV-2 infection will be carefully considered by the protocol team in consultation with the sponsor to minimize threats to the integrity of the study and the safety of study participants and staff. As well, we will create a study specific protocol for outlining management of conducting research on HIV and opioid addiction in the context of COVID-19.

6.1 Screening Visit

It is the responsibility of the local site to determine the best approach to screening. For each participant, independent written informed consent will be obtained before any study procedures are initiated. Screening procedures may occur over one or more visits. Enrollment must occur within 30 days of initiation of screening, defined as the date the participant signs the informed consent form. Screening and Enrollment may not occur on the same day.

Sites will follow the HIV testing algorithm for screening included in the SSP Manual. Individuals deemed not eligible will be informed that they do not meet the eligibility criteria for the study and will be referred for appropriate medical care, if necessary.

Potential participants may be rescreened up to two times (three screening attempts total) at the discretion of the IoR or their designee.

To protect community members and staff, it is expected that sites will implement pre-screening procedures to identify persons with symptomatic COVID-19 among those presenting for screening. Those with suspected COVID-19 will be deferred from screening and referred for community-available services/care/treatment.

6.2 Enrollment Visit

Potential participants determined to be eligible at screening will be invited to join the study. Not everyone who is screened eligible may be offered enrollment depending on the need to achieve enrollment targets by sex, age and HIV status. Participants will be considered enrolled once they have been randomized to either the intervention or active control arm. For those in the intervention arm, the activities of the Enrollment Visit will include the beginning of their clinical care in the mobile unit and will be more extensive. For participants randomized to the active control arm, the Enrollment Visit procedures will be fewer but will include the significant step of initiation of peer navigation, the primary benefit they will receive during the intervention period.

When creating a clinical plan for an intervention arm participant, staff will prioritize initiating MOUD to reduce the disruptive effects of addiction as a first step. For the buprenorphine regimens we anticipate using for MOUD in this study, induction requires the participant to be in a state of withdrawal before treatment can begin. It is therefore expected that in the majority of cases, participants will not be ready to begin MOUD until at least one day after Enrollment. But there may be cases in which a participant will be ready to be induced at Enrollment and MOUD will begin that day. This flexibility-- to start MOUD either at Enrollment or an interim visit-- is reflected in the schedule of evaluations (Appendix I).

Similarly, it is expected that most participants in the intervention arm will not initiate ART or PrEP until after they are initiated on MOUD, at an interim visit some days after Enrollment. However, there may be instances where initiating ART or PrEP immediately is a high priority for a participant and is supported by the clinician. In such cases ART or PrEP may be started at the Enrollment Visit. This flexibility in when to start ART or PrEP is reflected in the schedule of evaluations (Appendix I). We also anticipate that some participants may already be on ART, in which case peer navigation will support continuation of ART and HIV care at their existing provider.

As part of enrollment procedures, staff will assess potential participants for COVID-19. Persons who are otherwise eligible to be enrolled will have enrollment deferred if they are suspected to have COVID-19, until they meet the criteria for discontinuation of isolation per CDC guidelines or applicable local guidelines. Depending on the length of deferral, screening procedures may have to be repeated to establish eligibility. Enrollment may need to be paused if COVID-19 conditions dictate responses such as shelter-in-place in the cities where the study is being implemented.

6.3 Clinical Care and Navigation Visits Between Enrollment and Week 26

Between the Enrollment Visit and the week 26 visit, participants in both arms will engage with study staff regularly for peer navigation. Expectations for the frequency of visits between participants and navigators will be spelled out in the SSP manual, but the actual timing, length and content of navigation visits for any given participant will be determined by participant needs rather than a fixed study visit schedule.

In the intervention arm, participants will be seen in the mobile unit as needed to provide medication and to manage adherence to MOUD and ART or PrEP, and to address other participant health needs. These will be considered “care visits”. As for navigation visits, the timing, length, frequency and care provided during clinical visits will be determined by the needs

of the participant and not by a fixed study schedule. As intervention arm participants near the end of the 26 weeks, their peer navigator will facilitate their transition to community-based services.

Select data will be captured into the study database from peer navigation and clinical care visits to document and describe the intervention as delivered (particularly the "dose" of navigation delivered in each arm, to assist with interpretation of primary and secondary outcomes), to assist the study team in monitoring fidelity and comparability of delivery of the intervention during the period of study conduct and to enable the study team to make real-time improvements to intervention delivery during execution of the trial.

It is anticipated that the COVID-19 pandemic will require accommodations to how participant visits with peer navigators or on the mobile unit can be conducted. Enhanced infection control procedures such as wearing of personal protective equipment (PPE), reduced density of persons allowed in and around the unit, and thorough disinfection of the unit between participants will be undertaken. Participants will be assessed for COVID-19 before beginning a visit and deferred from continuing if COVID-19 is suspected. Guidelines for resumption of in-person visits will be per CDC and/or local guidelines for discontinuation of isolation. CDC and local guidelines will be followed regarding quarantine of staff with potential exposure to persons with suspected COVID-19. If the intensification of the COVID-19 pandemic prevents routine in-person clinical care or navigation visits between staff and participants, the study team will use available tools and approaches to continue to provide services to participants, as possible. As of the time of the writing of this protocol, DEA regulations allow participants to be prescribed buprenorphine over the telephone with home induction. If participants have mobile phones, the study will provide airtime minutes to allow participants to continue to receive peer navigation and clinical care via telemedicine, with appropriate training of peer navigators and clinicians in these approaches. Uninterrupted provision of medications for MOUD, ART and PrEP may be instituted through in-person delivery that maintains social distancing while insuring delivery to the prescribed recipient. Measures will be taken to ensure that participants taking ART or PrEP are receiving appropriate clinical follow-up, including periodic laboratory testing. The expectation that donated, long-acting buprenorphine injection will be the preferred offering for MOUD in this study will enable participants to maintain adherence with once-monthly dosing, eliminating the need to remember to take a daily dose during times of heightened disruption to daily routines.

6.4 Week 26 Visit

The week 26 visit marks the end of the intervention period for both arms of the study. Biological samples obtained at this visit will be used for assessment of primary and secondary objectives. For participants in both arms, peer navigation will end at 26 weeks; from this point forward, participants will be responsible for obtaining care from community-based services without further assistance from study staff.

Blood, urine and swabs will be collected from participants for laboratory assessment of HIV infection (in those not previously confirmed to be HIV infected), ART adherence or PrEP use, STIs, SARS-CoV-2 infection, substance use and, for those with a positive HCV antibody test at enrollment, HCV RNA. Results from point-of-care testing (e.g., for HIV and pregnancy) will be provided to the participant at the visit. For all other medical diagnoses or conditions identified during or after the visit, referral to community-based services will be provided. The site will attempt to provide any laboratory results obtained after the visit to the participant and to refer the participant to obtain any medical care/treatment indicated by these results from community-based services.

Although the mobile unit will be prioritized for Screening, Enrollment and provision of intervention services, this research visit may take place in the mobile unit, or at a community clinic or CRS. Conduct of these visits may take place at appropriate locations in the community where participants will feel comfortable, such as the CRS or an affiliated clinic or community space. Should concerns about the COVID-19 epidemic make conduct of in-person visits impossible, week 26 visits may be conducted using telephone/internet visits as scheduled, with collection of biospecimens at a different date. The study team would make every effort in such a situation to minimize the disruption to the study schedule, to collect as much data as possible while prioritizing the health of participants and staff.

6.5 Week 52 Visit

The week 52 visit marks the end of the period on study for all participants. Samples obtained at this visit will be used for assessment of secondary objectives. Blood, urine and swabs will be collected from all participants for laboratory assessment of HIV (in those not previously confirmed to be HIV infected), ART adherence or PrEP use, STIs, SARS-CoV-2, HCV (for those negative at Enrollment), substance use and HCV RNA (for those with a prior positive HCV antibody test). Test results available during the visit will be provided to the participant. The site will attempt to provide any clinically important laboratory results obtained after the visit to the participant (e.g., confirmed HIV, STIs, HCV; results of HIV drug restistance testing) with referral to community-based services. As with the week 26 visit, this research visit may take place in the mobile unit, community clinic or CRS.

As described for the week 26 visit, week 52 visits need to be in-person visits. Also as described for week 26 visits, a concerns related to the COVID-19 epidemic might necessitate making accommodations to complete the week 52 visits using on-line and distance procedures to protect the health of participants and staff, with every attempt to minimize the effects of deferred visits on study data and subsequent interpretation of endpoints.

6.6 Implementation Evaluation and Cost-Effectiveness Data Collection Procedures

Implementation Evaluation Procedures. We will use a mixed methods, theory-driven (PRISM) conceptual model to determine factors that affect delivery of integrated care using a mobile unit, supported by peer navigation, sucessfully and with fidelity in different US settings co-affected by opioid and HIV epidemics. Quantitative and qualitative data will identify with depth and precision the multi-level factors (i.e. patient, healthcare organizaiton and personnel, community environment, structural) that correspond with the fidelity of implementation, effectiveness, and dissemination of integrated health care delivered in a mobile unit including responding to the impact on service delivery imposed by COVID-19. To achieve the implementaiton evaluation objectives, site-level data will be collected across three timepoints: (a) the pre-implementation phase of the study to document baseline status of the local opioid epidemic, the local HIV epidemic, the policies and agencies aligned to address these epidemics through landscape data collection of the site, and to identify factors that may influence the delivery of integrated health services via mobile unit and peer navigation (e.g. site-level access to quality addiction, HIV, and primary care for PWID), (b) the implementation phase of the study to collect data on factors that promote or impede the implementation of the intervention (in the mobile unit and for peer navigation) to inform site-level refinements and processes that may influence primary and

secondary outcomes, and (c) we will use the findings in the data analytical plan to understand what influence implementation factors may have had on the primary and secondary outcomes.

Data sources.

Data for implementation evaluation will be collected at each study site over the course of the study, and reviewed monthly by the implementation evaluation team as part of an iterative analysis process. Data will be drawn from three primary sources:

1. Protocol activities involved in the conduct of the study (i.e. routine meetings, fidelity monitoring, and efficacy evaluation procedures). These data will be routinely collected by study staff involved with documenting (i) the process and outcomes of study-related meetings, (ii) capturing participant data, and (iii) documenting the conduct of the study.
    - A standing agenda item or specific prompts related to the implementation evaluation will be added to existing community engagement meetings and protocol site and cross-site meetings with study staff. Relevant responses will be flagged in meeting minutes or transcripts and field notes from these meetings for analysis.
    - Participant records and encounter forms, quality assurance and quality control (QA/QC) activities conducted by staff, and standardized checklists for assessing fidelity metrics in clinical encounters with providers and linkage to services by peer navigators will be reviewed and summarized at the site level for analysis.
    - Sociodemographic data (e.g. age, gender, race) on PWID at each site will be obtained from published reports or service records from regularly-available services (i.e. MOUD, ART, PrEP, and harm reduction) and for PWID who are screened and enrolled into the study protocol.

2. In-depth interviews with PWID, as well as providers of integrated health care in the mobile unit and key community stakeholders. Interviews will be conducted by field staff trained in qualitative methods either in-person or over the telephone/internet and will be recorded. We anticipate our qualitative sampling framework (see Table 3) will generate ~39 in-depth interviews per study site (195 interviews total) to be conducted approximately 6 months after the start of the study implementation phase while the study is still in the field.

Table 3- Sampling frame for in-depth qualitative interviews

Source	Selection strategy	Sample size per site
PWID	~10% of PWID enrolled at each study site will be purposefully sampled. Sampling criteria will aim to select PWID enrolled in the study that are demographically representative of the local injection community in terms of age, gender, race, and HIV status.	n=17
Mobile Unit staff	Staff who support integrated healthcare delivered in the mobile unit at each site will be sampled. This includes clinicians delivering MOUD and ARV treatment (n=2), peer navigators (n=3), and frontline staff routinely engaged with PWID recuitment and tracking activities (n=2)	n=7
Community stakeholders	Key community stakeholders will be identified through the study's formative landscape analysis to map community	n=15

	assets and gaps in the pre-impelmentation phase. This process will identify regularly available MOUD, HIV, harm reduction, and primary care serivces at each study site. Through this process we will invite up to 3 stakeholders from each of the following service domains who would be involved in the coordination and future sustainabiliy of integrated health care and peer navigaiton: HIV services (n=3), MOUD services (n=3), harm reduction services (n=3), primary care (n=3), and public health officials (n=3).	

3. Ethnographic observations of the surrounding environment and delivery of integrated healthcare and peer navigation at each study site. These data will be captured by experienced research assistance trained in the conduct of ethnographic observations.
   - Landscape analysis conducted in the pre-implementation phase of the study will document the availability of MOUD, HIV, harm reduction, and primary care services regularly availble at local clinics at each study site, how these services were impacted by COVID-19, as well as structural determinants of existing service delivery (e.g. policy regulating the distribution of clean needles, public health infrastructure).
   - Ecological assessment check lists and field notes of “hot spots” in the community will be collected during the pre-implementation phase of the study and used to determine where integrated services should be delivered via mobile unit at each site. These assessments will be routinely conducted throughout the implemenation phase to ensure participant recuitment and intervention delivery occur in the local areas with the greatest need.
   - Qualitative ethnographic observations (3-5 per site) will be conducted at each study site during the implemenation phase at two timepoints. First, at each study site within six months of the intervention implementation, and second at each site at least six months after Week 26, when the initial participants transiton from integrated health care delivered on the mobile unit to community-based services. Field notes and check lists will document contextual factors that: (i) affect protocol implementation and the process through which MOUD, ART, and PrEP services are delivered in the community and in the mobile unit; and (ii) document the patient-provider workflow and the contextual variables (environment, interpersonal) that may affect the process of initiating services in a mobile unit and transitioning to community-based services.
   - Time-and-Motion Studies will be implemented during the ethnographic field observations. They will be conducted over a two-week period at each site during study initiation and again when the intervention is running at full capacity. An experienced research assistant will collect data on the time required to complete each step of the intervention. This work may be completed as part of QA/QC activities, i.e., routine periodic observation of intervention staff to ensure they are implementing the intervention correctly. QA/QC checklists created for this staff evaluation will be used to guide the time-and-motion studies. These data will document the number of staff and number of staff hours involved in delivering the intervention to participants receiving (i) integrated healthcare on the mobile unit, and (ii) transitioning PWID from the mobile unit to community-based care via peer navigation.

Cost-Effectiveness Procedures

No participant-oriented procedures are required to complete the planned cost-effectiveness analyses (CEA). Data sources for cost-effectiveness will include estimates of effectiveness from modeling, and estimates of costs to include (1) costs of all commodities used in the intervention; (2) labor costs for intervention workers; (3) intervention startup costs; (4) average time participants spent with intervention including transportation / staff time / time for referrals; (6)

local wages of target population; (5) rent; (6) maintenance; (7) volunteer activities; (8) user fees; (9) value of donated goods and services; and (10) other relevant costs, including training of providers and mobile unit fuel costs. Cost estimates will also incorporate data from time-and-motion studies as described above in this section, which will be collected during participant interactions with staff, but which will not entail collection of participant-specific data.

6.7 Study Visit Windows

For each required study visit, there is an allowable visit window specifying on which study days (post-enrollment) the visit is "allowed" to be completed. The allowable visit windows are contiguous between week 26 and week 52 and do not overlap. Within each allowable visit window, there is a target visit window and study visits should ideally be conducted within this window. These windows are outlined in Table 4 below. If more than one visit is necessary to complete all visit procedures, these could be completed during multiple days within the allowable visit window.

Table 4: Study Visit Windows

Visit	Target Visit Day	Target Visit Window	Allowable Visit Window
Screening			Up to 30 days before enrollment
Enrollment	Day 0		
Week 26	Day 182	Day 168 - 210	Day 126 - 308
Week 52	Day 365	Day 351 - 393	Day 309 – until the study is closed at the site

7.0 SAFETY MONITORING AND SERIOUS ADVERSE EVENT REPORTING

7.1 Adverse Event Definition and Reporting

In this study, the only drugs that will be dispensed or prescribed will be US FDA-approved medications for treatment or prevention of OUD, HIV, and STIs and prescriptions for contraceptives, primary care concerns, and chronic conditions, if indicated. There are no investigational products in this protocol. Therefore, there will be no monitoring or reporting of unanticipated treatment-related risks with the following exception; site investigators will report to the FDA any serious adverse events that are "unexpected" per the current version of the package insert for drugs provided for MOUD, ART or PrEP via the FDA's MedWatch form, copying the RSC Safety Office.

Adverse events (AEs) that study staff become aware of will be recorded in source documentation and will be assessed for seriousness. Non-serious AEs will not be reported in the study database and referrals for care will be provided as necessary. Serious adverse events (SAEs) are those AEs that result in one or more of the following outcomes:

- Death
- A life-threatening (i.e., an immediate threat to life) event
- Requires in-patient hospitalization or prolongation of an existing hospitalization

- A persistent or significant incapacity or substantial disruption of the ability to conduct normal life functions
- A congenital anomaly/birth defect
- A medically important event that may not be immediately life-threatening or result in death or hospitalization but may jeopardize the patient or may require intervention to prevent one of the other outcomes listed in the definition above

Examples of SAEs that can be expected to occur during this study include deaths (e.g., overdoses or other addiction-related deaths) and life-threatening events requiring intervention (e.g. overdose reversals). Study participants will be provided contact information and instructed to contact the study clinician to report any SAEs they may experience. For life-threatening events, they will also be instructed to seek immediate emergency care.

All SAEs will be entered into the study data base, with appropriate levels of documentation and notification of the IRB and sponsors. For this trial, the intervention is the provision of peer navigation and the approach of providing medical care in a mobile health delivery unit. Investigators will therefore need to assess whether the SAE is related to the participant's receipt of peer navigation services or receipt of medical care in the mobile health delivery unit as opposed to a conventional health care clinic. SAEs are included in reports to the Data and Safety Monitoring Board (DSMB) for review.

There will be no expedited reporting of adverse events to DAIDS. However, it will be important that site teams routinely review clinical events that occur among their participants and alert study leadership if any individual incidents or trends raise concern about conduct of the study at that site or raise the possibility of serious or unanticipated risks to study participants posed by the trial. If such a risk is identified, by either site staff or other study team members, it will be reported and responded to as described below in Section 7~~1~~.6.

7.2 Grading Severity of Events

The Division of AIDS Table for Grading the Severity of Adult and Pediatric Adverse Events, Corrected Version 2.1, July 2017, will be used for determining and reporting the severity of adverse events. The DAIDS grading table is available on the DAIDS Regulatory Support Center (RSC) website at https://rsc.niaid.nih.gov/clinical-research-sites/daids-adverse-event-grading-tables.

7.3 Reporting Timeframe

The reporting period for all participants is from the time of enrollment through when a participant exits the study. However, any SAEs that come to the attention of the investigator after a participant has exited the study, but before the database has been locked, will also be reported in the same manner as when the participant was on study.

7.4 Social Impact Reporting

It is possible that participants' involvement in the study could become known to others, and that a social impact may result (participants could be perceived as being HIV positive or at high risk for HIV infection or could be subject to stigma related to their HIV status or their use of injection drugs). For example, participants could be treated unfairly, or could have problems accessing work or social benefits or being accepted by their families and/or communities. A social impact

that is reported by the participant and judged by the IoR/designee to be serious or unexpected will be reported to the IRB at least annually, or according to the IRB's requirements. Social impacts will be collected and submitted to the study database during regular visits. In the event that a participant reports a social impact, every effort will be made by study staff to provide appropriate responses and counseling to the participant as necessary, and/or referral to appropriate resources for the safety of the participant. Each site will provide such responses and counseling in accordance with standardized guidance in the SSP Manual. While maintaining participant confidentiality, study sites may engage their community advisory board (CAB) in exploring the social context surrounding instances of social impacts, to minimize the potential occurrence of such an impact.

It will be important that site teams routinely review social impacts that occur among their participants and alert the CMC, including the DAIDS Medical Officer, if any individual impacts or trends in impacts raise concern about conduct of the study at that site or raise the possibility of serious or unanticipated risks to study participants posed by the trial. See Section 7.6 below for further information about how possible serious or unanticipated risks will be reported and addressed.

Prior to study initiation, each site will conduct a "landscape analysis" of the local environment, including local laws, policies and practices affecting PWID. As part of this analysis, the site team will determine the degree of risk (of arrest, incarceration, physical harm, unwanted disclosure of drug use, of other harms) facing PWID in that location and whether study participation may put PWID at greater risk for harm. Each site will also enact a strategy to mitigate these risks and build support for the study prior to implementation and during study conduct. A key component of this work will be engagement of local stakeholders such as government officials, law enforcement personnel, public health departments, local medical providers, and community leaders to explain the study and obtain support for the work.

7.5 Other Unanticipated Problems

In addition to SAEs related to the study intervention, social impacts and unanticipated medication-related SAEs, other unanticipated problems may arise in the conduct of this research. An unanticipated problem is any incident, experience, or outcome that meets all of the following criteria:

- Unexpected (in terms of nature, severity, or frequency) given (a) the research procedures that are described in the protocol-related documents, such as the IRB-approved research protocol and informed consent document; and (b) the characteristics of the subject population being studied;
- Related or possibly related to participation in the research (possibly related means there is a reasonable possibility that the incident, experience, or outcome may have been caused by the procedures involved in the research); and
- Suggests that the research places subjects or others at a greater risk of harm (including physical, psychological, economic, or social harm) than was previously known or recognized.

All unanticipated problems that meet the three criteria above will be reported to the IRB and to the CMC as described in Section 7.6 below.

7.6 Safety Monitoring and Clinical Data Review

A multi-tiered safety review process will be followed for the duration of this study. The study site investigators are responsible for the initial identification, evaluation and reporting of safety concerns at the participant or site level. Sites are required to have detailed SOPs describing methods for identifying, reporting and managing serious or unanticipated safety concerns raised by SAEs, social impacts or other unanticipated problems.

When sites identify a potentially serious or unexpected risk or problem, they will report it to the CMC. The CMC will work together with appropriate entities such as the site investigators, LOC CRM, SDMC Biostatistician, SDMC Clinical Coding and Safety Staff, HPTN LC, DAIDS personnel (in addition to the Medical Officer on the CMC) and other study team members as appropriate to evaluate the risk or problem and determine an appropriate response. The timelines and mechanisms for reporting and responding to such events will be described in the SSP.

The study team will use regularly scheduled conference calls during the period of study implementation to address safety concerns or unanticipated problems that have been identified. Additional *ad hoc* calls will be convened if required.

Participant safety data is also monitored by the SDMC Clinical Safety staff, who review incoming safety data for completeness and consistency on an ongoing basis. Events identified as questionable, inconsistent, or unexplained will be queried for verification.

This study also will be monitored by a NIAID DSMB, which will meet at least annually to review safety and efficacy data. More frequent or *ad hoc* reviews of safety data may be conducted by the DSMB as needed. The DSMB may make recommendations based on review of safety and efficacy data.

7.7 Pregnancy

The CMC must be notified of any pregnancies identified at Enrollment or that occur among participants on study.

All pregnancies should be followed until the final outcome can be determined. The appropriate case report form for pregnancy outcome and obstetric medical complications at the end of the pregnancy may be completed, even after the participant is off study, up until the point that the study database is closed.

Participants should be advised that not all contraceptive choices can prevent HIV transmission. Study participants who are sexually active with HIV-positive or unknown HIV serostatus partners should be advised that they need to consider effective strategies for reducing the risk of HIV transmission. Participants should be instructed to discuss contraceptive choices and HIV risk reduction methods with their health care provider.

Pregnancies that occur on study among participants taking ART or PrEP should be reported to The Antiretroviral Pregnancy Registry. More information is available at www.apregistry.com. Phone: 800-258-4263; Fax: 800-800-1052. Intrapartum complications and/or pregnancy outcome will be recorded to The Antiretroviral Pregnancy Registry as well as on study case report forms, if possible.

8.0 STATISTICAL CONSIDERATIONS

8.1 Review of Study Design

This is a two-arm, controlled, individually randomized, open label study that will include HIV-positive and HIV-negative PWID from five major cities in the US. The purpose of this study is to determine whether providing 26 weeks of peer navigation with “one stop” integrated services in a mobile unit – particularly MOUD for OUD and HIV treatment and prevention medications -- to PWID with OUD will improve uptake and adherence to MOUD and uptake and adherence to ART or PrEP, compared to 26 weeks of peer navigation to community-based services. This trial will likely be conducted in the context of a significant COVID-19 epidemic which may reach peak levels in the five study cities at different times and may have varying levels of impact in each city. The individually randomized design used in this study (as compared to a cluster-randomized design) will protect against confounding that could arise if cities in one arm of the trial were by chance more affected by the epidemic than cities in the other arm.

8.2 Endpoints

8.2.1 Primary Endpoints

Consistent with the primary study objective to evaluate whether the intervention improves use of MOUD, increases rates of viral suppression among HIV-positive individuals, and increases use of PrEP among HIV-negative individuals, as measured at 26 weeks, the following endpoints will be assessed:

- Documented current use of MOUD: alive, retained, with biological evidence of MOUD (any detectable medications) at the week 26 visit and a MOUD prescription current at 26 weeks after enrollment
- In the HIV-positive cohort: alive, retained, and virally suppressed (VL<200 copies/mL) at the week 26 visit
- In the HIV-negative cohort: alive, retained, HIV negative, with detectable PrEP drugs in dried blood spot (DBS) samples at the week 26 visit

8.2.2 Secondary Endpoints

Consistent with secondary study objectives (1a-c), to evaluate whether the intervention improves use of MOUD, increases rates of viral suppression among HIV-positive individuals, and increases use of PrEP among HIV-negative individuals, compared to the active control condition, the following endpoint(s) will be assessed:

- Documented current use of MOUD: alive, retained, with biological evidence of MOUD (any detectable medications) at the week 52 visit and a MOUD prescription current at 52 weeks after enrollment
- Documented use of MOUD during the study: a MOUD prescription documented during the 52 weeks of study follow-up
- In the HIV-positive cohort: alive, retained, and virally suppressed (VL <200 copies/mL) at the week 52 visit
- In the HIV-negative cohort: alive, retained, HIV negative, with detectable PrEP drugs in DBS at the week 52 visit
- In HIV-negative cohort: alive, retained, HIV negative, with protective levels of PrEP drugs in DBS samples at the week 26 and 52 visits

Consistent with secondary endpoint (1d) to evaluate whether the intervention reduces opioid and polysubstance use at 26 and 52 weeks, compared to the active control condition, the following endpoint(s) will be assessed:

- Alive, retained, and no opioids (natural or synthetic), stimulants (methamphetamine, cocaine) or benzodiazepines detected in urine samples at the week 26 and 52 visits

Consistent with secondary study objective (1e) to evaluate whether the intervention reduces prevalence of bacterial STIs, compared to the active control condition, the following endpoint(s) will be assessed:

- Alive, retained and no evidence of gonorrhea, chlamydia, or new or recurrent syphilis infection detected at the week 26 and 52 visits

Consistent with secondary study objective (1f) to evaluate whether the intervention reduces the rate of fatal and non-fatal overdose events by 26 and 52 weeks, compared to the active control condition, the following endpoint(s) will be assessed:

- Death, with overdose as cause
- Self-report of non-fatal overdose, collected at week 26 and 52 visits

Consistent with secondary study objective (1g) to assess whether the intervention increases the proportion of participants with undetectable HCV RNA among those with chronic HCV infection at enrollment, compared to the active control condition, the following endpoint(s) will be assessed:

- Undetectable HCV RNA at the week 26 and 52 visits among participants with chronic HCV at enrollment

Consistent with secondary study objective (1h) to evaluate whether the intervention reduces HCV incidence, compared to the active control condition, the following endpoint(s) will be assessed:

- HCV antibody positive at the week 52 visit among participants who are HCV antibody negative at enrollment

Consistent with secondary study objective (2) to evaluate whether 26 weeks of "one stop" integrated health services delivered in a mobile health delivery unit, supported by peer navigation, increases MOUD use, viral suppression, and PrEP use at 26 and 52 weeks compared to enrollment, the following endpoint(s) will be assessed:

- In the intervention arm, change over time in the use of MOUD during the study, comparing documented use of MOUD (biological evidence of MOUD - any detectable medications - and a current MOUD prescription) at 26 and 52 weeks to documented use of MOUD at enrollment. MOUD use is assumed to be zero at enrollment due to study exclusion criteria. Follow-up endpoints will be defined as alive, retained, and having documented MOUD use.
- In the intervention arm of the HIV-positive cohort: change over time in the proportion of people with viral suppression (VL<200 copies/mL), comparing 26 and 52 weeks to enrollment. Follow-up endpoints will be defined as alive, retained, and virally suppressed.
- In the intervention arm of the HIV-negative cohort: change over time in the proportion of people with detectable PrEP drugs in DBS at 26 and 52 weeks compared to enrollment. Follow-up endpoints will be defined as alive, retained, and having detectable PrEP.

Consistent with secondary study objective (3) to evaluate whether 26 weeks of peer navigation to similar health services available at community-based agencies increases MOUD use, viral

suppression, and PrEP use at 26 and 52 weeks compared to enrollment, the following endpoint(s) will be assessed:

- In the active control arm, change over time in the use of MOUD during the study, comparing documented use of MOUD (biological evidence of MOUD - any detectable medications - and a current MOUD prescription) at 26 and 52 weeks to documented MOUD use at enrollment. MOUD use is assumed to be zero at enrollment due to study exclusion criteria. Follow-up endpoints will be defined as alive, retained, and having documented MOUD use.
- In the active control arm of the HIV-positive cohort: change over time in the proportion of people with viral suppression (VL<200 copies/mL), comparing 26 and 52 weeks to enrollment. Follow-up endpoints will be defined as alive, retained, and virally suppressed.
- In the active control arm of the HIV-negative cohort: change over time in the proportion of people with detectable PrEP drugs in DBS at 26 and 52 weeks compared to enrollment. Follow-up endpoints will be defined as alive, retained, and having detectable PrEP.

Consistent with secondary study objective (4) to assess the prevalence of SARS-CoV- 2 seropositivity at baseline, 26 and 52 weeks, the following endpoint will be assessed:

- Laboratory evidence of antibodies to SARS-CoV-2

Consistent with secondary study objective (5) to document the impact of the COVID-19 epidemic on participants' experiences of seeking, obtaining and/or maintaining health services, housing, food security and drugs, the team will:

- Document self-reported subjective experiences linked to COVID-19 when seeking MOUD, HIV care (ART, PrEP), STI testing and treatment, hepatitis screening and treatment, primary care, and harm reduction counseling.

Consistent with implementation objectives to evaluate implementation of "one-stop" integrated health services using a mobile unit, supported by peer navigation, across study sites to identify mechanisms at multiple levels to:

(6a) Guide real-time improvements and refinement in the conduct of the study to ensure primary and secondary outcomes are met with fidelity, the team will:

- Describe the local opioid and HIV epidemics and contextual facilitators and barriers that influenced the delivery of integrated services at each study site, and site-specific refinements used to maintain fidelity to service delivery.

(6b) Examine the quality and process of services delivered in each study arm, particularly as these affect primary and secondary outcomes, the team will:

- Describe contextual factors that may have influenced the quality and process of delivering services in each study arm, including the initiation into and transition out of mobile service delivery.

(6c) Develop evidence-based guidance for policymakers on the uptake and implementation of integrated health services using peer navigation and mobile health units in urban US regions to address HIV in PWID, the team will:

- Describe policies and other external environmental factors that affected the uptake and implementation of integrated health services and peer navigation via mobile unit at each study site; informing how these services may be best implemented in other settings.

(6d) identify factors that enhance or impede the delivery of integrated health services using a mobile unit, supported by peer navigation, on primary and secondary outcomes, including responding to the impact of COVID-19 on service delivery, the team will:

- Characterize outcomes of implementing integrated health services via mobile unit across study sties that may affect the impact of the intervention arm on co-occurring treatment outcomes (MOUD, ART, PrEP) and how service delivery and outcomes may be impacted by COVID-19 outbreaks.

Consistent with the secondary study objective (7a) to use mathematical modeling to estimate the effect of integrated health services delivered in a mobile unit, supported by peer navigation, on reducing HIV incidence in PWID and their sexual and injection partners, the following endpoints will be assessed:

- Cumulative HIV incidence over one year for HIV-negative PWID with OUD in the intervention arm and the active control arm. This will be compared to HIV incidence under SOC informed by background markers via NHBS and other datasets where available.
- Cumulative HIV transmission over one year from the cohorts of HIV-positive PWID with OUD in intervention arm and active control arm. This will be compared to estimated HIV transmission under SOC informed by background markers via NHBS and other datasets where available.
- Number of HIV-related deaths averted in the intervention arm and the active control arm in comparison to the background HIV-related deaths from county death indices at baseline through week 52.
- Changes in hepatitis and STI acquisition for cohorts of PWID with OUD in the intervention arm and active control arm in comparison to the background rates of hepatitis and STI acquisition taken from departments of public health records at baseline through week 52
- Quality-adjusted life years (QALYs) gained with the integrated strategy including non-HIV benefits such as treatment and prevention of STIs, HAV, HBV, HCV, improvements in health, employment, overdose and criminal justice factors linked with improved retention in MOUD.

Consistent with the secondary study objective (7b) to use mathematical modeling to estimate the cost-effectiveness of integrated health services provided in a mobile unit and supported by peer navigation, the following endpoints will be assessed:

- Estimated per-person incremental costs to provide the integrated strategy (intervention arm) and the limited intervention (active control arm) compared to SOC
- Estimated treatment costs incurred (and averted, by preventing HIV) due to the integrated strategy and the limited intervention
- Incremental cost effectiveness ratio (ICER) estimate
  - per incident HIV case
  - per HIV-associated death, and
  - per QALY gained comparing the intervention arm to the active control arm

8.2.3 Process Endpoints

Consistent with process objective (1) to assess the time to provide MOUD treatment, ART and PrEP in the intervention arm, the following endpoints will be assessed in the intervention arm:

- Proportion of participants for whom MOUD was provided within 1 week of enrollment
- Proportion of HIV-positive participants who were ART naïve or not currently on ART provided ART within 2 weeks of enrollment
- Proportion of HIV-negative participants who were provided with PrEP within 2 weeks of enrollment

Consistent with process objective (2) to assess the proportion of participants in the intervention arm linked to community-based MOUD, ART and PrEP services at 26 weeks, the following endpoints will be assessed in the intervention arm:

- Proportion of participants linked to MOUD at community-based services by the week 26 visit
- Proportion of HIV-positive participants linked to care for HIV at community-based services by the week 26 visit
- Proportion of HIV-negative participants linked to care for PrEP at community-based services by the week 26 visit

8.3 Sample Size

Sample sizes are chosen to provide sufficient power for the individual (HIV-related and MOUD-related) primary outcomes. By computing sample sizes based on each individual outcome separately and choosing the largest sample size needed for any outcome, high power is achieved for all individual outcomes. Success rates for HIV-related outcomes are expected to be different for HIV-positive and HIV-negative participants, and hence sample sizes are computed separately for the two cohorts. Prior work in PWID populations suggests that uptake of MOUD, ART, and PrEP in the control condition will likely be modest;[39,40] we anticipate 25% uptake of MOUD , 42.5% achieving viral suppression, and 5% uptake of PrEP in the control arm. All sample sizes are computed to provide 90% power to detect a fifteen percentage point difference in the proportion of participants achieving success, assuming a 5% type-I error rate (two-sided). A sample of 400 participants is needed to detect a 15-point difference in MOUD use (25% vs. 40%) between the active control and intervention arm at 26 weeks. A sample of 460 people is needed to detect a 15-point difference in viral-load suppression between the study arms (42.5% vs. 57.5%) among HIV-positive participants. For the HIV-negative cohort, 216 participants would be needed see a 15-point difference in PrEP uptake (5% vs. 20%). To cover both HIV and MOUD outcomes, the HIV-negative cohort will need 400 participants, and the HIV-positive cohort will need 460 participants, leading to a total sample size of 860 people.

8.4 Accrual and Retention

The study cohort (n=860) will be enrolled over the course of approximately 2.5 years and followed for 52 weeks each. This corresponds to a rate of approximately 50 new enrollees per month, or, approximately 12-13 new enrollees per month at each of the five sites.

8.5 Random Assignment/Study Arm Assignment

Random treatment-arm allocation will occur at a 1:1 ratio, stratified by study site and by HIV status cohort. Permuted-block randomization will be used to assure balanced groups within each study site and HIV-status cohort.

8.6 Blinding

This study is unblinded.

8.7 Data and Safety Monitoring Oversight

NIAID DSMB oversight is planned for this study. The DSMB will conduct interim reviews of study progress, including rates of participant accrual, visit retention, completion of primary and secondary endpoint collection, and, in a closed report, safety data by arm. The frequency and

content of DSMB reviews will be determined prior to the start of the study and outlined in the SSP Manual.

8.8 Statistical Analysis

This section briefly describes the final study analyses. Detailed technical specifications of the statistical analyses will be described in a separate Statistical Analysis Plan.

8.8.1 Primary Analyses

All primary endpoint proportions will be compared across study arms using simple linear-binomial regression models (estimating risk differences) adjusted for site (strata variable), with separate models for the HIV-positive and HIV-negative cohorts. Planned sample sizes should allow for Normal-based confidence intervals. No interim analyses are planned.

8.8.2 Secondary Analyses

Secondary analysis endpoint proportions will be compared using the identical method described for the comparison of proportions among primary endpoints with the exception of within-cohort change over time. Within-cohort change over time will be estimated using correlated-data binomial regression (a random-effects model estimating risk differences) adjusted for city. Incidence rates will be computed as the number of incident events (including recurrent events where applicable) divided by total number of accrued person-years in each study arm, where person time is computed for each person as the difference between enrollment date and the last available sample-collection date for which the corresponding STI test was performed. Person-time for participants with new HIV infection will be calculated as the difference between enrollment date and date of first detection of HIV infection.

Implementation Evaluation Analysis
The analysis for each objective will be iterative, whereby the implementaiton team meets monthly to review the incoming data for ideas, themes, and patterns that emerge from the data pertaining to the PRISM framework's multi-level factors that influence the implementation process that will be used to define a set of analytic coding categories. Trained staff will iteratively code the qualitative data and employ qualitative analysis techniques. Quantitative data will be descriptively summarized. The implementation evaluation analysis for each implementation objective will be achieved via parallel mixed method data analysis, integrating qualitative and quantative results to triangulate and interpret overall findings.

Results from pre-implementation analyses will inform the implementation of the intervention across study sites; implementation results will be used to systematically document site-specific refinements and document fidelity to the "one-stop" integrated strategies delivered in the mobile unit and supported by peer navigation. Ultimately, it is hoped that results from the analysis phase can inform implementation of the study's integrated healthcare services approach as a public health program to benefit PWID and to reduce drug-associated HIV transmission in cities across the US.

Modeling and Cost-Effectiveness Analysis
The modelling analysis will be conducted in two distinct phases. First, a stochastic individual-based model will be developed to simulate HIV acquisition among HIV-negative participants and

HIV transmission from HIV-positive participants for cohorts of PWID with OUD representative of the trial populations in the settings where HPTN 094 is conducted. This model will be used to help interpret the trial results by providing an estimate of the intervention impact on the HIV acquisition risk among HIV-negative trial participants and HIV transmission rate from HIV-positive trial participants. Second, a cost-effectiveness analysis of the integrated intervention will be performed that will take into account the costs of the full (integrated strategy) and limited (active control arm) interventions, the treatment costs incurred (and averted) in preventing new HIV infections, and modeled effectiveness. Effectiveness and cost-effectiveness results will be reported for the intervention compared to SOC.

9.0 HUMAN SUBJECTS CONSIDERATIONS

9.1 Ethical Review

This protocol and the sample informed consent forms (ICFs) contained in Appendices II and III will be reviewed and approved by the HPTN Scientific Review Committee and NIAID Prevention Science Review Committee with respect to scientific content and compliance with applicable research and human subjects regulations.

The protocol, site-specific ICFs, participant education and recruitment materials, and other requested documents — and any subsequent modifications — also will be reviewed and approved by the IRB responsible for oversight of this research study.

Subsequent to initial review and approval, the IRB will review the protocol at least annually. The investigators will make safety and progress reports to the IRB at least annually, and within three months of study termination or completion. These reports will include the total number of participants enrolled in the study, the number of participants who completed the study, all changes in the research activity, and all unanticipated problems involving risks to human subjects or others. In addition, all open DSMB reports will be provided to the IRB. Study sites will submit documentation of continuing review to the DAIDS Protocol Registration Office, in accordance with the current DAIDS Protocol Registration Policy and Procedure Manual.

9.2 Informed Consent

Written informed consent will be obtained from each study participant. Each study site is responsible for developing a study ICF for local use, based on the templates in Appendices II and III, which describe the purpose of the study, the procedures to be followed, and the risks and benefits of participation, in accordance with all applicable regulations. If applicable, the study site also is responsible for translating the template form into languages other than English and verifying the accuracy of the translation by performing an independent back-translation. Sites may have additional consent requirements based upon local requirements, e,g., for clinical care, privacy protection, etc.

Literate participants will document their provision of informed consent by signing their informed consent forms. Non-literate participants will be asked to document their informed consent by marking their ICFs (e.g., with an X, thumbprint, or other mark) in the presence of a literate third-party witness. (Further details regarding DAIDS requirements for documenting the informed consent process with both literate and non-literate participants are provided in the DAIDS Standard Operating Procedure for Source Documentation.) Any other IRB requirements for obtaining informed consent from non-literate persons also will be followed.

Participants will be provided with copies of their ICFs if they are willing to receive them.

9.3 Risks

Taking blood samples may cause participants some pain, bruise their arm, or cause them to feel lightheaded. In rare cases they may faint. There is a slight chance of infection when blood is drawn. Participants may experience pain or discomfort in their throat, rectum or vagina from the swab. In some cases, they may have some bleeding.

We will make every effort to protect participants' confidentiality during the study, however, it is possible that others may learn of a person's participation in this study and may think that the participant is living with HIV or at high risk for acquiring HIV or may make assumptions about the participant's use of drugs. The participant could face stigma related to HIV or drug use. Participants may be nervous while waiting for HIV or other test results. If the tests show that a participant has HIV or another infection, they may worry about their health and future. The questions we will ask participants about sexual behavior or drug use may make them feel uneasy. Trained counselors will be available to help participants deal with these feelings.

There are no risks related to investigational products in this study because no investigational products will be used.

For participants assigned to the group that WILL NOT receive medical care in the mobile health delivery unit, medications to treat STIs will be provided by the study staff at the Enrollment Visit, if they have STI symptoms, and they will be offered naloxone kits during study participation that can be used to treat an overdose. Participants may also be given or prescribed other commonly-used medications at the Enrollment Visit to treat things like a cough or infection. The clinician will explain to the participant any side effects associated with any of those medications. After the Enrollment Visit, the peer navigator will help the participant receive care from community-based services.

Participants assigned to the group that WILL receive medical care in the mobile unit will receive or be prescribed medications by the study staff during the first six months of their participation in the study. The medications that a participant might be dispensed in the mobile unit include those to treat or prevent: STIs or other bacterial infections, HIV, opioid use disorder and overdose. Participants may also be prescribed other medications as part of primary care provided in the mobile health delivery unit. The clinician will explain to the participant any side effects of the specific medications they are prescribed or given.

9.4 Benefits

Participants in this study will receive many health services at no charge. Participants will be tested for HIV, HAV, HBV, HCV, and other STIs. Participants will receive treatment or referral for treatment, as appropriate, for these infections. The counseling participants receive during this study may help them avoid HIV, HAV, HBV, HCV, and other STIs. For participants with HIV, counseling may help them learn how to better care for themselves and avoid passing HIV to sexual partners (or their fetus, if pregnant). HIV-negative participants will be offered PrEP or referral for PrEP to help avoid HIV infection. Peer navigators will work to link participants to health services, including treatment for OUD and HIV care or prevention services. Participants will receive free condoms and naloxone kits for overdose reversal. Participants in the group that

receives medical services in the mobile unit will be provided with medical care and medication in a single convenient location.

Participants may receive indirect benefits from this study. The information gathered during this study may show how medical services can be provided more successfully to people who inject drugs, particularly services for OUD and HIV. This could influence how medical services are provided in the future and may be beneficial to participants and their community.

9.5 Population-Specific Considerations

As noted in Section 1.1, persons with OUD and living with or at risk for HIV in the US typically face multiple and overlapping problems that can include criminal justice involvement. In HPTN 094, prisoners will not be recruited or enrolled from jail or prison settings (persons with parole status will be recruited and enrolled), nor will study-related visits with study participants be conducted in prisons or jails. Participants who become incarcerated during their time on study will be scheduled for study activities for which they are eligible upon release.

As noted in Sections 1.2 and 4.3 of this protocol, approximately 25% of the participants in this study will be women. It is expected that some number of these participants will be or will become pregnant while enrolled in the study. The "Common Rule" for conduct of human research studies funded by the US Government stipulates additional consideration for pregnant women as subjects, particularly regarding benefits and risks to both the pregnant woman and the fetus.

In considering the risks and benefits presented by the participation of pregnant women in this study, the intervention being investigated is the *combination* of *standard of care* therapies for people with OUD and people with (or at risk for) HIV, in a mobile health delivery unit, with peer navigation. The medications that will be prescribed and provided are not investigational but will be US FDA-approved medicines used for their approved indication. Providing these standard therapies, in a manner that we expect will improve their uptake and adherence (i.e., in an accessible mobile health delivery unit, with supportive navigation), will not increase risks to either a pregnant woman or to her fetus greater than would be encountered if she were to obtain them in a standard clinic setting.

The therapies that are the focus of this intervention are all commonly prescribed for pregnant women because of their acknowledged beneficial risk-benefit profile for both the woman and the fetus. For ART, treatment in a pregnant woman is highly effective at preventing transmission of HIV to the fetus and treatment has health benefits for the mother (current guidelines recommend treatment for all HIV-positive adults for their own health), with very low risk presented to either woman or fetus by currently used regimens. For PrEP, both the woman and the fetus benefit from avoiding potential HIV infection, and the currently approved PrEP regimen (Truvada) has a very well-studied and minimal risk profile. For MOUD, there are substantial benefits to treatment for both the woman and the fetus compared with the harms from untreated opioid use disorder. A woman on treatment spares herself and her developing fetus the fluctuating levels of opioid exposure and injection-related risks that occur from cycles of use and withdrawal of street-acquired opioids. MOUD reduces the dysregulated behavior of addiction, allowing the woman to improve nutrition, sleep, situational stability, attend pre-natal care visits, etc., with corresponding benefits for her fetus. Recommendations for treatment will always be based on the clinician's assessment of the individual participant's needs; if a particular therapy (i.e., methadone, buprenorphine, naltrexone) is deemed not appropriate for a particular participant (pregnant or otherwise), it will not be prescribed.

In the risk-to-benefit analysis, benefits of offering this intervention to pregnant women far outweigh potential risks for both the woman and her fetus. Excluding pregnant women would present ethical challenges, principal among them violating the principle of beneficence by excluding the opportunity for pregnant participants to benefit from the interventions provided (including medications) in this study in the face of no more than minimal risks to pregnant women and their fetuses treated using these medications. As well, including pregnant women allows the study population to be more representative of the population of PWID with OUD, and therefore allows our findings to be more generalizable.

9.6 Incentives

Pending IRB approval, participants will be compensated for their time and effort in this study, and/or be reimbursed for travel to study visits and time away from work. As well, some participants may be offered additional compensation for referring other PWID who enroll in the study. Site-specific reimbursement amounts will be specified in the study ICFs.

9.7 Confidentiality

All study-related information will be stored securely. All participant information will be stored in locked file cabinets in areas with access limited to study staff. All laboratory specimens, reports, study data collection, process, and administrative forms will be identified by a coded number only to maintain participant confidentiality. All databases will be secured with password-protected access systems. Forms, lists, logbooks, appointment books, and any other listings that link participant ID numbers to other identifying information will be stored in a separate, locked file in an area with limited access.

Interviews will be transcribed by qualified personnel and all identifiable information will be removed from the transcripts. All reports and publications will be carefully redacted to ensure that identification of interview or focus group participants is not possible.

Participants' study information will not be released without the written permission of the participant, except as necessary for monitoring by the NIAID, National Institute on Drug Abuse (NIDA) and/or their contractors; representatives of the HPTN LOC, SDMC, and/or LC; other government and regulatory authorities, and/or IRB.

The HPTN will obtain a Certificate of Confidentiality from the US Department of Health and Human Services that will be applicable for this study. Sites may register under the Certificate through the HPTN LOC once they have obtained IRB approval for the study. This Certificate protects study staff from being compelled to disclose study-related information by any Federal, State or local civil, criminal, administrative, legislative, or other body.

9.8 Communicable Disease Reporting Requirements

Study staff will comply with all applicable local requirements to report communicable diseases identified among study participants to local health authorities. Participants will be made aware of all reporting requirements during the study informed consent process.

9.9 Study Discontinuation

The study also may be discontinued at any time by NIDA, NIAID, the HPTN, other government or regulatory authorities, and/or the IRB.

10.0 LABORATORY SPECIMENS AND BIOHAZARD CONTAINMENT

Laboratory procedures are described below, Appendices (IA and IB), and Section 6.0; additional laboratory procedures are required at a subsequent visit for participants who have a reactive or positive HIV test result after enrollment (See Appendix IC).

10.1 Local Laboratory Specimens

As described in Section 6.0, the following types of specimens will be collected:

- Blood
- Urine
- Swabs (oral, rectal, vaginal)

As described in Section 6.0, the following types of testing will be performed in the mobile unit or at the local laboratory:

- HIV testing – see SSP Manual
- CD4 cell count and HIV viral load testing (if HIV positive)
- Hepatitis testing, including HBsAg, HBsAb, HBcAb, HCV Ab, HAV IgG, HCV RNA (if HCV positive), and HBV DNA (if needed for clinical management
- Syphilis testing and GC/CT by NAAT: oral swab and rectal swab (all), vaginal swab (women only), urine (men only).
- Substance use testing by urine dip stick (must include opioid, cocaine, amphetamines, benzodiazepines)
- MOUD testing by urine dip stick (must include buprenorphine and methadone)
- Urine pregnancy testing
- Chemistry testing for creatinine, ALT, AST, total bilirubin
- Hemoglobin
- HIV drug resistance testing, if needed for clinical management

Notes: Some tests will be performed in mobile units and some will be performed in local laboratories (see SSP Manual). Other testing related to HIV and HCV infection may be performed for clinical management (see Appendix 1A and 1B).

Each study site (mobile health delivery unit, clinic, laboratory, etc.) must adhere to standards of Good Clinical Laboratory Practice (GCLP) (https://www.niaid.nih.gov/sites/default/files/gclp.pdf) referenced in the HPTN Manual of Operations (MOP), the SSP Manual and local SOPs for proper collection, processing, labeling, transport, and storage of specimens to the local laboratory. In addition, each study site must adhere to the Requirements for DAIDS Funded and or Sponsored Laboratories in Clinical Trials Policy (https://www.niaid.nih.gov/sites/default/files/Lab-Appendix-I-US-Labs.pdf). Specimen collection, testing, and storage at the local laboratory will be documented using the HPTN Laboratory Data Management System (LDMS) as described in the SSP Manual.

10.2 HPTN Laboratory Center Specimens

The following types of specimens will be collected/prepared for testing at the HPTN LC.

- Plasma
- Serum
- Urine
- DBS

10.2.1 Stored Specimens

Plasma, serum, urine, and DBS specimens will be stored at the local site throughout the study. Study specimens will be maintained at the site until at least one year after publication of the primary findings of the study and all protocol-related testing has been performed. A subset of the stored samples will be shipped to the HPTN LC (located in the US) for QA and other assessments. Stored samples may be used to evaluate the performance of laboratory methods relevant to protocol objectives. As indicated below, testing on stored samples will be performed by the HPTN LC or another laboratory selected by the HPTN LC. These samples may not be used for local laboratory testing without written approval by the HPTN LC prior to use.

10.2.2 Virology

For participants with HIV infection, HPTN LC testing will include HIV viral load, and analysis of viral subtypes/strains, drug resistance, and duration of infection; other tests may be performed to characterize the host response to HIV infection. Results will not be returned to the sites or study participants, with the exception of HIV testing (if results obtained at the HPTN LC do not agree with site results).

For participants with HCV infection, HPTN LC testing may include HCV viral load, and analysis of HCV strains, drug resistance, and duration of infection; other tests may be performed to characterize the host response to HCV infection. Results will not be returned to the sites or study participants.

HIV resistance testing will be performed at the HPTN LC or a laboratory designated by the HPTN LC. This testing will be performed retrospectively at the end of the study. Results of this testing will not be returned to study sites. Because real-time resistance testing may be needed for clinical management in the event of HIV infection, each site will have an SOP as to how they will accomplish real-time local or regional resistance testing to assist with clinical decision making; separate specimens should be collected for that testing.

Phylogenetic analysis will also be performed to evaluate the genetic relationships between HIV strains. This will include analysis of clustered/linked HIV infections, transmission dynamics, and the association of behavioral, demographic, and clinical factors with HIV transmission/acquisition. This analysis may also include evaluation of the multiplicity of infection and HIV superinfection. Similar analysis may also be performed to characterize HCV infections and the relationship between HIV and HCV infections.

Laboratory testing will be performed to assess the prevalence of SARS-CoV-2 seropositivity. Testing may also be performed to characterize the host response to infection and factors associated with SARS-CoV-2 seropositivity.

10.2.3 Pharmacology and Toxicology Testing

Plasma, urine, and DBS samples will be processed and frozen locally for subsequent shipment to the HPTN LC following procedures outlined in the SSP Manual.

Plasma and DBS samples for Pharmacology testing will be collected throughout the study for analysis of PrEP use and adherence. Analysis at the HPTN LC may be limited to a subset of the samples. Pharmacology testing will be performed at the HPTN LC or at an outside laboratory designated by the HPTN LC. The primary pharmacologic assessments will be performed using assays that have been validated and approved by the Clinical Pharmacology Quality Assurance (CPQA) Committee. Results will not be returned to the study participants.

Stored plasma may also be tested for the presence of other drugs, including ARV drugs used for HIV treatment or other reasons and drugs used for hepatitis treatment. Stored urine may be tested for the presence of medications used to treat substance use, other concomitant medications, and substances of abuse.

10.3 Quality Control and Quality Assurance Procedures

The clinical sites will document that their clinical laboratories (including clinic laboratories, mobile units, or other locations where processing and testing are performed) have either a Clinical Laboratory Improvement Amendments of 1988 (CLIA)-waiver or are CLIA-certified under the Clinical Laboratory Improvement Amendments of 1988 (CLIA-certified) and/or participate in DAIDS sponsored external quality assurance programs. Laboratories must also follow the DAIDS requirements (link to policy on DAIDS website https://www.niaid.nih.gov/research/daids-clinical-research-laboratory-specimens-management).

LC staff will conduct periodic visits to each site to review the implementation of on-site laboratory QC procedures, including proper maintenance of laboratory equipment and use of appropriate supplies and reagents. LC staff will follow up directly with site staff to resolve any QC or QA problems identified through proficiency testing or on-site visit reviews. Throughout the course of the study, the HPTN LC will select a random sample of stored specimens to test for QA purposes. LC staff will follow-up directly with site staff to resolve any QA problems identified through this process.

HIV diagnostic tests will be listed on the US lab information sheet and will be subject to review and acceptance by DAIDS and the HPTN LC.

Throughout the course of the study, the SDMC will work with the HPTN LC to determine and select random sample of stored specimens to test for QA purposes, and to assist with and/or confirm HIV seroconversion cases.

The SDMC and/or the LC will inform site staff of the samples selected for QA testing, and site staff will ship the selected specimens to the LC. The LC will perform HIV diagnostic testing for QC, endpoint confirmation, end of study confirmation, and will work with the SDMC to compare the results of their tests with the results obtained by the local labs. LC staff will follow-up directly with site staff to resolve any QA problems identified through this process.

10.4 QC for HIV Diagnostic Testing

The local laboratories will perform testing for HIV diagnosis at Screening, Enrollment, and other scheduled visits as described in the SSP Manual. HIV infection status will be confirmed at study sites using local HIV testing guidelines or following the advice from the CMC/HPTN LC. In addition, if a participant has signs or symptoms consistent with acute HIV infection, or expresses a concern about recent HIV acquisition, testing will be performed using an RNA test such as the Aptima HIV-1 Qualitative Assay. Regardless of whether HIV RNA testing is used for diagnostic testing, incident HIV infection (seroconversion events) must be confirmed in all cases using two independent samples collected on different days. HIV algorithms for HIV diagnostic testing are provided in the SSP Manual. The HPTN LC will perform QC testing to confirm seroconversion events and evaluate the accuracy of site test results.

10.5 HIV RNA Monitoring

Quantitative HIV RNA (viral load) testing will be performed at local clinical laboratories to monitor HIV infection in any subject with confirmed HIV infection. Viral load testing will be performed in HIV-positive participants at the visit when HIV infection is confirmed, and at subsequent study visits. Note that this is distinct from use of qualitative HIV RNA testing that is performed to determine HIV infection status.

10.6 CD4 Cell Count Determination

CD4 cell count testing will be performed for participants who are HIV positive at enrollment and for participants who become HIV positive during the study.

10.7 Specimen Storage and Possible Future Research Testing

Study site staff will store urine, plasma, serum, and DBS collected in this study at least through the end of the study (this includes completion of all protocol-related testing, including testing at the HPTN LC). Sample destruction requires approval in writing from the LC to the site and will not occur until at least one year has passed after publication of the primary manuscript. In addition, study participants will be asked to provide written informed consent for their specimens to be stored after testing for all protocol-related testing has been completed (including testing for primary, secondary, and exploratory objectives) for possible future testing. The specimens of participants who do not consent to long-term storage and additional testing will be destroyed at the end of the study after the protocol-related testing has been completed, at least one year after completion of the primary manuscript. A list of samples to be destroyed will be provided by the SDMC, to be reviewed and reconciled by the site. Final approval for sample destruction will be provided in writing to the site by the LC.

10.8 Biohazard Containment

As the transmission of HIV and other blood-borne pathogens can occur through contact with contaminated needles, blood, and blood products, appropriate blood and secretion precautions will be employed by all personnel in the drawing of blood and shipping and handling of all specimens for this study, as currently recommended by the US CDC. All specimens will be shipped using packaging that meets requirements specified by the International Air Transport Association Dangerous Goods Regulations for UN 3373, Biological Substance, Category B, and Packing Instruction 650.

11.0 ADMINISTRATIVE PROCEDURES

11.1 Protocol Registration

Initial Registration of the protocol by the DAIDS Protocol Registration Office (PRO) is required prior to implementation of this protocol. As part of this process, each site must have the protocol and protocol ICF(s) approved, as appropriate, by the IRB. Upon receiving final approval, sites will submit all required protocol registration documents to the DAIDS PRO at the RSC. The DAIDS PRO will review the submitted protocol registration packet to ensure that all of the required documents have been received. In the case of Initial Registration, site-specific ICFs will be reviewed and approved by the DAIDS PRO. Sites will receive an Initial Registration Notification from the DAIDS PRO that indicates successful completion of the protocol registration process. A copy of the Initial Registration Notification should be retained in the site's regulatory files.

Upon receiving final IRB/EC and any other applicable RE approval(s) for an amendment, sites should implement the amendment immediately. Sites are required to submit an amendment registration packet to the DAIDS PRO at the RSC. The DAIDS PRO will review the submitted protocol registration packet to ensure that all the required documents have been received. Site-specific ICF(s) WILL NOT be reviewed and approved by the DAIDS PRO and sites will receive an Amendment Registration Notification when the DAIDS PRO receives a complete registration packet. A copy of the Amendment Registration Notification should be retained in the site's regulatory files.

For additional information on the protocol registration process and specific documents required for initial and amendment registrations, refer to the current version of the DAIDS Protocol Registration Manual, which can be found at https://rsc.niaid.nih.gov/clinical-research-sites/daids-protocol-registration-policy-and-procedures-manual.

11.2 Study Activation

Pending successful protocol registration and submission of all required documents, the HPTN LOC staff will "activate" a site to begin study operations. Study implementation may not be initiated until a study activation notice is provided to the site by the HPTN LOC. In addition, if study activation is determined to be necessary for any subsequent amendments, study implementation may not be initiated until a study activation notice is provided to the site by the HPTN LOC.

11.3 Study Coordination

Study implementation will be directed by this protocol as well as the SSP Manual. The SSP Manual will contain links to the Requirements for Source Documentation in DAIDS Funded and/or Sponsored Clinical Trials, as well as the DAIDS Toxicity Tables. This manual will outline procedures for conducting study visits; data and forms processing; SAE assessment, management and reporting; dispensing medications and documenting product accountability; and other study operations.

Study CRFs and other study instruments will be developed by the protocol team and HPTN SDMC. Data will be submitted to the HPTN SDMC for cleaning, reporting and analysis. Quality

control data queries will be generated on a routine schedule for verification and resolution by site data management staff.

Close coordination between protocol team members will be necessary to track study progress, respond to queries about proper study implementation, and address other issues in a timely manner. Rates of accrual, adherence, follow-up, and SAE incidence will be monitored closely by the team as well as the HPTN SMC. The CMC will address issues related to study eligibility and SAE management and reporting as needed to assure consistent case management, documentation, and information-sharing across sites.

11.4 Study Monitoring

On-site study monitoring will be performed in accordance with DAIDS policies. Study monitors will visit the site to:

- Verify compliance with human subjects and other research regulations and guidelines;
- Assess adherence to the study protocol, study-specific procedures manual, and local counseling practices; and
- Confirm the quality and accuracy of information collected at the study site and entered into the study database.

Site investigators will allow study monitors to inspect study facilities and documentation (e.g., ICFs, clinic and laboratory records, other source documents, paper or electronic CRFs), as well as observe the performance of study procedures. Investigators also will allow inspection of all study-related documentation by authorized representatives of the HPTN LOC, HPTN SDMC, HPTN LC, NIAID, NIDA, IRB, and US regulatory authorities (Office for Human Research Protections (OHRP) and other regulatory agencies). A site visit log will be maintained at each study site to document all visits.

11.5 Protocol Compliance

The study will be conducted in full compliance with the protocol. The protocol will not be amended without prior written approval by the Protocol Chair and DAIDS Medical Officer. All protocol amendments must be submitted to and approved by the IRB and the RSC prior to implementing the amendment.

11.6 Investigator's Records

The Investigator will maintain, and store in a secure manner, complete, accurate, and current study records throughout the study. Under the US Department of Health and Human Services (DHHS) regulations, the Investigator is required to retain all study records relating to research for at least three years after completion of the research, or longer if needed to comply with local regulations.

Completion of a clinical research study occurs when the following activities have been completed:

- All research-related interventions or interactions with human subjects (e.g. when all subjects are off study);

- All protocol-required data collection of identifiable private information described in the IRB-approved research plan;
- All analysis of identifiable private information described in the IRB-approved research plan;
- Primary analysis of either identifiable private or de-identified information.

Study records include administrative documentation — including protocol registration documents and all reports and correspondence relating to the study — as well as documentation related to each participant screened and/or enrolled in the study — including ICFs, locator forms, CRFs, notations of all contacts with the participant, and all other source documents.

11.7 Use of Information and Publications

Publication of the results of this study will be governed by the HPTN Manual of Operations. Any presentation, abstract, or manuscript will undergo review by the HPTN Manuscript Review Committee and DAIDS, prior to submission.

REFERENCES

1. Hedegaard H, Minino AM, Warner M. Drug overdose deaths in the United States, 1999-2018. 2020.
2. Centers for Disease Control and Prevention (CDC). Drug Overdose Death Data. 2017 (accessed July 4 2018).
3. Nolan M, Tuazon E, Blachman-Forshay J, Paone D. Unintentional Drug Poisoning (Overdose) Deaths in New York City, 2000-2017. *New York City Department of Health and Mental Hygiene: Epi Data Brief (104)* 2018.
4. Nicholson HL, Vincent J. Gender Differences in Prescription Opioid Misuse Among U.S. Black Adults. *Subst Use Misuse* 2019; **54**(4): 639-50.
5. Allen B, Nolan ML, Kunins HV, Paone D. Racial Differences in Opioid Overdose Deaths in New York City, 2017. *JAMA Intern Med* 2019; **179**(4): 576-8.
6. Hedegaard H, Minino AM, Warner M. Drug Overdose Deaths in the United States, 1999-2017. *NCHS Data Brief* 2018; (329): 1-8.
7. Ciccarone D. Fentanyl in the US heroin supply: A rapidly changing risk environment. *International Journal of Drug Policy* 2017; **46**: 107-11.
8. Ciccarone D. The triple wave epidemic: Supply and demand drivers of the US opioid overdose crisis. *Int J Drug Policy* 2019.
9. Volkow N, Frieden T, Hyde P, Cha S. Medication-assisted therapies--tackling the opioid-overdose epidemic. *New England Journal of Medicine* 2014; **370**(22): 2063-6.
10. Kolodny A, Courtwright D, Hwang C, et al. The prescription opioid and heroin crisis: a public health approach to an epidemic of addiction. *Annual Review of Public Health* 2015; **36**: 559-74.
11. Compton W, Jones C, Baldwin G. Relationship between Nonmedical Prescription-Opioid Use and Heroin Use. *New England Journal of Medicine* 2016; **374**: 154-63.
12. Compton W, Volkow N, Throckmorton D, Lurie P. Expanded access to opioid overdose intervention: research, practice, and policy needs. *Annals of Internal Medicine* 2013; **158**(1): 65-6.
13. Strathdee S, Beyrer C. Threading the Needle - How to Stop the HIV Outbreak in Rural Indiana. *New England Journal of Medicine* 2015; **373**(5): 397.
14. Platt L, Easterbrook, P., Gower, E., McDonald, B., Sabin, K., McGowan, C., Yanny, I., Razavi, H., Vickerman, P. Prevalence and burden of HCV co-infection in people living with HIV: a global systematic review and meta-analysis. . *Lancet Infect Diseases* 2016; **16**(7): 797-808.
15. Blanco C, Volkow ND. Management of opioid use disorder in the USA: present status and future directions. *Lancet* 2019; **393**(10182): 1760-72.
16. Peters PJ, Pontones P, Hoover KW, et al. HIV Infection Linked to Injection Use of Oxymorphone in Indiana, 2014–2015. *New England Journal of Medicine* 2016; **375**(3): 229-39.
17. Cranston K, Alpren C, John B, et al. Notes from the Field: HIV Diagnoses Among Persons Who Inject Drugs - Northeastern Massachusetts, 2015-2018. *MMWR Morbidity and mortality weekly report* 2019; **68**(10): 253-4.
18. Golden MR, Lechtenberg R, Glick SN, et al. Outbreak of Human Immunodeficiency Virus Infection Among Heterosexual Persons Who Are Living Homeless and Inject Drugs - Seattle, Washington, 2018. *MMWR Morbidity and mortality weekly report* 2019; **68**(15): 344-9.
19. El-Bassel N, Marotta P, Gilbert L, et al. Integrating treatment for opioid use disorders and HIV services into primary care: Solutions for the 21st Century. In: Crosby R, DiClemente R, eds. Preventing HIV Transmission and Improving HIV Care through the Use of Structural-Level Approaches: Global Insights from Successful Approaches. Cambridge, UK: Oxford University Press; In Press.
20. Williams A, Nunes E, Bisaga A, et al. Developing an opioid use disorder treatment cascade: A review of quality measures. *Journal of Substance Abuse Treatment* 2018; **91**: 57-68.

21. Malta M, Magnanini MMF, Strathdee SA, Bastos FI. Adherence to Antiretroviral Therapy Among HIV-Infected Drug Users: A Meta-Analysis. *AIDS and Behavior* 2010; **14**(4): 731-47.
22. Malta M, Ralil da Costa M, Bastos FI. The Paradigm of Universal Access to HIV-Treatment and Human Rights Violation: How Do We Treat HIV-Positive People Who Use Drugs? *Current HIV/AIDS Reports* 2014; **11**(1): 52-62.
23. Volkow ND, Wargo EM. OVerdose prevention through medical treatment of opioid use disorders. *Annals of Internal Medicine* 2018; **169**(3): 190-2.
24. Shoptaw S, Goodman-Meza D, Landovitz RJ. Collective Call to Action for HIV/AIDS Community-Based Collaborative Science in the Era of COVID-19. *AIDS Behav* 2020.
25. Green TC, Bratberg J, Finnell DS. Opioid use disorder and the COVID 19 pandemic: A call to sustain regulatory easements and further expand access to treatment. *Subst Abus* 2020; **41**(2): 147-9.
26. The National Academies of Sciences Engi (Washington District of Columbia). Opportunities to improve opioid use disorder and infectious disease services : integrating responses to a dual epidemic. Washington: The National Academies Press; 2020.
27. Iyengar S, Kravietz A, Bartholomew TS, Forrest D, Tookes HE. Baseline differences in characteristics and risk behaviors among people who inject drugs by syringe exchange program modality: an analysis of the Miami IDEA syringe exchange. *Harm Reduct J* 2019; **16**(1): 7.
28. Allen ST, Ruiz MS, Jones J. Assessing Syringe Exchange Program Access among Persons Who Inject Drugs (PWID) in the District of Columbia. *J Urban Health* 2016; **93**(1): 131-40.
29. Degenhardt L, Peacock A, Colledge S, et al. Global prevalence of injecting drug use and sociodemographic characteristics and prevalence of HIV, HBV, and HCV in people who inject drugs: a multistage systematic review. *Lancet Glob Health* 2017; **5**(12): e1192-e207.
30. Centers for Disease and Prevention. HIV Infection, Risk, Prevention, and Testing Behaviors among Persons Who Inject Drugs—National HIV Behavioral Surveillance: Injection Drug Use, 20 U.S. Cities, 2015. *HIV Surveillance Special Report 18* 2018.
31. Ait-Daoud N, Blevins D, Khanna S, Sharma S, Holstege CP, Amin P. Women and Addiction: An Update. *Med Clin North Am* 2019; **103**(4): 699-711.
32. Palis H, Marchand K, Guh D, et al. Men's and women's response to treatment and perceptions of outcomes in a randomized controlled trial of injectable opioid assisted treatment for severe opioid use disorder. *Subst Abuse Treat Prev Policy* 2017; **12**(1): 25.
33. Wejnert C, Hess KL, Hall HI, et al. Vital Signs: Trends in HIV Diagnoses, Risk Behaviors, and Prevention Among Persons Who Inject Drugs - United States. *MMWR Morb Mortal Wkly Rep* 2016; **65**(47): 1336-42.
34. Lapham G, Boudreau DM, Johnson EA, et al. Prevalence and treatment of opioid use disorders among primary care patients in six health systems. *Drug Alcohol Depend* 2020; **207**: 107732.
35. Yu SWY, Hill C, Ricks ML, Bennet J, Oriol NE. The scope and impact of mobile health clinics in the United States: a literature review. *Int J Equity Health* 2017; **16**(1): 178.
36. Curran GM, Bauer M, Mittman B, Pyne JM, Stetler C. Effectiveness-implementation hybrid designs: combining elements of clinical effectiveness and implementation research to enhance public health impact. *Med Care* 2012; **50**(3): 217-26.
37. Feldstein AC, Glasgow RE. A Practical, Robust Implementation and Sustainability Model (PRISM) for Integrating Research Findings into Practice. *The Joint Commission Journal on Quality and Patient Safety* 2008; **34**(4): 228-43.
38. Glasgow RE, Vogt TM, Boles SM. Evaluating the public health impact of health promotion interventions: the RE-AIM framework. *Am J Public Health* 1999; **89**(9): 1322-7.
39. Miller WC, Hoffman IF, Hanscom BS, et al. A scalable, integrated intervention to engage people who inject drugs in HIV care and medication-assisted treatment (HPTN 074): a randomised, controlled phase 3 feasibility and efficacy study. *Lancet* 2018; **392**(10149): 747-59.
40. Morgan JR, Schackman BR, Leff JA, Linas BP, Walley AY. Injectable naltrexone, oral naltrexone, and buprenorphine utilization and discontinuation among individuals treated for

opioid use disorder in a United States commercially insured population. *J Subst Abuse Treat* 2018; **85**: 90-6.

APPENDIX IA: SCHEDULE OF STUDY VISITS AND PROCEDURES FOR PARTICIPANTS WHO ARE HIV POSITIVE AT ENROLLMENT

	Screening	Enrollment	Care visit(s)[1]	26 Weeks	52 Weeks
**Administrative and Behavioral Procedures**					
Informed consent	X				
Locator information	X	X	X	X	X
MOUD counseling	X	X	X	X	X
HIV risk reduction counseling and, at screening, test results	X	X	X	X	X
Offer condoms and lubricant	X	X	X	X	X
Provide/facilitate access to harm reduction	X	X	X	X	X
Demographic information	X				
Randomization		X			
Behavioral data		X		X	X
Introduction to peer navigator		X			
Conclusion of peer navigation				X	
**Clinical Evaluations/Procedures**					
Assessment for COVID-19[2]	X	X	X	X	X
Assessment for OUD[3], recent injection drug use (track marks)		X	(X)	X	X
Targeted medical history to include MOUD treatment history, HIV risk behaviors, participation in other research studies	X	X	(X)	X[4]	X[4]
Basic physical exam[5]		X	(X)		
Screen for mental health needs and refer for services as indicated		X	(X)	X	X
Initiate or refer for HIV treatment		(X[6])	(X[6])		
COWS assessment and initiate mobile unit-based MOUD treatment program (intervention arm only)		(X[7])	(X[7])		
Provide clinical management of MOUD and HIV infection, including medication or prescription dispensation, as indicated			X		
HAV vaccination referral			X[8]		
HBV vaccination referral			X[9]		
HBV treatment/treatment referral			X[8,9]		
HCV treatment referral			X[8]		X
Development of a clinical plan		X			
Empiric treatment of STIs (if symptomatic)		(X)	(X)	(X)	(X)

Provide lab-based STI results and, if indicated, treatment (intervention arm) or referral (active control arm)			X[10]		
Provide lab-based STI results, and, if indicated, referral				X[10]	X[10]
Provide clinical assessment and management or referral for other medical conditions			X		
Blood collection	X	X	(X)	X	X
Urine collection	X	X	(X)	X	X
Swabs for STI testing[11]		X	(X)	X	X
**Laboratory Evaluations/Procedures**					
HIV rapid testing	X	X			
Laboratory-based HIV testing (see SSP Manual)	X[12]				
HIV viral load		X	(X)	X	X
CD4 cell count		X	(X)		
MOUD testing (urine dip stick)[13]	X	X		X	X
Substance use testing (urine dip stick)[14]	X	X	(X)	X	X
Pregnancy testing (urine)[15]		X	(X)	(X)	(X)
STI testing (syphilis, GC/CT NAAT)		X	(X)	X	X
HCV Ab testing[16]		X			X
HCV RNA (viral load)[17]		X		X	X
HBV testing (HBsAg, HBsAb, HBcAb)		X			
Other HBV-related testing[18]		(X)			
HAV testing (HAV IgG)		X			
Heme/Chem testing[19]		X			
Plasma storage[20]	X	X		X	X
Urine storage[21]		X		X	X
Serum storage for SARS-CoV-2 testing[22]		X		X	X

Footnotes for Appendix IA

Parentheses around an X indicate that this procedure will be done as needed.

[1] Between the Enrollment Visit and the 26-week visit, participants in the intervention arm will engage with study staff for clinical care in the mobile unit at a frequency determined by clinical need. These are considered care visits. Specimen collection and testing at care visits will be as needed for clinical care. Active control arm participants will not have these visits.
[2] Assessment for COVID-19 will consist of a symptom screen and temperature. Details included in the SSP Manual.
[3] Assessment for OUD will be by a validated screening tool to be specified in the SSP Manual. All sites will use the same tool.
[4] History of hospitalization only.
[5] Physical exam at enrollment to include vital signs, height, weight, general appearance, head, ear, nose and throat, neck, chest, abdomen, extremities, skin and a brief neurologic exam. Additional elements at clinician's discretion for patient care.
[6] HIV treatment (or referral) will be offered at the first visit where HIV infection is confirmed, for those participants not already in treatment. Intervention arm participants will be offered HIV treatment in the mobile unit if the available regimen is appropriate for them. Intervention arm participants who require a different regimen and active control arm participants will be referred for treatment. Initiation of MOUD treatment will be the clinical priority, so HIV-positive participants may defer initiating HIV treatment until established on MOUD.
[7] The exact timing of COWS assessment and MOUD initiation will depend on clinician judgment, the readiness of the participant to begin treatment and other factors. See Section 6.2.
[8] Vaccination referral or treatment/treatment referral will be offered at the first visit where results from testing are available.
[9] For participants in the intervention arm receiving care in the mobile health delivery unit, ART regimens can be selected by study clinicians that treat HIV as well as HBV. Active control arm participants will receive referrals indicating their dual infection with HIV and HBV so that they may also receive appropriate treatment.
[10] STI results and referrals provided on a date after results are available, coded as a "split visit".
[11] The types of samples collected (oral, rectal, vaginal) are specified in the SSP Manual.
[12] Other HIV-related testing may be performed for clinical care. This may include HIV drug resistance testing and/or HLA-B5701 testing. If indicated, this testing should be performed at a local laboratory; these results will not be reported to the HPTN SDMC.
[13] Testing for medications used to treat substance use (see SSP Manual).
[14] Testing for substances of abuse (see SSP Manual).
[15] Testing for pregnancy (urine human chorionic gonadotropin [HCG] testing) for any participant who could potentially be pregnant at that visit (unless already known to be pregnant).
[16] Perform HCV Ab testing at enrollment for all participants; perform HCV Ab testing at week 52 for participants who tested HCV negative at enrollment.
[17] Perform HCV viral load testing at enrollment, 26 weeks, and 52 weeks for participants who have a positive HCV Ab test.
[18] Perform HBV viral load testing for participants with chronic HBV infection (HBsAg+) or isolated HBcAb positive for clinical care management (intervention arm only).
[19] The following tests are required: hemoglobin, creatinine, ALT, AST and total bilirubin. Sites may obtain these values by ordering a complete blood count and comprehensive metabolic panel if that is standard practice or less costly than performing individual tests.
[20] Stored plasma will be used for testing at the HPTN LC, as described in Section 10.
[21] Stored urine will be used for testing at the HPTN LC, as described in Section 10.
[22] Stored serum will be used for retrospective testing at the HPTN LC to determine the prevalence of SARS-CoV-2 seropositivity at baseline, 26 and 52 weeks; stored samples may also be used for specialized testing related to COVID-19 (see Section 10 and SSP Manual).

Abbreviations: ART: antiretroviral treatment; aPTT: activated partial thromboplastin time; COWS: Clinical Opiate Withdrawal Scale; GC/CT: gonorrhea/chlamydia; HAV: hepatitis A virus; HBV: hepatitis B virus; HBcAb: HBV core antibody; HBsAb: HBV surface antibody; HBsAg: hepatitis B surface antigen; HCV: hepatitis C virus; HLA:

Human Leukocyte Antigen; IgG; immunoglobulin G; MOUD: medications for opioid use disorder; NAAT: nucleic acid amplification test; OUD: opioid use disorder; PT: prothrombin time; SDMC: Statistical and Data Management Center; SSP: Study specific protocol; STI: sexually transmitted infection.

APPENDIX IB: SCHEDULE OF STUDY VISITS AND PROCEDURES FOR PARTICIPANTS WHO ARE HIV NEGATIVE AT ENROLLMENT

	**Screening**	**Enrollment**	**Care visit(s)[1]**	**26 Weeks**	**52 Weeks**
**Administrative and Behavioral Procedures**					
Informed consent	X				
Locator information	X	X	X	X	X
MOUD counseling	X	X	X	X	X
HIV risk reduction counseling and test results	X	X	(X)	X	X
Offer condoms and lubricant	X	X	X	X	X
Provide/facilitate access to harm reduction	X	X	X	X	X
Demographic information	X				
Randomization		X			
Behavioral data collection		X		X	X
Introduction to peer navigator		X			
Conclusion of peer navigation				X	
**Clinical Evaluations/Procedures**					
Assessment for COVID-19[2]	X	X	X	X	X
Assessment for OUD[3], recent injection drug use (track marks)		X	(X)	X	X
Targeted medical history including MOUD treatment, HIV risk behaviors, participation in other research studies	X	X	(X)	X[4]	X[4]
Basic physical (wellness) exam[5]		X	(X)		
Screen for mental health needs and refer for services as indicated		X	(X)	X	X
PrEP initiation (intervention arm) or referral (active control arm)		(X)	(X)		
COWS assessment and initiate mobile unit-based MOUD treatment program (intervention arm only)		(X[6])	(X[6])		
Provide clinical management of MOUD and PrEP, including medication or prescription dispensation, as indicated			X		
HAV vaccination referral			X[7]		
HBV vaccination referral			X[7]		
HBV treatment/treatment referral			X[7,8]		
HCV treatment referral			X[7]		X
Development of a clinical plan		X			
Empiric treatment of STIs (if symptomatic)		(X)	(X)	(X)	(X)

Lab-based STI results provided and, if indicated, treatment (intervention arm) or referral (active control arm)			X[9]		
Provide lab-based STI results, and, if indicated, referral				X[9]	X[9]
Provide clinical assessment and management or referral for other medical conditions			X		
Blood collection	X	X	(X)	X	X
Urine collection	X	X	(X)	X	X
Swabs for STI testing[10]		X	(X)	X	X
**Laboratory Evaluations/Procedures**					
HIV rapid testing	X	X	(X)	X	X
Laboratory-based HIV testing (see SSP Manual)	X	X	(X)	X	X
MOUD testing (urine dip stick)[11]	X	X		X	X
Substance use testing (urine dip stick)[12]	X	X	(X)	X	X
Pregnancy testing (urine)[13]		X	(X)	(X)	(X)
STI testing (syphilis, GC/CT NAAT)		X	(X)	X	X
HCV Ab testing[14]		X			X
HCV RNA (viral load)[15]		X		X	X
HBV testing (HBsAg, HBsAb, HBcAb)		X			
Other HBV-related testing[16]		(X)			
HAV testing (HAV IgG)		X			
Heme/Chem testing[17]		X			
Plasma storage[18]	X	X	(X)	X	X
Urine storage[19]		X		X	X
DBS storage[20]		X		X	X
Serum storage for SARS-CoV-2 testing[21]		X		X	X

Footnotes for Appendix IB

Parentheses around an X indicate that this procedure will be done as needed.

[1] Between the Enrollment Visit and the 26-week visit, participants in the intervention arm will engage with study staff for clinical care in the mobile unit at a frequency determined by clinical need. These are considered care visits. Specimen collection and testing at care visits will be as needed for clinical care. Active control arm participants will not have these visits.
[2] Assessment for COVID-19 will consist of a symptom screen and temperature. Details included in the SSP Manual.
[3] Assessment for OUD will be by a validated screening tool to be specified in the SSP Manual. All sites will use the same tool.
[4] History of hospitalization only.
[5] Physical exam at enrollment to include vital signs, height, weight, general appearance, head, ear, nose and throat, neck, chest, abdomen, extremities, skin and a brief neurologic exam. Additional elements at clinician's discretion for patient care.
[6] The exact timing of COWS assessment and MOUD initiation will depend on clinician judgment, the readiness of the participant to begin treatment and other factors. See Section 6.2.
[7] Vaccination referral or treatment/treatment referral will be offered at the first visit where results from testing are available.
[8] For participants in the intervention arm receiving clinical care in the mobile unit, a patient-centered discussion will take place between the clinician and the participant regarding the potential risks and benefits of starting PrEP with HBV infection; whether to take PrEP will be the participant's choice. Study staff will provide results and a referral to those in the active control arm with HBV infection, instructing them to discuss the potential risks and benefits of HBV treatment/PrEP with their provider.
[9] STI results and referrals provided on a date after results are available, coded as a "split visit".
[10] The types of samples collected (oral, rectal, vaginal) are specified in the SSP Manual.
[11] Testing for medications used to treat substance use (see SSP Manual).
[12] Testing for substances of abuse (see SSP Manual).
[13] Testing for pregnancy (urine human chorionic gonadotropin [HCG] testing) for any participant who could potentially be pregnant at that visit (unless already known to be pregnant).
[14] Perform HCV Ab testing at enrollment for all participants; perform HCV Ab testing at week 52 for participants who tested HCV negative at enrollment.
[15] Perform HCV viral load testing at enrollment, 26 weeks, and 52 weeks for participants who have a positive HCV Ab test.
[16] Perform HBV viral load testing for participants with chronic HBV infection (HBsAg+) or isolated HBcAb positive for clinical care management (intervention arm only).
[17] The following tests are required: hemoglobin, creatinine, ALT, AST and total bilirubin. Sites may obtain these values by ordering a complete blood count and comprehensive metabolic panel if that is standard practice or is less costly than performing individual tests.
[18] Plasma will be stored at screening, enrollment, 26 and 52 week visits and at any visit where HIV testing is performed. Stored plasma will be used for testing at the HPTN LC, as described in Section 10. This includes testing to determine the seroprevalence of SARS-CoV-2 at baseline, 26 and 52 weeks.
[19] Stored urine will be used for testing at the HPTN LC, as described in Section 10.
[20] Stored DBS will be used for testing at the HPTN LC, as described in Section 10.
[21] Stored serum will be used for retrospective testing at the HPTN LC to determine the prevalence of SARS-CoV-2 seropositivity at baseline, 26 and 52 weeks; stored samples may also be used for specialized testing related to COVID-19 (see Section 10 and SSP Manual).

Abbreviations: aPTT: activated partial thromboplastin time; COWS: Clinical Opiate Withdrawal Scale; DBS: dried blood spot; GC/CT: gonorrhea/chlamydia; HAV: hepatitis A virus; HBV: hepatitis B virus; HBcAb: HBV core antibody; HBsAb: HBV surface antibody; HBsAg: hepatitis B surface antigen; HCV: hepatitis C virus; HLA: Human Leukocyte Antigen; IgG; immunoglobulin G; MOUD: medications for opioid use disorder; NAAT: nucleic acid amplification test; OUD: opioid use disorder; PrEP: pre-exposure prophylaxis; PT: prothrombin time; SDMC: Statistical and Data Management Center; SSP: Study specific protocol; STI: sexually transmitted infection.

APPENDIX IC: ADDITIONAL PROCEDURES FOR PARTICIPANTS WHO HAVE A REACTIVE OR POSITIVE HIV TEST AFTER ENROLLMENT

	Confirmatory visit
**Administrative and Behavioral Procedures**	
HIV counseling and test results	X
**Clinical Evaluations/Procedures**	
Initiate HIV treatment	X
Blood collection	X
**Laboratory Evaluations/Procedures**	
HIV testing (see SSP Manual)[1]	X
HIV viral load	X
CD4 cell count	X
Other HIV related testing[2]	X
Plasma storage[3]	X
DBS storage[4]	X

[1] Site should also ensure that local guidelines for HIV confirmatory testing are followed.

[2] Other HIV testing may be performed for clinical care. This may include HIV drug resistance testing and/or HLA-B5701 testing. If indicated, this testing should be performed at a local laboratory; these results will not be reported to the HPTN SDMC.

[3] Stored plasma will be used for testing at the HPTN LC, as described in Section 10.

[4] Stored DBS will be used for testing at the HPTN LC, as described in Section 10.

APPENDIX II: SAMPLE INFORMED CONSENT FORM- MAIN STUDY

HPTN 094
INTEGRA: A Vanguard Study of Health Service Delivery in a Mobile Health Delivery Unit to Link Persons who Inject Drugs to Integrated Care and Prevention for Addiction, HIV, HCV and Primary Care

Final Version 1.0
15 July 2020
DAIDS Document ID: 38715

Sponsored by: Division of AIDS, US National Institute of Allergy and Infectious Diseases, US National Institutes of Health.

PRINCIPAL INVESTIGATOR: *[Insert Name*]

PHONE: *[Insert Number]*

**<u>Key Information</u>:**
**The first two pages of this document include summary information about this study that will help you decide whether or not you should participate. More detailed information is provided after this summary section.**

About this research
You are being asked to join a research study. Scientists do research to answer important questions which might help change or improve the way we do things in the future. This form explains the research study and your part in the study. Please read it carefully and take as much time as you need. Ask your study doctor or the study team to explain any words or information that you do not understand. You may take this description home and discuss it with your family or friends to help you decide.

Taking part in this research study is voluntary
You may choose not to take part in the study or may choose to leave the study at any time. Deciding not to participate, or deciding to leave the study later, will not result in any penalty or loss of benefits to which you are entitled and will not affect your relationship with the study site.

Important Information
This information gives you an overview of the research. More information about these topics may be found in the pages that follow.

1. Why is this research being done?
This research study will help us see whether providing medical services in a mobile unit will help PWID start and stay on anti-HIV medicines and medicines for opioid use disorder better.

2. What will happen to me during the study?
You will either receive health care services at visits conducted from a mobile unit or you will be guided to regular clinics in the area for your health care. All participants at Screening and Enrollment

will receive laboratory testing to evaluate opiate use, HIV, hepatitis and sexually transmitted diseases. Participants will also receive a physical exam, counseling and develop a clinical plan with the health care providers. All participants will be assigned a peer health navigator to work with them during the study.

3. **How long will I participate in the study?**
   If you decide to join the study, participation will last about 1 year with two visits after enrollment.

4. **Will I benefit from the study?**
   It is possible that you may benefit from taking part in this study; however, there is no guarantee that it will help you. You will get information about your health and the results of the tests, as well as treatment for sexually transmitted infections. The counseling you get during this study may help you avoid HIV and other sexually transmitted infections. For more information, please see below.

5. **Will taking part in the study expose me to risks?**
   Taking part in this research may expose you to risks. We may not know or understand all the risks at this time. It is very important that you understand the risks in this research study before you decide whether you will participate. For details and a list of risks you should know about, please see below.

6. **Will I be paid to participate?**
   Payment for your time or travel is available if you decide that you will take part in this study. For more information, please see below.

7. **Will it cost me anything to participate?**
   There is no cost to you for taking part in this study.

Please review the rest of this document for details about these topics and additional things you should know before making a decision about whether you will participate in this research.

INTRODUCTION

You are being asked to take part in a research study for people who inject drugs (PWID) with opioid use disorder (OUD) who may either be at risk for getting human immunodeficiency virus (HIV) or could pass HIV on to others. HIV is the virus which causes acquired immunodeficiency syndrome (AIDS). This research study will help us see whether providing medical services in a mobile unit will help PWID start and stay on anti-HIV medicines and medicines for opioid use disorder better.

Before you decide whether to join the study, we will explain the purpose of the study, the risks and benefits to you, and what is expected of you if you decide to participate.

A research team member will talk with you about the study, tell you what will happen if you decide to participate in this study and give you this consent form to read. If you do not understand what you are reading please ask the study staff to explain anything you do not understand, including any language contained in this form. You may ask to have this form read to you.

After the study is explained to you and all your questions have been answered, you can decide whether or not you want to join the study. You do not have to make a decision now; you can take the consent document home and share it with family, friends, and your health care provider. Taking part in the study is voluntary which means you can choose whether or not to participate. If you join the study, you can

then choose to leave at any time and it will not affect your eligibility to receive regularly-provided health services.

If you decide to join the study, you will be asked to sign this consent form and a copy of this form will be given to you. Keep this form; in it you will find contact information for the research staff and answers to questions about the study. If you choose not to take a copy of this form, we will give you a card with the research staff contact information.

1. You should know key information about this study before you join.

Here is a summary of important information about the study:

- This is a research study. Research is not the same as routine treatment or medical care. The purpose of a research study is to answer scientific questions. These answers can help find better ways to deliver treatment and improve knowledge of human behavior.
- Your participation in this study is voluntary. You may refuse to join, or you may withdraw your consent to be in the study, at any time, for any reason.
- We are doing this study to see whether providing medical services in a mobile unit will help PWID start and stay on opioid use treatment medications and anti-HIV medications.
- About 860 PWID will participate in this study from cities in the United States. Participants will be in the study for about one year.
- We will collect blood, urine, and swabs from you at study visits to test for sexually transmitted infections (STIs). Collecting blood samples may cause pain, bruise your arm or make you feel lightheaded.
- We will test you for HIV, hepatitis A, B, and C and STIs during the study. You will receive treatment or referral for treatment as appropriate. The STIs we will test for are Chlamydia, gonorrhea, and syphilis. There are other STIs, but we will not test for them as part of this study.
- Before a study visit or at the start of a visit, we will assess you for COVID-19. If you are suspected to have COVID-19, you will need to wait until it is safe for you to be seen by study staff before we can conduct the visit.
- You may be provided medication for opioid use disorder (MOUD) and anti-HIV medications for HIV treatment or prevention, or you will be referred to places where you can get these medications.
- There may be some social risks. You may feel embarrassed or uncomfortable with some of the questions you will be asked, some of the procedures that will be done, or some of the test results that you will receive.
- We will make every effort to protect your confidentiality during the study. However, it is possible that others may learn that you are part of this study.
- The counseling you receive during this study may help you to avoid HIV, hepatitis A, B, and C and other STIs.
- If you join this study you will receive many health services at no charge while you are participating in the research. As well, the information gathered during this study may help to develop ways to prevent HIV and other STIs and to increase use of treatment for OUD among PWID and their partners in your community.

More information is given in this form about the study. You should feel that you understand the study before deciding whether you will participate.

ABOUT THE STUDY

The HIV Prevention Trials Network (HPTN) and *[insert site name]* are doing this study to see whether providing medical services in a mobile unit will help PWID start and stay on medications. The medications we mean here are for treatment of OUD as well as for treatment or prevention of HIV. About 860 PWID will participate in this study from cities in the United States. Participants will be in the study for about one year.

2. The study is testing a program to provide care for HIV and opioid use disorder to PWID in a mobile health delivery unit ("mobile unit").

Opioid use is the leading cause of accidental death in the United States. Injecting drugs can put people at high risk for getting HIV or passing HIV on to others. It can be difficult for people who inject opioids to start or stay on medication treatment offered at clinics. These clinics can be hard to get to and sometimes people have to go to multiple clinics to get all the services they need. We want to see whether providing multiple services in one location—the mobile unit—in a convenient place will make it easier to get treatment for OUD and HIV prevention and treatment. Medications offered in the mobile unit will be the same medications used in the regular clinics in this city. The only difference will be that they are provided in the mobile unit. This study may show a new way to improve the health of PWID.

3. Participants will be placed in 1 of 2 groups.

All participants will be assigned to one of two groups by random chance (the equivalent of throwing dice). The difference between the groups is that the first group will receive health care services delivered on a mobile unit. These services include MOUD, HIV testing, HIV treatment for people living with HIV and not in care, pre-exposure prophylaxis (PrEP) for people who do not have HIV, and STI testing and treatment. The second group will have their initial Screening and Enrollment Visits in the mobile unit, but all other visits will occur in regular clinics in the area.

Participants in both groups will be assigned a peer navigator for 26 weeks who will assist participants in accessing and remaining in medical care.

All participants will come in for study assessments at approximately six and 12 months after starting the study and will be in the study for about a year total.

JOINING THE STUDY

4. It is your decision whether to participate in the study.

This consent form gives information about the study that will be discussed with you. We will help you understand the form and answer your questions before you sign this form. Once you understand the study, and if you agree to take part, you will be asked to sign your name or make your mark on this form. You will be offered a copy of this form to keep.

Before you learn about the study, it is important that you know the following:

- Your participation is voluntary. You do not have to take part in any of the tests or procedures in the study.
- You may decide not to take part in the study, or you may decide to leave the study at any time. Your decision will not affect your eligibility for continuing to receive health care at community-based services.
- If you decide not to take part in the study, you can still join another study at a later time if there is one available and you qualify.

- You cannot join this study if you are taking part in another research study, without special permission. You are asked to tell the study staff about any other studies you are taking part in or thinking of taking part in.

5. **You must qualify before you can join the study.**

If you decide to join this study, we will first do some tests and collect some information from you to find out if you qualify. These tests and the information collected are described in #6 below. If you do not qualify, you cannot join the study.

6. **We will ask you questions, examine you, and test your blood.**

To find out if you qualify, we will first conduct a "Screening Visit." Your Screening Visit will happen after you read, discuss, understand, and sign this form. The Screening Visit will take about X hours [*sites to fill in the amount of time]*.

At the Screening Visit, we will:

- Ask you about symptoms of COVID-19 and take your temperature.
- Ask you some questions about yourself, like your age, and your ethnic group.
- Ask where you live and how to contact you
- Provide drug use and HIV risk reduction counseling
- Collect ~*XX* mL (about *x* teaspoons) [*sites to complete*] of blood for HIV testing
- Collect urine to test for drug use and drug treatment
- Ask you questions about your sexual and drug use behaviors
- Offer you condoms and lubricant and counsel you on how to use them safely
- Provide you with clean needles and dispose of any used needles you have [*sites to include if they are allowed to provide in their location*]
- Offer you a naloxone kit that can be used to treat an overdose, and show you how to use it

We may assess you before or at the start of the visit for COVID-19. If you are suspected to have COVID-19, you will need to wait until it is safe for you to be seen by study staff before continuing with screening for the study.

The initial results of the HIV test will be available immediately. If your initial test results indicate that you have HIV infection, we will perform additional testing in a laboratory to confirm these results and these will be available [*site to insert timeframe of testing*]. A small amount of blood will be stored from this visit. No other samples collected at the time of screening will be kept or used for any other tests other than those listed above.

7. **We will confirm if you qualify for the study.**

If the information you provide and the test results from the Screening Visit indicate that you may be eligible for the study, you will be asked to return for an "Enrollment Visit". This visit will last about X hours [*sites to fill in the amount of time]*.

During the Enrollment Visit, we will first determine if you are eligible to enroll in the study. If you are enrolled, you will complete additional activities.

At the Enrollment Visit we will:

- Ask you questions to assess for COVID-19 infection and take your temperature. If you are suspected to have COVID-19, you will need to wait until it is safe for you to be seen by study staff before continuing with the visit.
- Confirm where you live and how to contact you.
- Collect urine to test for drug use and drug treatment, STIs (if you are male) and, if you are enrolled, to check for pregnancy (if you are someone who can become pregnant)
- Collect ~*XX* mL (about *x* teaspoons) [*sites to complete*] of blood for HIV testing and STIs. If you are enrolled, we will then also test your blood for hepatitis (a liver disease) and to assess the health of your blood and liver.
- Provide you with HIV test results and HIV counseling.
- Ask you about your drug use and treatment history and sexual practices. We will also ask you about other research studies you are participating in.

8. If you qualify, you will enter the study

If you are eligible to participate in the study, you will be enrolled during the Enrollment Visit. You will then be placed by random chance into either the group that will receive medical care in the mobile health delivery unit, or the group that will not. After that, the Enrollment Visit will continue and we will:

- Ask you questions about your medical history and how you feel about how your life is going.
- Collect swabs from you to test for STIs. These swabs may be taken from the throat, rectum and vagina.
- Obtain your medical history, including MOUD treatment, HIV risk behaviors, participation in other research studies
- Give you a physical exam, that includes measuring your height, weight, heart rate, temperature and blood pressure; looking into your ears, mouth and throat; listening to your heart and lungs, feeling your abdomen, looking at your skin, arms and legs, checking your reflexes and strength and asking you about any medicines you are taking.
- We will store blood and urine samples for other testing related to this study. This may include tests for drugs, medications used to treat HIV, and medications to treat substance use. Tests may also be performed to characterize HIV and the body's response to HIV. Similar testing may be performed for hepatitis viruses. The stored blood samples may also be used to learn more about how HIV is spread throughout the community. Blood samples will also be used to test for possible exposure to the virus that causes COVID-19 and to learn more about how the body responds to COVID-19. Results from testing using stored samples will not be returned to you or the study site.
- Introduce you to a peer navigator, someone who has been trained to help you get medical services and be successful in getting treatment for OUD.
- Give you the results of tests that are available during the visit and discuss your health needs.
- If you have symptoms of an STI at the time of the visit, we will provide you with treatment right away. If laboratory testing of the samples collected at this visit show you have an STI, we will let you know, however these results will not be available until after this visit.
- Make a plan for treating your opioid use disorder and for starting anti-HIV medications to treat or prevent HIV.
- Offer you condoms and lubricant and counsel you on how to use them safely.
- Provide you with clean needles and dispose of any used needles you have. [sites to include if they are allowed to provide in their location]
- Offer you a naloxone kit that can be used to treat an overdose and show you how to use it

BEING IN THE STUDY

9. You will have two study visits over a year. In addition to this you will work with your peer navigator several times over the first six months of the study. If you are in the group that will receive medical care in the mobile unit, you will also have medical visits in the mobile unit at various times over the first six months of the study.

Study Visits

If you join the study you will have at least two additional study visits after your Enrollment Visit. These visits will be approximately six months (26 weeks) and 12 months (52 weeks) after the Enrollment Visit.

During the **week 26 and 52 study visits**, we will:

- Ask you questions to assess for COVID-19 infection and take your temperature. If you are suspected to have COVID-19, you will need to wait until it is safe for you to be seen by study staff before continuing with the visit.
- Confirm where you live and how to contact you.
- Ask you questions about your sexual practices, medical history, drug use and drug treatment history and how you feel about how your life is going.
- Talk with you about HIV and ways to protect yourself from getting it if you do not have HIV.
- Collect ~*XX* mL (about *x* teaspoons) [*sites to complete*] of blood for HIV testing if you are not already known to be living with HIV and to test your blood for STIs and hepatitis (a liver disease). We will test the level of HIV virus in your blood (viral load) if you have HIV infection.
- Collect urine to test for drug use and drug treatment, and to check for pregnancy (if you are someone who can become pregnant)
- Collect swabs from you to test for STIs. These swabs may be taken from the throat, rectum and vagina.
- We will store blood and urine samples for other testing related to this study. This may include testing for drugs, medications used to treat HIV and substance use, and other tests related to HIV and HCV infection. The stored blood samples may also be used to learn more about how HIV and HCV are spread throughout the community. Blood samples will also be used to test for possible exposure to the virus that causes COVID-19 and to learn more about how the body responds to COVID-19. Results from testing using stored samples will not be returned to you or the study site.
- Provide treatment if you have symptoms of an STI.
- Give you the results of tests when they are available.
- If your laboratory testing shows that you have an STI, we will refer you for treatment.
- Offer you condoms and lubricant and counsel you on how to use them safely.
- Provide you with clean needles and dispose of any used needles you have. [*sites to include if they are allowed to provide in their location*]
- Offer you a naloxone kit that can be used to treat an overdose and show you how to use it

Each of these visits will last about X hours [*site staff to insert amount of time]*. At your final study visit (week 52 visit), we will talk with you about what will happen when the study is over, including when the results of the study will be available.

Visits with Your Peer navigator

You will work with a peer navigator during the first six months of the study. Your peer navigator will help you address issues that may have made it hard for you to get care in the past. They will act as a coach to help you stay motivated in treatment for opioid use disorder and HIV care or prevention. They will

remind you of medical appointments you have and help you find medical and other resources available to you in your community.

You will mostly work with your navigator in-person at the start of the six months. Over time you may work together more over the phone/through messaging apps. The study will provide airtime minutes to allow participants who have phones to continue to receive peer navigation. If you are in the group that receives medical care in the mobile unit, toward the end of the six months, your navigator will help you transition your medical care to a clinic or clinics in your community. If you are in the group that does not get medical care in the mobile unit, your navigator will work with you to get medical care at a clinic or clinics in your community right from the start.

How often you interact with your navigator and how long these interactions last will depend on your needs. We expect you will be in contact with your navigator multiple times per month.

Medical Care Visits in the Mobile Unit
If you are put in the group that will receive medical care in the mobile unit, you will have multiple visits to the mobile unit over the first six months of the study. How often you have visits and how long they last will depend on your medical needs. This may change over time.

Note that if you already receive medical care from providers in the community and are happy with this care, you do not have to switch to care in the mobile unit. You can also choose to get some care in the mobile unit and other care from regular clinics in the community.

A primary goal of the care you receive in the mobile unit will be to provide you with medical treatment for opioid use disorder. Another primary goal is to provide you with HIV treatment (ART) if you are living with HIV or to provide you with HIV prevention (PrEP) if you are not living with HIV.

What happens at a medical care visit will depend on your medical needs at the time. We would expect that they would include at least some of the following:

- Ask you questions to assess for COVID-19 infection and take your temperature. If you are suspected to have COVID-19, you will need to wait until it is safe for you to be seen by study staff before continuing with the visit.
- Confirm where you live and how to contact you.
- Ask you questions about your sexual practices, medical history, drug use and drug treatment history and how you feel about how your life is going.
- Talk with you about HIV and ways to protect yourself from getting it if you do not have HIV
- Give you a physical exam that may include measuring your weight, temperature, blood pressure, looking into your mouth and throat, listening to your heart and lungs, feeling your abdomen (stomach and liver), and asking you about any medicines you are taking.
- Begin HIV treatment if you are living with HIV. Offer PrEP if you are HIV negative.
- Provide medications to treat opioid use disorder.
- Provide referral for vaccinations for hepatitis A and B.
- Provide referral for treatment, if you are infected with hepatitis B or C.
- Collect ~*XX* mL (about *x* teaspoons) [*sites to complete*] of blood for HIV testing if you are not already known to be living with HIV and to test your blood for STIs. We will test the level of HIV virus in your blood (viral load) and the health of your blood (CD4 cell count) if you have HIV infection.
- Collect urine to test for drug use, and to check for pregnancy (if you are someone who can become pregnant).

- Collect swabs from you to test for STIs. These swabs may be taken from the throat, rectum and vagina.
- We will store blood samples for other testing related to this study at any visit where HIV testing is done. This may include testing for medications used to treat HIV and other tests related to HIV and HCV infection. The stored blood samples may also be used to learn more about how HIV and HCV are spread throughout the community. Results from testing using stored samples will not be returned to you or the study site.
- Provide treatment if you have symptoms or laboratory results indicating an STI.
- Give you the results of tests when they are available.
- Offer you condoms and counsel you on how to use them safely.
- [*Sites to include if allowed at their location*: "Provide you with clean needles and dispose of any used needles you have."]
- Offer you a naloxone kit that can be used to treat an overdose and show you how to use it

10. If you stop participating in peer navigation, or stop receiving OUD or HIV care or prevention, we will ask you to stay in the study.

We hope that if you join this study, you will work with your peer navigator for the full six months. We also hope that you will start and stay on treatment for OUD and receive HIV treatment or prevention for the full six months (in the mobile unit if that is your group). But if you stop these activities for any reason, we still ask you to continue to come for the study visits at 26 and 52 weeks. The data you provide at these study visits will still be important to the outcome of this study. You always have the right to stop participating in the study completely at any time.

11. If you acquire HIV during the study, we will help you get care and support.

We will test your blood for HIV during this study. If you are HIV-negative and get HIV while you are in the study, you will stop taking PrEP if you started PrEP, and we will provide or help you find the care and support you need. You will still continue to receive peer navigation through six months and will continue to receive medical care in the mobile unit, if you are in the group that receives care in the mobile unit.

12. We will test you for pregnancy during the study if you are someone who can become pregnant.

We will collect urine to test for pregnancy and will give you these results each time. If you become pregnant during the study, you will be able to stay in the study as originally planned. If you are pregnant at the last study visit, we will contact you after that to find out the outcome of the pregnancy.

Even if you are in the group to receive medical care in the mobile unit, the study cannot provide all the medical care you will need for a pregnancy or delivery of a baby. The baby will not be in the study. Therefore, it is important to receive medical care for a pregnancy outside the study. We will tell you where you can go for the care you need.

If you are taking ART or PrEP and become pregnant, we will provide some basic information about your pregnancy, such as what medications you took while pregnant, what prenatal care you received, and the outcome of your pregnancy, to the Antiretroviral Pregnancy Registry. None of your personal information, such as your name or address, will be provided to the registry. You can learn more about this registry at www.apregistry.com.

13. Some of your blood and urine may be left over at the end of the study and may be used for future research, if you provide consent.

Some of your blood and urine collected for this study may be left over after all of the study tests are completed. If you agree, these stored samples may also be used for future research related to HIV infection, hepatitis infection, COVID-19, and other infections, and to better understand laboratory tests related to this study. This research will not include whole genome sequencing also known as WGS.

You will be asked to sign this consent form to give permission to use your stored samples for future research. Even if you do not give permission to store your blood for possible future research, you can still be in this study. If you give permission, you will not be asked to give permission again during the study. However, you may withdraw your consent to use your stored samples for future research at any time. We will then destroy your samples after all of the study-related testing has been completed. If you agree to have your stored samples used for future research, there is no time limit on how long your samples will be stored. The stored samples will be labeled only with your study number and will be tested at the HPTN Laboratory Center (LC) or laboratories designated by the HPTN LC. We will not share the key that says which study number is yours so the laboratory doing the testing will not know who you are. Only approved researchers will have access to your samples. Results from this testing will not be returned to the study site or you. Your samples will not be sold or directly used to produce commercial products or for commercial gain. All proposed research studies using your samples will be reviewed by the National Institutes of Health (NIH).

RISKS OF THE STUDY

14. There may be risks to being in this study.

STUDY PROCEDURES

Taking blood samples may cause some pain, bruise your arm, or make you feel lightheaded. In rare cases you may faint. There is also a slight chance of infection when blood is drawn. You may be nervous while you are waiting for your HIV or other test results. If the tests show that you have HIV or another infection, you may worry about your health and future. You will receive counseling before and after the test to help address your concerns.

You may experience pain or discomfort in your throat, rectum or vagina from the swab. In some cases, you may have some bleeding.

You will be tested for gonorrhea, chlamydia, syphilis, and hepatitis. [*Note to sites: Insert here any reporting responsibilities for your state or local jurisdictions or reporting of these infections to public health authorities. Also include whether if a participant tests positive, the results will become part of public health records, or any other record (medical file, etc.)]*.

DISCLOSURE OF PERSONAL INFORMATION

We will make every effort to protect your confidentiality during the study. However, it is possible that others may learn that you are part of this study and they may think that you are living with HIV or are at high risk for acquiring HIV. They could make assumptions about your use of drugs. Because of this, you could face stigma related to HIV or drug use. This could cause you trouble finding or keeping a job. You could also have problems with your family, friends and community. We can tell you more about how we will protect your information.

SENSITIVE QUESTIONS

The questions we will ask you about your sexual behavior or drug use may make you feel uneasy. However, you do not have to answer any question that you do not want to and you can stop answering the questions at any time.

SIDE EFFECTS OF MEDICATIONS

If you are assigned to the group that **will NOT receive medical care in the mobile health delivery unit**, you may receive medications from the study staff at the Enrollment Visit. These will be medications to treat STIs, if you have STI symptoms at the Enrollment Visit. The clinician will explain to you any side effects associated with those medications. After the Enrollment Visit, your peer navigator will help you receive care at the regularly available clinics in your community. The staff at those facilities can explain to you the side effects of any medications they give to you or prescribe for you. You will also be offered naloxone kits during your study participation that can be used to treat an overdose. The side effects of naloxone will be explained to you.

If you are assigned to the group that **WILL receive medical care in the mobile unit**, you will receive or be prescribed medications by the study staff during the first six months of your participation in the study. The drugs you could be given on the **mobile unit** include medications to treat or prevent STIs or other bacterial infections, hepatitis, HIV, opioid use disorder or overdose. You may also be prescribed medications that you will need to pick up at a pharmacy. The clinician will explain to you the side effects of the specific medications you are prescribed or given.

IF YOU ARE PREGNANT OR BECOME PREGNANT WHILE PARTICIPATING IN THE STUDY

The medications provided in this study are the same types of drugs that would be provided as part of regular care at clinics in your community and are commonly provided to pregnant people. There is no greater risk to you by receiving them as part of this study. If you are in the group that receives medical care in the **mobile unit**, your clinician will tell you if the medications you are taking have any risks or side effects that apply to pregnant people. If you are in the group that does not receive medical care in the **mobile unit**, you should tell your clinician that you are pregnant so that they can explain to you any risks or side effects of your treatment that apply to pregnant people.

BENEFITS OF THE STUDY

15. There may be direct and indirect benefits to you from participating in the study.

If you participate in this study, you will receive many health services at no charge. You will be tested for HIV, hepatitis A, B, and C, and other STIs. You will receive treatment or referral for treatment, as appropriate, if you have these infections. The counseling you get during this study may help you to avoid HIV, hepatitis A, B, and C, and other STIs. If you have or get HIV, this counseling may help you to learn how to better care for yourself and avoid passing HIV to your sexual partners (or fetus, if you are or become pregnant). If you do not have HIV, you will be offered PrEP or referral for PrEP to help you avoid getting HIV. Your peer navigator will work to link you to health services, including treatment for OUD and HIV care or prevention services. You will receive free condoms and lubricant, and naloxone kits for overdose reversal. If you are put in the group that receives medical services in the mobile unit, you will be provided with medical care and medication in a single convenient location.

If you are pregnant or become pregnant while participating in the study, there may be benefits to your fetus from participating in this study. Being on medical treatment for OUD may let you take better care of yourself, which will benefit your fetus. Taking OUD medication may decrease your use of street

drugs, which can also benefit your fetus. Taking medication to either treat or prevent HIV infection while you are pregnant can help you prevent passing HIV on to your fetus.

You may receive indirect benefits from this study. The information gathered during this study may show how medical services can be provided more successfully to people who inject drugs, particularly services for OUD and HIV. This could influence how medical services are provided in the future and may be beneficial to you and your community.

OTHER INFORMATION ABOUT THE STUDY

16. Some of the information we collect from you for this study may be used for other research.

Information collected from this study may be used for other studies carried out by this team or by other researchers. The information we share with other researchers would never include your name or any other information that could identify you.

17. We will tell you any new information that may affect your decision to be in the study.

You will be told any new information learned during this study that might affect your willingness to stay in the study. You will also be told when the results of the study may be available, and how to learn about them.

18. You may be withdrawn from the study without your consent.

We may take you out of the study at any time without your consent. This may happen if:

- You are unable or unwilling to follow all of the study procedures or instructions.
- The study is stopped or canceled.
- The study staff feels that staying in the study would be harmful to you.
- Other reasons, as decided by the study staff.

If we take you out of the study, we may ask you to come back to the clinic one last time to collect a blood sample and ask you questions.

19. You have other choices if you choose not to be in this study.

[*Sites to include/amend the following if applicable]* There may be other studies going on here or in the community that you may be eligible for. If you wish, we will tell you about other studies that we know about. There also may be other places where you can go for HIV counseling and testing, HIV treatment or PrEP, and medication for OUD. We will tell you about those places if you wish.

20. There is no cost to you to be in this study.

There will be no cost to you for study related visits, physical examinations, laboratory tests, or other procedures or medications you receive in the mobile health unit.

The study will not pay for any medications or health services you receive outside of the mobile unit. This is true even if you are given a prescription or a referral for these by the staff in the mobile unit. However, study staff will help connect you to health services in the community that may be free or low-cost and will help you to sign up for health insurance that you are eligible for.

21. **We will give you [site to insert amount] for each study visit.**

You will receive [$xx] for your time, effort, and travel to and from the clinic at each scheduled visit. We may invite you to refer people you know to enroll in this study and offer to pay you if those people enroll. If so, you will receive [$xx] for each of these referrals. [*Sites to insert information about local reimbursement for the study.*]

22. **We will do our best to protect your private information.**

Every effort will be made to keep your personal information confidential, but we cannot guarantee absolute confidentiality. To keep your information private, the samples of your blood and urine that are used for research purposes will be labeled with a code that can only be traced back to your study clinic. Your name, where you live, and other personal information will be protected by the study clinic. The results of any tests done on these research samples will not be included in your health records. You will be identified by a code, and personal information from your records will not be released without your written permission. Any publication of this study will not use your name or identify you personally. Your personal information may be disclosed if required by law.

Clinic staff will have access to your study records. Your records may also be reviewed, under guidelines of the US Federal Privacy Act, by:

- The [*insert name(s) of Single Institutional Review Board (IRB)]*
- The sponsor of the study (US National Institutes of Health [NIH]), its contractors, and its study monitors
- the National Institute on Drug Abuse (NIDA) and/or their contractors
- The US Office for Human Research Protections (OHRP)
- other local, US, and international regulatory entities
- Personnel of the HPTN research network that is conducting this study
- And (*insert any other applicable local authorities*]

In addition to the efforts made by the study staff to help keep your personal information confidential, we have obtained a Certificate of Confidentiality from the US Federal Government. This Certificate protects researchers from being forced to tell people who are not connected with this study, such as the court system, about your participation. The Certificate of Confidentiality does not prevent you from releasing information about yourself and your participation in the study. The Certificate cannot be used to resist a demand for information from personnel of the US Government that is used for auditing or evaluation of federally funded projects or for information that must be disclosed in order to meet the requirements of the US FDA. Even with the Certificate of Confidentiality, if the study staff learns of possible child abuse and/or neglect or a risk of harm to you or others, we will tell the proper authorities.

The study staff will also use your personal information, if needed, to verify that you are not taking part in any other research studies. This includes other studies conducted by [*site name*] and studies conducted by other researchers that study staff know about.

[Sites to include/amend the following if applicable:] [*Local/state/national*] regulations require study staff to report the names of people who test positive for [*HIV and other infections*] passed during sex to the [*local health authority*]. Outreach workers from the [*health authority*] may then contact you about informing your partners, since they also should be tested. If you do not want to inform your partners

yourself, the outreach workers will contact them, according to the confidentiality guidelines of the [*health authority*].

The virus in one person with HIV is slightly different from the viruses in other people. If you give us permission to save your samples for future testing, and you are HIV positive, we may use special laboratory tests to compare the virus in your blood to the virus from other people with HIV. This can help us understand how HIV is spread throughout communities. The samples used for this testing will not include any information that would identify you, like your name. This is to prevent anyone from being able to connect any information we learn about your HIV to you. The results of this analysis will only be used for research.

23. If you get sick or injured during the study, contact us immediately.

[Sites to specify institutional policy:] It is unlikely that you will be injured as a result of study participation. If you are injured, the [*institution*] will give you immediate necessary treatment for your injuries. You [*will/will not*] have to pay for this treatment. You will be told where you can get additional treatment for your injuries. There is no program to pay money or give other forms of compensation for such injuries either through this institution or the US NIH. You do not give up any legal rights by signing this consent form.

24. Contact us at any time if you have questions or problems.

If you ever have any questions about the study, or if you have a research-related injury, you should contact [*insert name of the investigator or other study staff*] at [*insert telephone number and/or physical address*].

An institutional review board (IRB) is an independent committee established to help protect the rights of research participants. If you have any questions about your rights as a research participant, and/or concerns or complaints regarding this research study, contact**:**

- By mail:
  Study Subject Adviser
  Advarra IRB
  6940 Columbia Gateway Drive, Suite 110
  Columbia, MD 21046
- or call **toll free**: 877-992-4724
- or by **email**: adviser@advarra.com

If you have questions about who to contact at the research site, you should contact [insert name of the investigator or community educator or CAB member] at [insert physical address and telephone number]

SIGNATURE PAGE

HPTN 094
INTEGRA: A Vanguard Study of Health Service Delivery in a Mobile Health Delivery Unit to Link Persons who Inject Drugs to Integrated Care and Prevention for Addiction, HIV, HCV and Primary Care

Final Version 1.0
15 July 2020

(Modify as needed per protocol requirements)
Insert signature blocks as required by the local IRB:] If you have read this consent form, or had it read and explained to you, and you understand the information, and you voluntarily agree to join the study, please sign your name or make your mark below. Also, please indicate by providing your initials in the spaces below if you agree to long-term storage and future testing of your samples.

________ I agree to take part in this study.

________ I agree to have samples of my blood stored for future testing related to HIV infection, HCV infection, COVID-19, other infections, and other goals of this study.

________ I agree to have samples of my urine stored for future testing related to HIV infection, HCV infection, COVID-19, other infections, and other goals of this study

________ I do not agree to have samples of my blood stored and used for future testing.

________ I do not agree to have samples of my urine stored and used for future testing.

____________________	____________________
Participant Name (print)	Participant Signature and Date
____________________	____________________
Study Staff Conducting<br>Consent Discussion (print)	Study Staff Signature and Date
____________________	____________________
Witness Name (print)<br>(As appropriate)	Witness Signature and Date

APPENDIX III: SAMPLE INFORMED CONSENT FORM- QUALITATIVE INTERVIEWS

HPTN 094
INTEGRA: A Vanguard Study of Health Service Delivery in a Mobile Health Delivery Unit to Link Persons who Inject Drugs to Integrated Care and Prevention for Addiction, HIV, HCV and Primary Care

Final Version 1.0
15 July 2020
DAIDS Document ID: 38715

Sponsored by: Division of AIDS, US National Institute of Allergy and Infectious Diseases, US National Institutes of Health.

PRINCIPAL INVESTIGATOR: *[Insert Name*]

PHONE: *[Insert Number]*

KEY INFORMATION

- Participation in this research study is entirely voluntary.
- This study is part of a larger research project focused on people who inject drugs with opioid use disorder. That project is testing whether providing medical services in a mobile unit can help people who inject drugs stay on anti-HIV medicines and on medicines for opioid use disorder.
- This research study will involve conducting interviews with different kinds of people to better understand what makes it easier or harder to successfully offer medical services in a mobile unit to people who inject drugs. Your participation will consist of a one-time, audio-recorded interview, conducted in-person or via phone/internet, and is expected to last 30-60 minutes.
- Risks or discomforts associated with participating in this research study include potential feelings of discomfort due to the interview questions, as well as potential breaches of confidentiality and privacy.
- It is unlikely that you will receive any direct benefit from participating in this research study. We hope that the information gathered from these interviews lead to better health options in the future for people who inject drugs.

INTRODUCTION

You are being asked to participate in an interview to help us understand what makes it easier or harder to successfully offer medical services in a mobile unit to people who inject drugs. We will interview different kinds of people to answer this question, potentially including leaders in the community, health care providers, people who inject drugs and people who work with people

who inject drugs, law enforcement, harm reduction leaders and advocates, medical examiners/coroner's office, and scientists who are involved in studying PWID in the area.

VOLUNTARY PARTICIPATION

Your participation in this study is voluntary. You may refuse to join, or you may withdraw your consent to be in the study, at any time, for any reason. *[For potential interviewees who are participants in the main study: "*If you decide not to take part in this study, or if you join this study and then decide to leave, it will not alter the medical care you are eligible to receive. It also will not affect your ability to participate in the main study."*]* [*For potential interviewees who are staff members at a site*: "If you decide not to take part in this study, or if you join this study and then decide to leave, it will not have any effect on your employment. You have the same right to decide whether to participate as main study participants do and will not be viewed as "uncooperative" if you choose not to participate."] Although we hope that you will be comfortable answering all of the questions openly and honestly, please remember that you may refuse to answer any of the questions, or stop participating in the interview completely, at any time.

Before you decide whether to join the study, we would like to explain the purpose of the study, the risks and benefits to you, and what is expected of you.

This consent form gives information about the study that will be discussed with you. We will help you understand the form and answer your questions before you sign this form. Once you understand the study, and if you agree to take part, you will be asked to sign your name or make your mark on this form. You will be offered a copy of this form to keep.

PURPOSE OF THE STUDY

The purpose of this study is to learn what makes it easier or harder to successfully offer medical services in a mobile unit to people who inject drugs. The information you give in this interview will be combined with information collected during the main research study. We expect to interview approximately 195 people (39 people total in this city) from the cities where the main research study is being conducted. We hope that in the future the information collected in this study will allow medical care to better meet the needs of people who inject drugs.

STUDY PROCEDURES

If you decide to participate in this study, you will have one interview. The interview will be conducted by a member of the research team either in person or via phone. They will ask you where you live and how to contact you. They will ask you specific interview questions to address the overall question

[*for local public health officials: "what public health infrastructures would be needed to deliver medical services in a mobile unit to persons who inject drugs in the absence of this study?"]*
[*for staff* at existing community-based services*: "in what ways does delivery of medical services in a mobile unit affect how people who inject drugs are served in this community?"*]
[*for staff* on the moble mobile unit*: "what are the resource and personnel needs you have had to address to deliver medical services to people who inject drugs on a mobile unit?"*]
[*for study participants: "in what ways can having medical services in a* mobile unit *affect the health of people who inject drugs in this city?"*]

To help make sure that we fully understand your answers, the interviews will be audio-recorded. The information on the audio-recording will then be turned into a transcript (a written record of the conversation) by an individual who works with the research team. Your name will not be included in that written record.

- The interview requires only one study visit and **will take 30-60 minutes** to complete.
- There will be **no cost to you** to participate in the interview.
- **You will receive [*site to fill-in*]** for your time and effort.

RISKS AND/OR DISCOMFORTS

The risk to you in participating in this interview is that some of the questions may be uncomfortable or make you feel uneasy. However, you do not have to answer any question that you do not want to and you can stop answering the questions at any time. You will also be provided with contact and referral information if any of the questions raise issues that you would like to address at this or some later time. Other possible risks associated with this study may include breaches of confidentiality. Although study sites will make every effort to protect your privacy and confidentiality, it is possible that your involvement in the study could become known to others, and that social harms (e.g., unfair or discriminatory treatment) may result. To reduce the likelihood of these risks, all interviews will be conducted in a private setting, and names/identifying information will be removed from transcriptions to protect confidentiality.

BENEFITS

It is unlikely that you will receive any direct benefit from being in this study; however, information gathered during this study may lead to better health options in the future for people who inject drugs.

STUDY RESULTS

You will be told when the results of the study may be available, and how to learn about them.

WHY YOU MAY BE WITHDRAWN FROM THE STUDY WITHOUT YOUR CONSENT

You may be withdrawn from the study without your consent if any of the following occur:

You are unable or unwilling to follow all of the study procedures or instructions.

The study is stopped or canceled.

The study staff feels that staying in the study would be harmful to you.

You are not able to complete all study procedures.

Other reasons, as decided by the study staff.

CONFIDENTIALITY

The research team will protect your confidentiality by not putting your name on any audio files or interview transcripts. These items will be labeled with a code that can only be traced back to your study clinic and these items will be kept in a secure location that can only be accessed by the study staff. Your name and other personal information will be protected by the study clinic. Your information collected as part of this research study will not be used or distributed for future studies even if identifiers are removed.

Efforts will be made to keep your study records and test results confidential to the extent permitted by law. However, we cannot guarantee absolute confidentiality. Your personal information may be disclosed if required by law. You will be identified by a code, and personal information from your records will not be released without your written permission. Any publication of this study will not use your name or identify you personally. However, your records may be reviewed, under guidelines of the US Federal Privacy Act, by the sponsor of the study (US National Institutes of Health [NIH]) and/or its authorized representatives, the Institutional Review Board (IRB), study staff, study monitors, [*insert applicable local authorities*] and other local, US or international regulatory authorities.

In addition to the efforts made by the study staff to help keep your personal information confidential, we have obtained a Certificate of Confidentiality from the US Federal Government. This Certificate protects researchers from being forced to tell people who are not connected with this study, such as the court system, about your participation. The Certificate of Confidentiality does not prevent you from releasing information about yourself and your participation in the study. The Certificate cannot be used to resist a demand for information from personnel of the US Government that is used for auditing or evaluation of federally funded projects or for information that must be disclosed in order to meet the requirements of the US FDA. Even with the Certificate of Confidentiality, if the study staff learns of possible child abuse and/or neglect or a risk of harm to you or others, we will tell the proper authorities.

Your records may be reviewed by:

the US NIH

the US Department of Heath and Human Services (DHHS), Office for Human Research Protection (OHRP)

the National Institute on Drug Abuse (NIDA) and/or their contractors

[insert name of IRB]

US, local or international regulatory authorities/entities

study staff

study monitors

Other regulatory agencies

RESEARCH-RELATED INJURY

[Sites to specify institutional policy:] It is unlikely that you will be injured as a result of study participation. If you are injured, the [*institution*] will give you immediate necessary treatment for your injuries. You [*will/will not*] have to pay for this treatment. You will be told where you can get additional treatment for your injuries. There is no program to pay money or give other forms of compensation for such injuries either through this institution or the US NIH. You do not give up any legal rights by signing this consent form.

PROBLEMS OR QUESTIONS

If you have any questions about the study, or if you have a research-related injury, you should contact [*insert name of the investigator or other study staff*] at [*insert telephone number and/or physical address*].

If you have questions about your rights as a research participant, you should contact *[insert name or title of person on the IRB or other organization appropriate for the site]* at *[insert physical address and telephone number].*

If you have questions about who to contact at the research site, you should contact *[insert name of the investigator or community educator or CAB member]* at *[insert physical address and telephone number].*

SIGNATURE PAGE

HPTN 094
INTEGRA: A Vanguard Study of Health Service Delivery in a Mobile Health Delivery Unit to Link Persons who Inject Drugs to Integrated Care and Prevention for Addiction, HIV, HCV and Primary Care

Final Version 1.0
15 July 2020

If you have read this consent form, or had it read and explained to you, and you understand the information, and you voluntarily agree to join the study, please sign your name or make your mark below.

____________________________	______________________________________
Participant Name (print)	Participant Signature and Date
____________________________	______________________________________
Study Staff Conducting Consent Discussion (print)	Study Staff Signature and Date
____________________________	______________________________________
Witness Name (print) *(As appropriate for illiterate participants)*	Witness Signature and Date

STATISTICAL ANALYSIS PLAN

INTEGRA: A Vanguard Study of Health Service Delivery in a Mobile Health Delivery Unit to Link Persons who Inject Drugs to Integrated Care and Prevention for Addiction, HIV, HCV, and Primary Care

HPTN 094 (Protocol Version #2.0)

Date SAP finalized for signature: *10 FEB 2025*

Effective Date: Effective on the Date of Final Signature

SAP Version: 2.0

STATISTICAL ANALYSIS PLAN

**Protocol Name:**	*INTEGRA: A Vanguard Study of Health Service Delivery in a Mobile Health Delivery Unit to Link Persons who Inject Drugs to Integrated Care and Prevention for Addiction, HIV, HCV and Primary Care*
**Protocol Number:**	*HPTN094*
**Author(s):**	*Rahul PaulChoudhury, SRA IV; Timothy Skalland, Staff Scientist; Brett S Hanscom PhD, Sr Staff Scientist*
**Version:**	*2.0*

Author(s):

**Lead Statistical Research Associate**	
**Legal Name**	Rahul PaulChoudhury
**Job Title**	Statistical Research Associate IV
**Signature & Date**	See eTMF signature manifest

**Protocol Statistician**	
**Legal Name**	Timothy Skalland
**Job Title**	Staff Scientist
**Signature & Date**	See eTMF signature manifest

**Protocol Statistician**	
**Legal Name**	Brett Hanscom
**Job Title**	Senior Staff Scientist
**Signature & Date**	See eTMF signature manifest

The effective date of this document is the date of the latest signature.

All proprietary rights reserved

Approver(s):

Protocol Chair	
Legal Name	Steven Shoptaw
Job Title	Vice Chair of Research and Professor in Family Medicine
Signature & Date	Steven Shoptaw Ph.D.<br>12-FEB-2025

The effective date of this document is the date of the latest signature.

All proprietary rights reserved

TABLE OF CONTENTS

1. LIST OF ABBREVIATIONS AND ACRONYMS 5

2. INTRODUCTION 6

2.1 GENERAL DESIGN CONSIDERATIONS 6
2.2 STUDY OBJECTIVES AND ENDPOINTS 7
2.3 RANDOMIZATION 11
2.4 BLINDING 11
2.5 SAMPLE SIZE AND POWER 11
2.6 ACCRUAL AND RETENTION 11

3. GENERAL DATA ANALYSIS CONSIDERATIONS 12

3.1 ANALYSIS SET(S) 12
3.2 STATISTICAL ANALYSIS ISSUES 12

4. INTERIM ANALYSIS AND DATA MONITORING COMMITTEE 13

5. GENERAL ANALYSIS METHODS 13

6. TRIAL PARTICIPANT DISPOSITION 14

6.1 DISPOSITION OF PARTICIPANTS 14
6.2 PROTOCOL DEVIATIONS 15

7. BASELINE DATA 15

8. EFFICACY/EFFECTIVENESS ANALYSES 15

9. SAFETY ANALYSES 16

9.1 ADVERSE EVENTS AND DEATHS 16
9.2 OTHER SAFETY MEASURES 16

10. SECONDARY ENDPOINTS 16

11. REFERENCES 21

12. CHANGE HISTORY 21

1. LIST OF ABBREVIATIONS AND ACRONYMS

A list of abbreviations used in the SAP

Term/Abbreviation	Definition
AE	Adverse Event
ART	Anti-Retroviral Therapy
COVID-19	Coronavirus disease 2019
DAIDS	Division of AIDS
DBS	Dried Blood Spot
FDA	Food and Drug Administration
GLM	Generalized Linear Model
HAV	Hepatitis A
HBV	Hepatitis B
HCV	Hepatitis C
HIV	Human Immunodeficiency Virus
HPTN	HIV Prevention Trials Network
mL	Milli-Liter
MOUD	Medication for Opioid Use Disorder
OUD	Opioid Use Disorder
PrEP	Pre-Exposure Prophylaxis
PWID	Persons Who Inject Drugs
RNA	Ribonucleic Acid
RSC	Radiation Safety Committee
SAE	Severe Adverse Event
SAP	Statistical Analysis Plan
SARS-CoV-2	Severe Acute Respiratory Syndrome Coronavirus 2
SMC	Safety Monitoring Committee
SSP	Study Specific Procedures
STI	Sexually Transmitted Infection
VL	Viral Load

2. INTRODUCTION

This statistical analysis plan (SAP) details the final analyses and statistical procedures that address the study objectives specified in Version 2.0 of Protocol HPTN 094: INTEGRA: A Vanguard Study of Health Service Delivery in a Mobile Health Delivery Unit to Link Persons who Inject Drugs to Integrated Care and Prevention for Addiction, HIV, HCV and Primary Care. New versions of the SAP will be issued to document updates and changes in the plan.

2.1 General Design Considerations

The following is a protocol summary of the study:

**Protocol title:**	INTEGRA: A Vanguard Study of Health Service Delivery in a Mobile Health Delivery Unit to Link Persons who Inject Drugs to Integrated Care and Prevention for Addiction, HIV, HCV, and Primary Care
**IND Sponsor:**	DAIDS
**Protocol Chair(s):**	Steve Shoptaw, PhD; Nabila El-Bassel, PhD
**Sample Size:**	A total of 450 participants allotted in a 1:1 ratio to intervention and active control arms, with targets of a minimum 25% women and 25% participants under 30 years of age, and 10% people living with HIV at enrollment.
**Study Population:**	People living with and without HIV and who inject drugs, have OUD, and are not receiving MOUD
**Study Sites:**	Five urban sites in the United States (US) with substantial populations of PWID with OUD. The locations are as follows: Houston, TX; Los Angeles, CA; Bronx, NY; Philadelphia, PA; Washington, DC
**Study Design:**	A two-arm, individually randomized, controlled, open label study
**Study Duration:**	Approximately three and a half years with a possibility of extension, with individual participants on study for approximately 52 weeks (26 weeks receiving the intervention or peer navigation and then an evaluation for durability of effect at the end of 52 weeks).
**Study Products:**	Mobile health delivery unit ("mobile unit") to deliver "one stop" integrated health services – particularly medication for opioid use disorder (MOUD) and medication for HIV treatment and prevention – to people who inject drugs (PWID) with opioid use disorder (OUD).<br>VS<br>Health services available at community-based agencies
**Study Regimen:**	All potential study participants will provide biological samples and self-reported data via interview in the mobile unit at Screening and Enrollment Visits. Samples will be tested for HIV, hepatitis A (HAV), B (HBV) and C (HCV) and sexually transmitted infections (STIs). At the Enrollment Visit, participants who meet all inclusion and no exclusion criteria will be randomized to the intervention or active control arm and will receive harm reduction services and empiric treatment for STIs if symptomatic. Participants will also be assessed for COVID-19 at study visits; participants with suspected COVID-19 or recent

All proprietary rights reserved

exposure will be referred for further evaluation, care and/or treatment, as available.

Intervention Arm: Participants in the intervention arm will be provided integrated health services delivered in the mobile unit and peer navigation for 26 weeks. The integrated health services in the mobile unit will include:
MOUD and harm reduction services for OUD
HIV testing
HIV treatment for people living with HIV not already in care
PrEP for people without HIV
Testing and referral for vaccination or treatment for HAV and HBV
Testing and referral for treatment for HCV
STI testing and treatment
Primary care
Harm reduction services

Peer navigation in the intervention arm will coordinate and facilitate integrated care in the mobile unit through 26 weeks. As participants become established in care, navigation will help transition participants to community-based services by 26 weeks after randomization.

Active Control Arm: Participants in the active control arm will be provided 26 weeks of peer navigation to connect them to health services available at community-based agencies.

All participants (both arms) will have study visits at 26 and 52 weeks for evaluation of study endpoints.

2.2 Study Objectives and Endpoints

Objectives:

Primary:

To evaluate whether 26 weeks of “one stop” integrated health services delivered in a mobile unit, supported by peer navigation, improves use of MOUD and increases use of PrEP, as measured at 26 weeks among people without HIV, when compared to 26 weeks of peer navigation to similar health services available at community-based agencies.

Secondary:

1) To evaluate whether 26 weeks of “one stop” integrated health services delivered in a mobile unit, supported by peer navigation, compared to 26 weeks of peer navigation to similar health services available at community-based agencies:
   a) improves use of MOUD at 52 weeks
   b) increases rates of viral suppression among people living with HIV at 26 and 52 weeks
   c) increases use of PrEP among people without HIV at 26 and 52 weeks
   d) decreases opioid and polysubstance use at 26 and 52 weeks
   e) decreases prevalence of bacterial STIs at 26 and 52 weeks
   f) decreases fatal and non-fatal overdose events by 26 and 52 weeks
   g) increases the proportion of participants with undetectable HCV RNA at 26 and 52 weeks among those with chronic HCV infection at enrollment

   h) decreases HCV incidence at 52 weeks, for those who are HCV negative at enrollment

2) To evaluate whether 26 weeks of "one stop" integrated health services delivered in a mobile unit, supported by peer navigation, increases MOUD use, viral suppression, and PrEP use at 26 and 52 weeks compared to Enrollment

3) To evaluate whether 26 weeks of peer navigation to similar health services available at community-based agencies increases MOUD use, viral suppression, and PrEP use at 26 and 52 weeks compared to Enrollment

4) To document the prevalence of seropositivity for SARS-CoV-2 at baseline, 26 and 52 weeks

5) To document the impact of the COVID-19 epidemic on participants' experiences of seeking, obtaining and/or maintaining health services, housing, food security and drugs

6) To evaluate implementation of "one-stop" integrated health services using a mobile unit, supported by peer navigation, across study sites to identify mechanisms at multiple levels to:
   a) Guide real-time improvements and refinements in the conduct of the study to ensure primary and secondary outcomes are met with fidelity
   b) Examine the quality and process of services delivered in each study arm, particularly as these affect primary and secondary outcomes
   c) Develop evidence-based guidance for policymakers on the uptake and implementation of integrated health services using peer navigation and mobile health units in urban US regions to address HIV prevention in PWID
   d) Identify factors that enhance or impede the delivery of integrated health services using a mobile unit, supported by peer navigation, on primary and secondary outcomes, including responding to the impact of COVID-19 on service delivery

7) To use mathematical modeling methods to:
   a) Estimate the effect of integrated health services delivered in a mobile unit, supported by peer navigation, on reducing HIV incidence in PWID and their sexual and injection partners
   b) Estimate the cost-effectiveness of the integrated health services provided in a mobile unit and supported by peer navigation

Process:

For participants in the intervention arm, assess:
1. Time to provide MOUD treatment, ART (among those who are living with HIV and not on ART at Enrollment) and PrEP (among those who are without HIV at Enrollment)
2. The proportion of participants linked to community based MOUD, ART and PrEP services at 26 weeks

Exploratory Objectives:

Stored samples may be used to analyze HIV subtypes/strains, HIV drug resistance, and the duration of HIV infection. Phylogenetic methods may be used to evaluate behavioral, demographic, and clinical factors associated with viral clusters and transmission dynamics. Stored samples may also be used to characterize HCV strains and the relationship between HIV and HCV infections, and to explore issues related to COVID-19.

All proprietary rights reserved

Endpoints:

Primary:

Consistent with the primary study objective to evaluate whether the intervention improves use of MOUD and increases use of PrEP, as measured at 26 weeks among people without HIV, the following endpoints will be assessed:

1. Documented current use of MOUD. At the Week 26 visit:
   a) Alive
   b) Retained
   c) Biological evidence of MOUD (any detectable Methadone or Buprenorphine)
   d) A MOUD prescription current at the week 26 visit or proof of current receipt of MOUD from a clinic that does not provide individual MOUD prescriptions (e.g., methadone clinics)
2. Among participants who were without HIV at enrollment: alive, retained, without HIV, with detectable PrEP drugs (Truvada or Descovy in dried blood spot (DBS) samples, or Cabotegravir in plasma samples at the week 26 visit

Secondary:

1. To evaluate whether "one stop" integrated health services delivered in a mobile unit, supported by peer navigation, compared to peer navigation to similar health services available at community-based agencies, the following endpoint(s) will be assessed:

   a. The intervention improves use of MOUD at 52 weeks
      - Documented current use of MOUD: alive, retained, with biological evidence of MOUD (any detectable medications) at the week 52 visit and a MOUD prescription current at 52 weeks after enrollment or proof of current receipt of MOUD from a clinic that does not provide individual MOUD prescriptions (e.g., methadone clinics) at the week 52 visit
      - Documented use of MOUD during the study: a MOUD prescription documented during the 52 weeks of study follow-up or proof of current receipt of MOUD from a clinic that does not provide individual MOUD prescriptions (e.g., methadone clinics) during the 52 weeks of study follow-up

   b. The intervention increases rates of viral suppression among people living with HIV at 26 and 52 weeks
      - Among participants living with HIV at enrollment: alive, retained, and virally suppressed (VL <200 copies/mL) at the week 26 and week 52 visits, separately.

   c. The intervention increases use of PrEP among people without HIV at 26 and 52 weeks
      - Among participants without HIV at enrollment: alive, retained, HIV negative, with detectable PrEP drugs in DBS at the week 52 visit
      - Among participants without HIV at enrollment: alive, retained, HIV negative, with protective levels of PrEP drugs in DBS samples at the week 26 and 52 visits

   d. The intervention decreases opioid and polysubstance use at 26 and 52 weeks
      - Alive, retained, and no opioids (natural or synthetic), stimulants (methamphetamine, cocaine) or benzodiazepines detected in urine samples at week 26 and 52 visits

   e. The intervention decreases prevalence of bacterial STIs at 26 and 52 weeks
      - Alive, retained and no evidence of gonorrhea, chlamydia, or new or recurrent syphilis infection detected at the week 26 and 52 visits

f. The intervention reduces the rate of fatal and non-fatal overdose events by 26 and 52 weeks,
   - Death, with overdose as cause
   - Self-report of non-fatal overdose, collected at week 26 and 52 visits

g. The intervention increases the proportion of participants with undetectable HCV RNA among those with chronic HCV infection at enrollment
   - Undetectable HCV RNA at the week 26 and 52 visits among participants with chronic HCV at enrollment

h. The intervention reduces HCV incidence
   - HCV antibody positive at the week 52 visit among participants who are HCV antibody negative at enrollment

2. To evaluate whether 26 weeks of "one stop" integrated health services delivered in a mobile health delivery unit, supported by peer navigation, increases MOUD use, viral suppression, and PrEP use at 26 and 52 weeks compared to enrollment, the following endpoint(s) will be assessed:
   - In the intervention arm, change over time in the use of MOUD during the study, comparing documented use of MOUD (biological evidence of MOUD - any detectable medications - and a current MOUD prescription or proof of current receipt of MOUD from a clinic that does not provide individual MOUD prescriptions (e.g., methadone clinics)) at 26 and 52 weeks to documented use of MOUD at enrollment. MOUD use is assumed to be zero at enrollment due to study exclusion criteria. Follow-up endpoints will be defined as alive, retained, and having documented MOUD use.
   - Among participants living with HIV at enrollment: change over time in the proportion of people with viral suppression (VL<200 copies/mL), comparing 26 and 52 weeks to enrollment. Follow-up endpoints will be defined as alive, retained, and virally suppressed.
   - Among participants who were without HIV at enrollment: change over time in the proportion of people with detectable PrEP drugs in DBS at 26 and 52 weeks compared to enrollment. Follow-up endpoints will be defined as alive, retained, and having detectable PrEP.

3. To evaluate whether 26 weeks of peer navigation to similar health services available at community-based agencies increases MOUD use, viral suppression, and PrEP use at 26 and 52 weeks compared to enrollment, the following endpoint(s) will be assessed:
   - In the active control arm, change over time in the use of MOUD during the study, comparing documented use of MOUD (biological evidence of MOUD - any detectable medications - and a current MOUD prescription or proof of current receipt of MOUD from a clinic that does not provide individual MOUD prescriptions (e.g., methadone clinics)) at 26 and 52 weeks to documented MOUD use at enrollment. MOUD use is assumed to be zero at enrollment due to study exclusion criteria. Follow-up endpoints will be defined as alive, retained, and having documented MOUD use.
   - In the active control arm among participants living with HIV at enrollment: change over time in the proportion of people with viral suppression (VL<200 copies/mL), comparing 26 and 52 weeks to enrollment. Follow-up endpoints will be defined as alive, retained, and virally suppressed.
   - In the active control arm among participants who were without HIV at enrollment: change over time in the proportion of people with detectable PrEP drugs in DBS at 26

and 52 weeks compared to enrollment. Follow-up endpoints will be defined as alive, retained, and having detectable PrEP.

4. To assess the prevalence of SARS-CoV-2 seropositivity at baseline, 26 and 52 weeks, the following endpoint will be assessed:
   - Laboratory evidence of antibodies to SARS-CoV-2

The rest of the endpoints will be handled separately in a different SAP or will be added later.

2.3 Randomization

Random treatment-arm allocation will occur at a 1:1 ratio, stratified by study site and by HIV status. Permuted-block randomization will be used to assure balanced groups within each study site and HIV-status subgroup with 30 blocks per site/HIV-status stratum. A list of size 240 [10*(6+8+10)] would allow each site more than enough randomization slots in case a site is called upon to enroll greater than the projected sample size (approx. n=80 HIV negative participants and approx. n= 10 HIV positive participants per site), or in case of dropouts or randomization errors.

2.4 Blinding

This study is unblinded.

2.5 Sample Size and Power

Sample sizes are chosen to provide sufficient power for both the HIV-related and MOUD-related primary outcomes. By computing sample sizes based on each individual outcome separately and choosing the largest sample size needed for either outcome, high power is achieved for both individual outcomes. Prior work in PWID populations suggests that uptake of MOUD, ART, and PrEP in the control condition will likely be modest;[1,2] we anticipate 25% uptake of MOUD, 42.5% achieving viral suppression, and 5% uptake of PrEP in the control arm.

Sample sizes are computed to provide 90% power to detect a fifteen-percentage point difference in the proportion of participants achieving success, assuming a 5% type-I error rate (two-sided). A sample of 400 participants is needed to detect a 15-point difference in MOUD use (25% vs. 40%) between the active control and intervention arm at 26 weeks. Approximately 216 people without HIV would be needed to see a 15-point difference in PrEP uptake (5% vs. 20%). To cover both HIV and MOUD outcomes, 400 people without HIV will be needed. Approximately 10% of the study population is expected to be living with HIV – insufficient to accommodate a fully powered subgroup - but because this subgroup is expected to have very different rates of HIV-related success than the people without HIV subgroup, it will be important to analyze these participants separately. To assure 400 people without HIV, the target sample size will be augmented by 50 for a combined total of 450 participants, where 40-50 of those are expected to be people living with HIV.

2.6 Accrual and Retention

The study cohort (n=450) will be enrolled over the course of approximately 2.5 years and followed for 52 weeks each. This corresponds to a rate of approximately 15 new enrollees per month, or approximately 3 new enrollees per month at each of the five sites.

3. GENERAL DATA ANALYSIS CONSIDERATIONS

3.1 Analysis Set(s)

- **Intent to Treat (ITT):**
  All participants enrolled, excluding those who were inappropriately enrolled.

- **Safety:**
  There is no formal safety analysis in this study, however, serious adverse events (SAE) will be monitored for all randomized participants.

3.2 Statistical Analysis Issues

- **Missing Data:**
  In anticipation of the potential for missing data in this unstable study population, a missing-data strategy has been built into the primary endpoints. A positive result for each endpoint is defined as being "alive, retained," and meeting the MOUD, ART, or PrEP criteria at 26 weeks from enrollment. If the 26-week visit is missing for any individual, that individual is "not retained" at 26 weeks, and the 26-week result for that person will be considered "negative".

  Secondary endpoints are defined similarly. If missing data rates are higher than anticipated (over 15%) for primary or secondary endpoints, sensitivity analyses will be conducted to assess the impact of assuming missed visits represent negative outcomes.

  - **Missing Start and Stop Dates:**
    Start date:
    - If start date is completely missing, start date will not be imputed.
    - If year is present and month and day are missing, then set month and day to January 1 or date of enrollment whichever is later.
    - If year and day are present and month is missing, the set month to January or date of enrollment whichever is later.
    - If year and month are present and day is missing, set day to the 1st day of month or date of enrollment whichever is later.

    Stop date:
    - If end date is completely missing, end date will not be imputed.
    - If year is present and month and day are missing, then set month and day to December 31st or date of termination whichever is earlier.
    - If year and day are present and month is missing, set month to December or date of termination whichever is earlier.
    - If year and month are present and day is missing, set day to the last day of month.

- **Out of window visits:**
  For each required study visit, there is an allowable visit window specifying on which study days (post-enrollment) the visit is "allowed" to be completed. The allowable visit windows are contiguous between week 26 and week 52 and do not overlap. Within each allowable visit window, there is a target visit window and study visits should ideally be conducted within this window. Further details about visit windows are defined at section 6.8 of the Protocol V2.0.

  All data will be tabulated per the evaluation visit as recorded on the CRF even if the assessment is outside of the visit window. In data listings, actual visit dates will be presented alongside nominal visit types.

All proprietary rights reserved

4. INTERIM ANALYSIS AND DATA MONITORING COMMITTEE

No interim analyses are planned.

HPTN SMC oversight is planned for this study. The SMC will conduct interim reviews of study progress, including rates of participant accrual, visit retention, completion of primary and secondary endpoint collection, and, in a closed report, endpoint and safety data by arm. The frequency and content of SMC reviews will be determined prior to the start of the study and outlined in the SSP Manual but will occur at least once per calendar year.

5. GENERAL ANALYSIS METHODS

Participants characteristics at baseline will include the number of subjects with non-missing value (n), plus frequency and percentage for the categorical variables; and mean, median, standard deviation (SD), minimum (min), and maximum (max) for the continuous variables. Baseline is defined as Enrollment visit, except for data collected at screening and not at enrollment visit. For example, opioids data collected at screening visit will be considered as baseline data.

Any deviations from this statistical plan will be described in the final report.Primary and secondary outcomes will be limited to data collected within the allowable visit window for the week 26 and week 52 visits, respectively.

For all outcomes below, “retained” at 26 weeks is defined as having completed the 26-week visit within the allowable window of 126-308 days from enrollment, and “retained” at 52 weeks is defined as having completed the 52-week visit within the allowable window of 309-449 days from enrollment.

For MOUD outcomes, biological detection of Buprenorphine (or Norbuprenorphine or Buprenorphine Glucuronide) or Methadone (or EDDP) will be performed via laboratory analysis of plasma and urine samples. Detectable MOUD is defined as a value above the lower limit of quantitation (LLOQ) for any of the medications or metabolites mentioned above, in either plasma or urine.

For PrEP outcomes, the PrEP regimen tested (Truvada or Descovy in DBS, or CAB-LA in plasma) is determined based upon reported PrEP use. If there is no reported PrEP usage, the default regimen is Truvada. Detectable PrEP is defined as a value above the lower limit of quantitation (LLOQ) for the PrEP regimen tested.

All proprietary rights reserved

6. TRIAL PARTICIPANT DISPOSITION

6.1 Disposition of Participants

Final disposition for all study participants will be summarized using the following CONSORT flow chart.

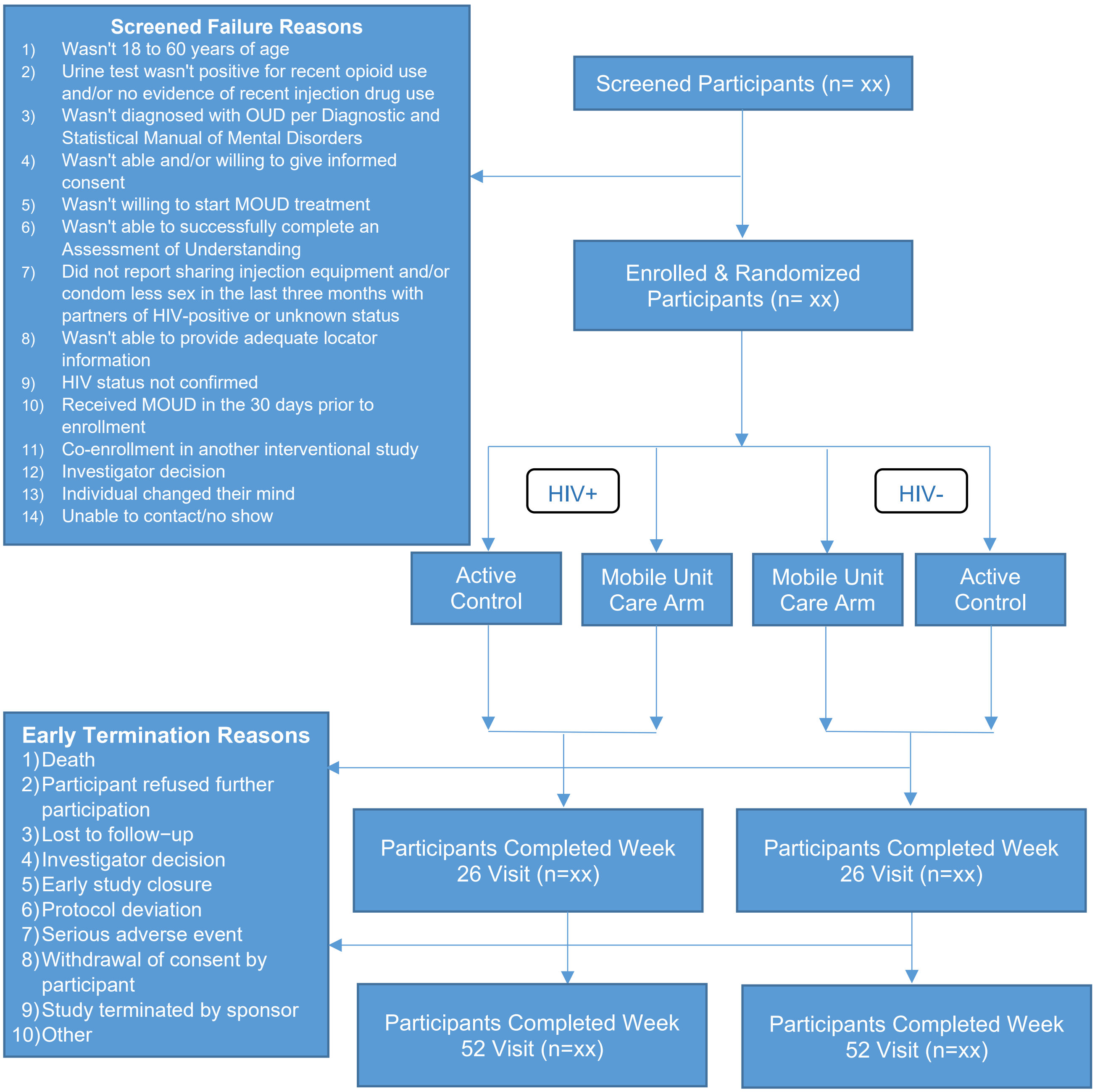

6.2 Protocol Deviations

Protocol deviations related to study inclusion or exclusion criteria, the conduct of the study, and inappropriate enrollment will be summarized by site, and overall, in the form of tables and listings. The following protocol deviations will be summarized:

- Inappropriate enrollment
- Failure to follow randomization or blinding procedures
- Conduct of non-protocol procedure
- Improper SAE
- Unreported SAE
- Breach of confidentiality
- Physical assessment deviation
- Lab assessment deviation
- Staff performing duties that they are not qualified to perform
- Use of non-IRB/EC-approved materials
- Informed consent process deviation
- Other

7. BASELINE DATA

Participants' baseline characteristics will be presented by site, HIV status, study arm, and overall.

- Demographics, including age, race, ethnicity, self-identified gender and sexual orientation.
- Drug use and overdose history including: the types of drugs taken (stimulants, opioids, other); ways the drugs were taken; and the number of days drugs were taken in the past month. Overdose history will be summarized by the type of drug leading to overdose on and the number of times overdosed.
- Delivery of the intervention will be described by summarizing the number of navigation sessions per participant, the location and length of the sessions, and the topics addressed.

8. EFFICACY/EFFECTIVENESS ANALYSES

Primary Outcomes:

Among the enrolled HIV-negative cohort:

- Documented current use of MOUD defined as: alive, retained, with biological evidence of MOUD (defined in section 5) and a current MOUD prescription or proof of current receipt of MOUD from a clinic that does not provide individual MOUD prescriptions (e.g., methadone clinics) at the week 26 visit

- Current use of PrEP defined as: alive, retained, HIV-negative, with detectable PrEP (defined in section 5) at the week 26 visit

For the primary outcome of MOUD use, a Generalized Linear Model (GLM) for binary outcomes using the identity link will be performed with treatment arm and site as covariates. The proportions of participants who meet the MOUD endpoint criteria (by-arm, and by-site-by-arm) at week 26 will be provided along with the estimate and 95% confidence interval for the difference in proportions.

For the primary outcome of PrEP use, a Generalized Linear Model (GLM) for binary outcomes using the identity link will be performed with treatment arm and site as covariates. The proportions of participants who meet the PrEP endpoint criteria (by-arm, and by-site-by-arm) at week 26 will be provided along with the estimate and 95% confidence interval for the difference in proportions.

9. SAFETY ANALYSES

In this study, the only drugs that will be dispensed or prescribed will be US FDA-approved medications for treatment or prevention of OUD, HIV, and STIs and prescriptions for contraceptives, primary care concerns, and chronic conditions, if indicated. There are no investigational products in this protocol. Therefore, there will be no monitoring or reporting of unanticipated treatment-related risks with the following exception: site investigators will report to the FDA any serious adverse events that are “unexpected” per the current version of the package insert for drugs provided for MOUD, ART or PrEP via the FDA’s MedWatch form, copying the RSC Safety Office.

9.1 Adverse Events and Deaths

Adverse events (AEs) that study staff become aware of will be recorded in source documentation and will be assessed for seriousness. Non-serious AEs will not be reported in the study database and referrals for care will be provided as necessary. Only Serious adverse events (SAEs) will be reported in form of tables and listings categorized by system organ class, severity grade (The Division of AIDS Table for Grading the Severity of Adult and Pediatric Adverse Events, Corrected Version 2.1, July 2017. https://rsc.niaid.nih.gov/clinical-research-sites/daids-adverse-event-grading-tables), HIV status and arm. Serious adverse events (SAEs) are those AEs that result in one or more of the following outcomes:

- Death
- A life-threatening (i.e., an immediate threat to life) event
- Requires in-patient hospitalization or prolongation of an existing hospitalization
- A persistent or significant incapacity or substantial disruption of the ability to conduct normal life functions
- A congenital anomaly/birth defect
- A medically important event that may not be immediately life-threatening or result in death or hospitalization but may jeopardize the patient or may require intervention to prevent one of the other outcomes listed in the definition above

Examples of SAEs that can be expected to occur during this study include deaths (e.g., overdoses or other addiction-related deaths) and life-threatening events requiring intervention (e.g., overdose reversals).

9.2 Other Safety Measures

Pregnancy will be presented in the form of listings by site, study arm and HIV-status.

10. SECONDARY ENDPOINTS

1. **To evaluate whether “one stop” integrated health services delivered in a mobile unit, supported by peer navigation, compared to peer navigation to similar health services available at community-based agencies, the following endpoint(s) will be assessed:**

   a) Improves use of MOUD at 52 weeks compared to the active control condition

All proprietary rights reserved

Outcomes:

- Documented current use of MOUD: alive, retained, with biological evidence of MOUD (as defined above) at the week 52 visit and a MOUD prescription current at 52 weeks after enrollment or proof of current receipt of MOUD from a clinic that does not provide individual MOUD prescriptions (e.g., methadone clinics) at the week 52 visit
- Documented use of MOUD during the study: a MOUD prescription documented during the 52 weeks of study follow-up or proof of current receipt of MOUD from a clinic that does not provide individual MOUD prescriptions (e.g., methadone clinics) during the 52 weeks of study follow-up

These outcomes will be modeled and summarized using the same strategy as specified for the primary outcome in section 8 above.

b) Increases rates of viral suppression among people enrolled living with HIV, at 26 and 52 weeks

Outcome:

- Among participants living with HIV at enrollment: alive, retained, and virally suppressed (VL <200 copies/mL) at the week 26 and 52 visits, separately.

The proportion of participants achieving the viral suppression outcome will be computed and presented by arm and within site by arm, along with exact Clopper-Pearson 95% confidence intervals using F-quantiles based on the Binomial distribution. No statistical comparisons between treatment arms will be made.

c) The intervention increases use of PrEP among people without HIV at 26 and 52 weeks

Outcome:

- Among participants without HIV at enrollment: alive, retained, HIV negative, with detectable PrEP drugs in DBS at the week 52 visit
- Among participants without HIV at enrollment: alive, retained, HIV negative, with protective levels of PrEP drugs in DBS samples at the week 26 and 52 visits

A protective level of oral PrEP[3] is defined as:
TFV-DP concentrations $\geq$ 800 fmol/punch in DBS among those tested for Truvada (TDF-FTC)
TFV-DP concentrations $\geq$ 950 fmol/punch in DBS among those tested for Descovy (TAF-FTC)

A protective level of long-acting PrEP (Cabotegravir) is defined as plasma concentrations above the four times the PA-IC_{90} of 166 ng/mL – that is, above 664 ng/mL.

These outcomes will be modeled and summarized using the same strategy as specified for the primary outcome in section 8 above.

d) Decreases opioid and polysubstance use at 26 and 52 weeks

Outcome:

- Opioids (natural or synthetic) detected in urine samples for those retained at the week 26 and 52 visits (visits analyzed separately).
- Opioids (natural or synthetic) detected, along with stimulants (methamphetamine, cocaine), xylazine and/or benzodiazepines detected in urine samples at the week 26 and 52 visits (visits analyzed separately).

This objective will be analyzed only in those giving a urine sample at the respective visit (week 26 or week 52).

Opioids (natural or synthetic) include opiates, fentanyls and synthetic opioids.

Opiate detection includes 6-mam, codeine, dihydrocodeine, heroin, hydrocodone, hydromorphone, morphine, morphine glucuronide naloxone, and normorphine.

Fentanyl detection includes 4-ANPP, acetyl fentanyl, alfentanil, fentanyl, FIBF, furanyl fentanyl, methoxyacetyl fentanyl, norfentanyl, para-chlorofentanyl, para-fluorofentanyl, and valeryl fentanyl.

Synthetic Opioid detection includes isotonitazene, meperidine, metonitazene, noroxycodone, oxycodone, oxymorphone, tapentadol, tramadol, and U-47700.

Stimulant detection includes amphetamine, MDA, MDEA, MDMA, methamphetamine, methylphenidate, phentermine, benzoylecgonine, cocaethylene, and cocaine.

Benzodiazepine detection includes alpha-hydroxyalprazolam, alprazolam, carisoprodol, chlordiazepoxide, clonazepam, diazepam, flurazepam, lorazepam, nordiazepam, oxazepam, and temazepam.

These outcomes will be modeled and summarized using the same strategy as specified for the primary outcome in section 8 above.

e) Decreases prevalence of bacterial STIs at 26 and 52 weeks

Outcome:

- Gonorrhea, chlamydia, or new or prevalent syphilis infection detected via local labs for those retained at the week 26 and 52 visits (visits analyzed separately).

This objective will be analyzed only in those giving a specimen and having a valid test result at the respective visit (week 26 or week 52).

Negative for syphilis, at any visit, is defined as a negative/not-detected non-treponemal test; or a positive non-treponemal test and a negative/not-detected treponemal test (this would be considered a false-positive test and thus considered negative for the visit).

Prevalent syphilis is defined as a positive non-treponemal test and a positive treponemal test, with a titer value >= 1:8.

Note: Participants can be non-prevalent (positive tests with tier < 1:8) but also not negative for syphilis. Thus, they had syphilis previously, but it is not a prevalent infection.

New syphilis is defined as a positive non-treponemal test and positive treponemal test (regardless of titer value) at a follow-up visit when they were negative at the previous visit.

These outcomes will be modeled and summarized using the same strategy as specified for the primary outcome in section 8 above.

f) Decreases fatal and non-fatal overdose events by 26 and 52 weeks

Outcomes:

- Death, with overdose as cause, collected on or before 26- and on or before 52-week visits, separately.
- Self-report of non-fatal overdose, collected on or before week 26- and on or before 52-week visits, separately.

These outcomes are reported on the SAE log form.

Death, caused by overdose, will be modeled with a time-to-event cox proportional hazards model with treatment arm as a covariate.

Person-time for fatal overdoses will be calculated as follows:

- For those retained (alive) at the visit: time between enrollment and the week 26 or week 52 visit date.
- For those reported deceased by the visit date: time between enrollment and recorded date of death.
- If deceased and no recorded date of death: time between enrollment and the midpoint of last attended visit and termination date.
- Those with no reported death and not retained at week 26 and/or week 52 (or terminated before the visit): use time between enrollment and the last attended visit (or termination date). If no other visit besides enrollment is documented: exclude from the analysis.

Note: Last attended visit can include the enrollment or week 26 visits, an interim visit, or a navigation session

Self-reported non-fatal overdoses will use a Poisson regression model (GLM with log link) with treatment arm as a covariate. We will use the number of reported non-fatal overdoses in the 30 days prior to the respective visit to compare treatment arms.

g) Increases the proportion of participants with undetectable HCV RNA at 26 and 52 weeks among those with chronic HCV infection at enrollment

Outcome:

- Undetectable HCV RNA at the week 26 and 52 visits (visits analyzed separately) among participants with chronic HCV at enrollment.

The HCV treatment outcome will be modeled and summarized using the same strategy as specified for the primary outcome in section 8 above.

h) Decreases HCV incidence at 52 weeks, for those who are HCV negative at enrollment

Outcome:

- HCV antibody positive at the week 52 visit among participants who are HCV antibody negative at enrollment.

HCV incidence will be modeled using Poisson regression (GLM with log link), with treatment arm and site as covariates and person years as an offset. Person time will be computed as the number of years from enrollment to the visit date where infection is first detected for participants with incident HCV infection, and from enrollment to the week 52 visit date (or last available visit) for HCV-negative participants with no incident infection.

2. **To evaluate whether 26 weeks of "one stop" integrated health services delivered in a mobile health delivery unit, supported by peer navigation, increases MOUD use, viral suppression, and PrEP use at 26 and 52 weeks compared to enrollment, the following endpoint(s) will be assessed:**

- In the intervention arm, change over time in the use of MOUD during the study, comparing documented use of MOUD (biological evidence of MOUD - any detectable medications - and a current MOUD prescription or proof of current receipt of MOUD from a clinic that does not provide individual MOUD prescriptions (e.g., methadone clinics)) at 26 and 52 weeks to documented use of MOUD at enrollment. MOUD use is assumed to be zero at enrollment due to study exclusion criteria. Follow-up endpoints will be defined as alive, retained, and having documented MOUD use.
- Among participants living with HIV at enrollment: change over time in the proportion of people with viral suppression (VL<200 copies/mL), comparing 26 and 52 weeks to enrollment. Follow-up endpoints will be defined as alive, retained, and virally suppressed.

- Among participants who were without HIV at enrollment: change over time in the proportion of people with detectable PrEP drugs in DBS at 26 and 52 weeks compared to enrollment. Follow-up endpoints will be defined as alive, retained, and having detectable PrEP.

The viral suppression outcome will not be modelled. We will report the proportions at enrollment, 26 week and 52-week visits, along with exact Clopper-Pearson 95% confidence intervals using F-quantiles based on the Binomial distribution.

Change over time in the MOUD endpoint will be modeled using GEE (one model for 26 weeks compared to enrollment, and another model for 52 weeks compared to enrollment) with a linear link and an exchangeable correlation structure accounting for within-person repeated measures over time. Each model will include an indicator for time point (26 weeks vs enrollment, or 52 weeks vs enrollment) and a covariate for site. The proportion of participants achieving the outcome at each time point will be summarized, along with the estimated difference in proportions and corresponding 95% confidence interval.
Change over time in the PrEP outcome will be modeling and summarized using the same strategy as defined above for MOUD.

3. **To evaluate whether 26 weeks of peer navigation to similar health services available at community-based agencies increases MOUD use, viral suppression, and PrEP use at 26 and 52 weeks compared to enrollment, the following endpoint(s) will be assessed:**

- In the active control arm, change over time in the use of MOUD during the study, comparing documented use of MOUD (biological evidence of MOUD - any detectable medications - and a current MOUD prescription or proof of current receipt of MOUD from a clinic that does not provide individual MOUD prescriptions (e.g., methadone clinics)) at 26 and 52 weeks to documented MOUD use at enrollment. MOUD use is assumed to be zero at enrollment due to study exclusion criteria. Follow-up endpoints will be defined as alive, retained, and having documented MOUD use.
- In the active control arm among participants living with HIV at enrollment: change over time in the proportion of people with viral suppression (VL<200 copies/mL), comparing 26 and 52 weeks to enrollment. Follow-up endpoints will be defined as alive, retained, and virally suppressed.
- In the active control arm among participants who were without HIV at enrollment: change over time in the proportion of people with detectable PrEP drugs in DBS at 26 and 52 weeks compared to enrollment. Follow-up endpoints will be defined as alive, retained, and having detectable PrEP.

The viral suppression outcome will not be modelled. We will report the proportions at enrollment, 26 week and 52-week visits, along with 95% confidence intervals using exact binomial distribution.

For participants in the control arm, change over time in the MOUD and PrEP outcomes will be modeled and presented using the same strategy as defined in section 2 above for the intervention arm.

4. **To document the prevalence of seropositivity for SARS-CoV-2 at baseline, 26 and 52 weeks, the following endpoint(s) will be assessed:**

- Laboratory evidence of antibodies to SARS-CoV-2 Overall and by Site (and by arm).

At each of the three timepoints, we will report the proportion of participants with lab evidence of SARS-CoV-2 antibodies (by-arm and by-site-by-arm) at week 26 and week 52. Laboratory evidence of antibodies to SARS-CoV-2

Analyses for Secondary Objectives 5, 6, and 7, along with Process Objectives 1 and 2, will be outlined in a different Analysis Plan document.

11. REFERENCES

1. Miller WC, Hoffman IF, Hanscom BS, et al. A scalable, integrated intervention to engage people who inject drugs in HIV care and medication-assisted treatment (HPTN 074): a randomised, controlled phase 3 feasibility and efficacy study. Lancet 2018; 392(10149): 747-59.

2. Morgan JR, Schackman BR, Leff JA, Linas BP, Walley AY. Injectable naltrexone, oral naltrexone, and buprenorphine utilization and discontinuation among individuals treated for opioid use disorder in a United States commercially insured population. J Subst Abuse Treat 2018; 85: 90-6.

3. HPTN LC Pharmacology Core (Marzinke, Anderson).

12. CHANGE HISTORY

Version		Affected Section(s)	Activity Description
Number	Effective Date		
v1.0	14JUL2023	N/A	First version
v2.0	Date of last signature	2.2, 5, 8, 10, 11, 12	Defining cohorts and endpoints, including biological evidence of MOUD, more clearly. Updating general analysis methods with definitions of endpoints including PrEP regimen testing. Updating protective levels of PrEP for Truvada, Descovy and CAB-LA (with references added).

All proprietary rights reserved
