# Supplementary material for: Mobile Units for Integrated Addiction and HIV Prevention Services for People Who Inject Drugs: The HPTN 094 Randomized Clinical Trial
Source: JAMA Psychiatry. 2026 Nov 4:e263644. Online ahead of print. doi: 10.1001/jamapsychiatry.2026.3644 (PMC13735193; doi:10.1001/jamapsychiatry.2026.3644)
Supplement: Supplementary file 2 [file jamapsychiatry-e263644-s002.pdf]

Supplemental Online Content

Shoptaw S, El-Bassel N, Hanscom B, et al. Mobile Units for Integrated Addiction and HIV Prevention Services for People Who Inject Drugs: The HPTN 094 Randomized Clinical Trial. *JAMA Psychiatry*. Published online November 4, 2026. doi:10.1001/jamapsychiatry.2026.3644

eTable 1. Overview of Medical Care Provided in the Mobile Unit for Intervention Arm Participants
eAppendix 1. Description of limited medical care, HIV testing, PrEP provision, PrEP adherence assessments, and MOUD and other drug use
eAppendix 2. Links to Peer Navigation Manual and the Graphical Peer Navigation Manual
eTable 2. Cumulative all−cause mortality through week 26 and 52, overall and by site

This supplemental material has been provided by the authors to give readers additional information about their work.

eTable 1. Overview of Medical Care Provided in the Mobile Unit for Intervention Arm Participants

This table describes the scope of clinical services provided in the integrated strategies approach on the mobile unit.<br><br>This is not a comprehensive list of services provided by clinicians in the mobile unit, as clinicians may encounter and respond to many conditions in the course of providing care for addiction, infectious diseases and primary care. The descriptions below set expectations for how primary care clinicians can function in the mobile unit with consistency from site to site, with limits on what will be provided, while enabling them to provide needed attention to the conditions most common to primary care. Referrals will be provided for diagnosis and maintenance of care of most chronic primary care conditions. Referrals will also be provided for conditions that will routinely be managed in the mobile unit (e.g. STI infection) if they require more specialized care (e.g. neurosyphilis).	
**Condition**	**Notes**
OUD	MOUD will be managed on the mobile unit. Will include dispensation of drugs (pending donation), to include:<br>• Buprenorphine-based medicine (sublingual and injectable regimens)<br>• Long-acting naltrexone<br>Participants who prefer methadone will be referred to community-based services if available
Stimulant Use	Participants who also use stimulants (methamphetamine, cocaine) will be referred to 12-step meetings such as crystal meth anonymous, narcotics anonymous and alcoholics anonymous, and evidence-based behavioral treatment, where available.
HIV-PrEP	PrEP will be managed from the mobile unit including:<br>• Dispensation of one, single pill regimen<br>• Prescription provided for fulfillment at a pharmacy if a different regimen is indicated
HCV Treatment	• Testing will be performed as part of study procedures.<br>• Those who test positive will be referred for treatment.
STI	Bacterial STIs will be managed from the mobile unit including:<br>• Provision of antibiotics for treatment of bacterial STIs tested for as part of the protocol (CT, GC and syphilis)<br>• Providing a prescription for fulfillment at a pharmacy, or a referral to community-available services, for any conditions not able to be treated on the mobile unit
HAV	• Testing will be performed as part of study procedures.<br>• Those without evidence of immunity will be referred for vaccination.
HBV	• Testing will be performed as part of study procedures.<br>• Those without evidence of immunity will be referred for vaccination.<br>• Those with evidence of chronic infection who are HIV positive may be co-treated by an appropriate ART regimen dispensed on the mobile unit or through a prescription fulfilled at a local pharmacy.<br>• Those with HBV who are HIV negative will be counseled on the risks/benefits of PrEP.
Mental Health	Clinicians will screen for symptoms of mental health disorders during visits on the mobile unit.<br>• For any issues identified, participants will be referred for further evaluation and/or care at community-based services, facilitated by peer navigation.

© 2026 Shoptaw S et al. *JAMA Psychiatry*.

	• If a person appears to be at risk to harm themselves or others, 911 will be called
Pregnancy	Pregnant participants may utilize the mobile unit.<br>• Participants who are pregnant will continue to be seen for MOUD, ART, PrEP and other services on the mobile unit. all pregnant participants will be referred for obstetric care with an OB/GYN provider comfortable treating pregnant women who inject drugs treated with MOUD. Study clinicians will endeavor to coordinate with the obstetric care provider to optimize care.
Reproductive Health	Limited reproductive health services will be provided in the mobile unit<br>• Prescriptions for oral contraception<br>• Condoms/lube
Basic Primary Care	Basic primary care that will be provided on the mobile unit will include:<br>• Diagnosis and management of minor acute illnesses and infections such as upper respiratory tract infections, colds, flu, diarrheal illness<br>• Providing prescriptions for pharmacy fulfillment for such conditions if indicated.<br>• Identifying more complex care needs and referring for further diagnostics and care from community-available services
COVID-19	Clinicians will assess participants for COVID-19 at each encounter. Those with suspected COVID-19 will be referred for further evaluation, care and treatment, as appropriate and available. CDC and local guidelines for discontinuation of isolation will direct when participants with suspected COVID-19 can resume in-person visits. Distance procedures to collect data and monitor health will be implemented to the extent possible.
More Complex Care Needs & Chronic Conditions	• Management of complex care needs or chronic conditions will be referred to community-available services, such as FQHCs or other clinics<br>• Clinicians on the mobile unit may provide prescriptions for medications for chronic conditions that have been lost or stolen or are needed for continuity of care, including communication and coordination, as possible, with the provider managing the care of the chronic condition.

© 2026 Shoptaw S et al. *JAMA Psychiatry*.

eAppendix 1. Description of limited medical care, HIV testing, PrEP provision, PrEP adherence assessments, and MOUD and other drug use

Procedures for limited medical care at study visits: Sexually transmitted infections (STI) testing was performed at the study sites for *Neisseria gonorrhoeae* and *Chlamydia trachomatis* using nucleic acid amplification tests for urine and swabs (throat, rectal). Participants were also tested for syphilis using blood specimens. Treatment (or referral) for symptomatic or asymptomatic cases of confirmed STIs was provided. Participants who tested positive for Hepatitis C virus (HCV) or who presented at the study visit with complex medical problems were referral to treatment (if available). Access to harm reduction services and supplies were available as part of limited primary care at study visits.

HIV testing: HIV testing was performed at study sites; results were confirmed at the HIV Prevention Trials Network (HPTN) Laboratory Center (Baltimore, MD). Viral load testing was performed retrospectively using the Aptima HIV-1 Quant Dx assay (Hologic, Marlborough, MA).

Procedures for provision of PrEP: Provision (or prescription) (tenofovir alafenamide-emtricitabine [Gilead Sciences] for male participants; tenofovir disoproxil fumarate-emtricitabine ([Gilead Sciences]) for female participants); immediate access in the mobile unit or facilitated linkage to area providers for antiretroviral therapy (ART) for people living with HIV (PWH) (tenofovir alafenamide-emtricitabine-bictegravir [Gilead Sciences]). Long-acting HIV PrEP formulations were not provided by the study.

PrEP adherence assessments: Intraerythrocytic tenofovir-diphosphate (TFV-DP) concentrations for participants on tenofovir (TFV)-based pre-exposure prophylaxis (PrEP; emtricitabine/tenofovir disoproxil fumarate, F/TDF; emtricitabine/tenofovir alafenamide, F/TAF) or evidence (biomedical or injection administration data) for participants on long-acting cabotegravir (CAB-LA) were used to objectively evaluate adherence to PrEP regimens. Intraerythrocytic TFV-DP and CAB-LA testing was performed at the HPTN Laboratory Center.

Intraerythrocytic TFV-DP concentrations were determined from dried blood spots (DBS) via liquid chromatographic-tandem mass spectrometric (LC-MS/MS) analysis on API 6500 (SCIEX, Redwood City, CA); the assay lower limit of quantitation is 6.25 fmol/punch. Specific processing workflows and punch sizes were used for participants offered F/TDF (3 mm punch) or F/TAF (2 x 7 mm punches). Of note, emtricitabine-triphosphate (FTC-TP) was also monitored, but TFV-based PrEP use was based on TFV-DP measurements.

For participants who sought out care outside the study for CAB-LA PrEP, plasma CAB measurements were conducted via LC-MS/MS, as previously described, with a lower limit of quantitation of 25 ng/mL (1). For participants who reported CAB−LA use, plasma concentrations above the four-times protein adjusted 90% inhibitory concentration (4x PA-IC90; 664 ng/mL) was the target concentration associated with protection (2).

MOUD and other drug use: Buprenorphine was available in the mobile unit. Methadone treatment was available by facilitated referral to narcotic treatment programs located in community, as methadone was not available on the mobile unit.

Within the HPTN Laboratory Center, liquid chromatography-high resolution mass spectrometry (LC-HRMS) with MS2 library matching (Thermo Fisher Scientific, Waltham, MA) was used for the qualitative identification of the following analytes in urine samples collected during study visits: 6-monoacetylmorphine, α-hydroxyalprazolam, alprazolam, amphetamine, benzoylecgonine (cocaine metabolite), buprenorphine, buprenorphine-glucuronide, carisoprodol, chlordiazepoxide, cis-tramadol, clonazepam, cocaine, codeine, diazepam, dihydrocodeine, EDDP (2-ethylidene-1,5-dimethyl-3,3-diphenylpyrrolidine), ethyl glucuronide, fentanyl, flurazepam, hydrocodone, hydromorphone, lorazepam, MDA (3,4-methylenedioxyamphetamine), MDEA (3,4-methylenedioxy-N-ethylamphetamine), MDMA (3,4-methylenedioxymethamphetamine), meperidine, methadone, methamphetamine, methylphenidate, morphine, naloxone, nitazene, norbuprenorphine, nordiazepam, norfentanyl, noroxycodone, oxazepam, oxycodone, oxymorphone, phentermine, tapentadol, temazepam, THC-COOH (11-nor-9-carboxy-delta-9-tetrahydrocannabinol), xylazine, and zolpidem. Analyte limits of detection ranged from 5 ng/mL to 200 ng/mL.

© 2026 Shoptaw S et al. *JAMA Psychiatry*.

A biomarker confirmed methadone testing was determined using LC-derived urine measurements. Plasma samples were used to assess adherence to buprenorphine. Buprenorphine and its major metabolite norbuprenorphine, and methadone and its metabolite EDDP (2-ethylidene-1,5-dimethyl-3,3-diphenylpyrrolidine) were measured via liquid chromatographic-tandem mass spectrometric (LC-MS/MS analysis on an Altis Plus mass analyzer (Thermo Fisher Scientific); for all analytes, the lower limit of quantitation was 5 ng/mL.

REFERENCES

1. Landovitz RJ, Li S, Eron JJ, Grinsztejn B, Dawood H, Liu AY, et al. Tail-phase safety, tolerability, and pharmacokinetics of long-acting injectable cabotegravir in HIV-uninfected adults: a secondary analysis of the HPTN 077 trial. Lancet HIV 2020;7:e472–81.
2. Marzinke MA, Grinsztejn B, Fogel JM, Piwowar-Manning E, Li M, Weng L, McCauley M, Cummings V, Ahmed S, Haines CD, Bushman LR, Petropoulos C, Persaud D, Adeyeye A, Kofron R, Rinehart A, St Clair M, Rooney JF, Pryluka D, Coelho L, Gaur A, Middelkoop K, Phanuphak N, Cohen MS, Hendrix CW, Anderson P, Hanscom B, Donnell D, Landovitz RJ, Eshleman SH. Characterization of Human Immunodeficiency Virus (HIV) Infection in Cisgender Men and Transgender Women Who Have Sex With Men Receiving Injectable Cabotegravir for HIV Prevention: HPTN 083. J Infect Dis. 2021 Nov 16;224(9):1581-1592.

© 2026 Shoptaw S et al. *JAMA Psychiatry*.

eAppendix 2. Links to Peer Navigation Manual and the Graphical Peer Navigation Manual

The Peer Navigation Manual is published online and can be downloaded for review from this URL: https://www.hptn.org/sites/default/files/inlineAppendixs/094-PeerNavigationManual_27Apr2021.pdf

The Graphical Peer Navigation Manual is published online and can be downloaded for review from this URL: https://www.hptn.org/sites/default/files/inlineAppendixs/094-NavigationGuide_06March2024.pdf

© 2026 Shoptaw S et al. *JAMA Psychiatry*.

eTable 2. Cumulative all−cause mortality through week 26 and 52, overall and by site

	Active Control Arm (n=206) Deaths/100PYs, IR (95% CI)	Intervention Arm (n=203) Deaths/100PYs, IR (95% CI)	Estimated Hazard Ratio (95% CI)[2]	p-value[2]
		Week 26		
All Sites	9, 9.5 (4.3, 18.0)	3, 3.0 (0.6, 8.9)	0.33 (0.09, 1.23)	0.100
New York, Bronx	1, 5.7 (0.1, 31.9)	0, 0.0 (0.0, 19.9)		
Los Angeles	3, 12.7 (2.6, 37.1)	1, 4.8 (0.1, 26.8)		
Washington DC	1, 11.9 (0.3, 66.2)	0, 0.0 (0.0, 41.5)		
Houston	3, 13.1 (2.7, 38.2)	1, 4.0 (0.1, 22.4)		
Philadelphia	1, 4.4 (0.1, 24.7)	1, 3.9 (0.1, 21.7)		
		Week 52		
All Sites	13, 7.3 (3.9, 12.5)	4, 2.1 (0.6, 5.5)	0.29 (0.10, 0.90)	0.032
New York, Bronx	2, 6.3 (0.8, 22.6)	1, 3.4 (0.1, 18.8)		
Los Angeles	4, 9.1 (2.5, 23.3)	1, 2.2 (0.1, 12.5)		
Washington DC	1, 6.7 (0.2, 37.4)	0, 0.0 (0.0, 23.4)		
Houston	3, 7.9 (1.6, 23.0)	1, 2.3 (0.1, 12.7)		
Philadelphia	3, 6.2 (1.3, 18.1)	1, 1.9, (.0, 10.6)		

[1] Deaths = the number of deaths (all causes), PYRS = observed person years, IR = incidence rate, events per 100 person years, exact 95% Poisson confidence interval
[2] Cox Proportional Hazards Model for differences by Arm for All Sites

© 2026 Shoptaw S et al. *JAMA Psychiatry*.
