# Supplementary material for: Mobile Units for Integrated Addiction and HIV Prevention Services for People Who Inject Drugs: The HPTN 094 Randomized Clinical Trial
Source: JAMA Psychiatry. 2026 Nov 4:e263644. Online ahead of print. doi: 10.1001/jamapsychiatry.2026.3644 (PMC13735193; doi:10.1001/jamapsychiatry.2026.3644)
Supplement: Supplementary file 3 [file jamapsychiatry-e263644-s003.pdf]

Data Sharing Statement

Shoptaw. Mobile Units for Integrated Addiction and HIV Prevention Services for People Who Inject Drugs. *JAMA Psychiatry*. Published November 04, 2026.
doi:10.1001/jamapsychiatry.2026.3644

Data
Additional Information: Clinical Trials.gov (NCT04804072)
Data available: Yes
Data types: Deidentified participant data, Data dictionary
How to access data: At present, the data depository is being built at the Statistical Center for HIV/AIDS Research, Fred Hutchinson Cancer Center, Seattle, WA, USA. The scientist in charge of the data repository is Brett Hanscom: bhanscom@fredhutch.org The Protocol Co-Chairs for the study also can be contacted to request data sharing from this study: Steve Shoptaw: sshoptaw@mednet.ucla.edu Nabila El-Bassel: ne5@columbia.edu
When available: With publication

Supporting Documents
Document types: Other (please specify)
Additional Information: Goodman-Meza D, Shoptaw S, Hanscom B, Smith LR, Andrew P, Kuo I, Lake JE, Metzger D, Morrison EAB, Cummings M, Fogel JM, Richardson P, Harris J, Heitner J, Stansfield S, El-Bassel N; HPTN 094 Study Team. Delivering integrated strategies from a mobile unit to address the intertwining epidemics of HIV and addiction in people who inject drugs: the HPTN 094 randomized controlled trial protocol (the INTEGRA Study). Trials. 2024 Feb 15;25(1):124. doi: 10.1186/s13063-023-07899-5. PMID: 38360750; PMCID: PMC10870682.
How to access documents: The documents will be available at the data depository at the Statistical Center for HIV/AIDS Research, Fred Hutchinson Cancer Center, Seattle, WA, USA. Dr. Brett Hanscom is the custodian of this repository: bhanscom@fredhutch.org
When available: With publication

Additional Information
Who can access the data: Data will be made available to any investigator who receives IRB approval for their proposed data analysis. A data dictionary will be available. All requests must be accompanied by a set of specific aims for the analyses proposed. These requests will be reviewed and approved by Drs. Hanscom, Shoptaw, and El-Bassel.
Types of analyses: Analyses conducted can be dedicated to answer a range of research questions. As noted, All requests for additional analyses must be accompanied by a set of specific aims and an intended purpose (i.e., whichever media the findings will be published in).
Mechanisms of data availability: These data will be available after approval of a proposal and will be provided with a signed data use agreement (DUA).
